# A cyclic adenosine monophosphate response element-binding protein inhibitor enhances the antibacterial activity of polymyxin B by inhibiting the ATP hydrolyzation activity of CrrB

**DOI:** 10.3389/fphar.2022.949869

**Published:** 2022-09-06

**Authors:** Wei Huang, Jinyong Zhang, Yuzhang He, Chunxia Hu, Shumin Cheng, Huan Zeng, Manling Zheng, Huijuan Yu, Xue Liu, Quanming Zou, Ruiqin Cui

**Affiliations:** ^1^ Antimicrobial Drug Screening Laboratory, Shenzhen Institute of Respiratory Diseases, Shenzhen People’s Hospital (The Second Clinical Medical College, Jinan University, The First Affiliated Hospital, Southern University of Science and Technology), Shenzhen, China; ^2^ Department of Clinical Microbiology, Shenzhen People’s Hospital (The Second Clinical Medical College, Jinan University, The First Affiliated Hospital, Southern University of Science and Technology), Shenzhen, China; ^3^ National Engineering Research Center of Immunological Products, Department of Microbiology and Biochemical Pharmacy, College of Pharmacy, Third Military Medical University, Chongqing, China; ^4^ Department of Pathogen Biology, International Cancer Center, Shenzhen University Health Science Center, Shenzhen, China; ^5^ College of Pharmacy, Jinan University, Guangzhou, China; ^6^ Medical College, Shantou University, Shantou, China

**Keywords:** antibiotic adjuvant, *Klebsiella pneumoniae*, polymyxin B resistant, lipid A, lipopolysaccharide, two-component system

## Abstract

The emergence of polymyxin B (PB) resistant Gram-negative bacteria poses an important clinical and public health threat. Antibiotic adjuvants development is a complementary strategy that fills the gap in new antibiotics. Here, we described the discovery of the enhancement capacity of compound 666-15, previously identified as an inhibitor of cyclic adenosine monophosphate response element-binding protein (CREB), on the activity of PB against *Klebsiella pneumoniae in vitro* and *in vivo*. Mechanistic studies showed that this compound reduced the transcription and translation levels of genes related to lipid A modification in the presence of PB. We also identified that 666-15 reduces the ATP hydrolyzation activity of CrrB, and P151L mutation mediates the resistance of bacteria to the enhancement of 666-15. Our results demonstrated the potential of 666-15 in clinical application and support the further development of a PB synergist based on this compound.

## Introduction

Polymyxins are one of the primary classes of antibiotics with activity against most Gram-negative bacteria. Two polymyxins, colistin and polymyxin B (PB), differ by a single amino acid in the peptide ring, with phenylalanine in PB and leucine in colistin (Nation et al., 2014). PB is recommended as the preferred agent for routine systemic use in invasive infections, while colistin is recommended in the treatment of lower urinary tract infections given a renal clearance of the prodrug colistin methanesulfonate that then converts to the active moiety colistin in the urinary tract (Tsuji et al., 2019).

In many areas where multidrug-resistant (MDR) Gram-negative bacteria are prevalent, colistin and PB are being considered last-resort antibiotics in clinical medicine. However, toxicity and polymyxin resistance (PR) are two important factors hindering the use of polymyxins. Nephrotoxicity and neurotoxicity are two major adverse effects of colistin. Pharmacokinetic–pharmacodynamic (PK-PD) analyses have shown that the risk of colistin-associated nephrotoxicity increases with plasma concentrations above 2.5–3 µg/ml (Sorli et al., 2013). Paresthesia is the most common neurotoxicological effect and occurs in 27% of patients (Falagas and Kasiakou, 2006). Modification of lipopolysaccharide (LPS), which is the initial target of polymyxins, specifically lipid A modification, is the most common molecular mechanism of polymyxin resistance. The charge of LPS is increased by the addition of phosphoethanolamine (pEtN) and/or 4-amino-4-deoxy-L-arabinose (L-Ara4N) cationic groups, and therefore blocks polymyxin binding (Jiang et al., 2010; Aquilini et al., 2014; Lin et al., 2014). LPS modifications are achieved by enzymes that are encoded by a large panel of genes, such as the *pmrHFIJKLM* operon (also called the *arnBCADTEF* or *pbgPE* operon) (Raetz et al., 2007). Furthermore, the qualitative modification of LPS is also regulated by two-component systems, such as PmrA/B, PhoP/Q, and CrrA/B (Gunn, 2008; Dalebroux and Miller, 2014; Wright et al., 2015). Moreover, plasmid-mediated forms of PR (mediated by *mcr* genes) have been reported since 2015 (Liu et al., 2016). For these reasons, if polymyxins cannot be used due to drug resistance or side effects, there will be no cure for some infections caused by MDR Gram-negative bacteria.

With no alternative medicine available, an infection caused by PR strains is a clinical challenge. In this case, there are two ways to address the current dilemma. One is to develop new antibacterial agents with completely new structures and modes of action but the success rate of that approach is often very low (Payne et al., 2007; Tommasi et al., 2015). Another complementary strategy is to develop antibiotic adjuvants that enhance the activity of current drugs and block the development of resistance (Gill et al., 2015; Wright, 2016). In this study, we initiated a screening program for PB synergists, and described the discovery of the enhancement capacity of compound 666-15, previously identified as an inhibitor of cyclic adenosine monophosphate response element-binding protein (CREB), on the activity of PB against *K. pneumoniae* (Xie et al., 2015). Mechanistic studies showed that this compound reduced bacterial lipid A modification levels by inhibiting the activity of CrrB.

## Materials and methods

### Reagents

Luria–Bertani (LB) broth powder was purchased from Meilunbio (Dalian, China). 666-15 was purchased from Topscience (Shanghai, China). Antibiotics were purchased from MedChem Express (MCE, United States). Thiazolyl blue tetrazolium bromide (MTT) and phosphate-buffered saline (PBS) were purchased from Sangon Biotech (Shanghai, China). The bacterial total RNA extraction kit and DNA extraction kit were purchased from TIANGEN (Beijing, China). RT Master Mix and SYBR Green qPCR Master Mix were purchased from MedChem Express (MCE, United States). The lipopolysaccharide (LPS) detection kit was purchased from Cloud-Clone (Wuhan, China). Propidium iodide (PI) was purchased from Thermo Fisher Scientific, USA. 3,3-Dipropylthiadi-carbocyanine iodide [DiSC_3_(5)] was purchased from AAT Bioquest, USA. The LIVE/DEAD BacLight bacterial viability kit was purchased from Invitrogen, USA.

### Bacterial strains and growth conditions

Strains were obtained from the American Type Culture Collection (Manassa, VA, United States). Clinical strains were collected by Shenzhen People’s Hospital. Strains were grown at 37 C in LB broth or on LB agar plates. The details of the strains were provided in Supplementary Table S1.

### Minimum inhibitory concentration and fraction inhibitory concentration index determination

Bioactive Compound Library (4724 molecules) was purchased from Selleck Chemicals (Houston, TX, United States). Antibiotics were purchased from MedChem Express (MCE, United States). MIC determination was performed by a standard broth microdilution method. Briefly, the compound was serially diluted two-fold in bacterial solution (OD_600_ = 0.001) and then inoculated into a transparent 96-well plate. MTT was used for measuring bacterial viability after 18 h. The MIC values were determined as the lowest concentration of compounds at which 90% bacterial growth was inhibited.

The Chequerboard strategy was used for FICI determination. FICI between two compounds, A and B, is calculated from the following formula:

$$FICI=\frac{MIC\;\left(A\;in\;combination\;with\;B\right)}{MIC\;\left(A\;alone\right)}+\frac{MIC\;\left(B\;in\;combination\;with\;A\right)}{MIC\;\left(B\;alone\right)}.$$

Synergy was defined as a FICI value ≤ 0.5 (Odds, 2003).

### Spontaneous mutant selection

*K. pneumoniae* ATCC13883 cells were used to raise spontaneous mutant selection. An aliquot of bacteria (30 µl) at the mid-log phase was added to the fresh LB medium (3 ml) with PB (0.25 µg/ml) and 666-15 (0.5 µg/ml). Ten independent experiments were performed. The cultures with growth were transferred to a fresh LB medium with PB (0.25 µg/ml) and 666-15 (1 µg/ml) for further culture. This process (a serial increase of 666-15) was continued until growth was no longer available. The cells from the highest concentration that supported growth were spread onto LB agar plates. Single colonies were isolated and inoculated to a fresh LB medium. The FICI between PB and 666-15 of purified strains was measured by chequerboard strategy. Strains with a shift of FICI values were subjected to sequencing.

### CRISPR-Cas9 gene editing

The two plasmid systems pSGKp-Spec and pCasKp were used to generate point mutation of the gene *crrB* (452 base C to T) in the ATCC13883 (Wang et al., 2018). For pSGKp-Spec-CrrB, the sgRNA targeting the *crrB* in the pSGKp-Spec was constructed using reverse PCR and the amplicon was transferred into *E. coli* DH5α. Then, the plasmid was extracted and confirmed by sanger sequencing and the confirmed plasmid was given the name pSGKp-Spec-CrrB. For pSGKp-Spec-500CrrB, the pSGKp-Spec-CrrB was digested by *Xba*I and *Bam*HI, and the ∼ 500 bp homologous arm up and downstream of the sequence S1 (ACG​CCG​GTG​ACT​ATC​TTA​CGT​GG) were cloned into the digested pSGKp-Spec-CrrB and the final plasmid was named as pSGKp-Spec-500CrrB. pSGKp-Spec-500CrrB was used to introduce the artificial N20 AAC​TTG​GTC​ACC​CGT​CCA​AT to replace the S1 (ACG​CCG​GTG​ACT​ATC​TTA​CG). For pSGKp-Spec-CT500, the sgRNA targeting the artificial N20 AAC​TTG​GTC​ACC​CGT​CCA​AT was constructed using inverse PCR and the amplicon was transferred into the *E. coli* DH5α. Then, the plasmid was extracted and confirmed by Sanger sequencing and then the plasmid was digested by *Xba*I and *Bam*HI, and the ∼ 500 bp homologous arm up and downstream of artificial N20 AAC​TTG​GTC​ACC​CGT​CCA​AT was cloned into the digested plasmid and the final plasmid was named pSGKp-Spec-CT500.

In order to introduce the artificial N20 AAC​TTG​GTC​ACC​CGT​CCA​AT into the ATCC13883 strain, 1 ml of an overnight culture of the pCasKP-harboring ATCC13883 strain from a fresh single colony was diluted into 100 ml of LB broth containing 30 μg/ml apramycin and incubated at 30°C. When the cell density reached an OD_600_ of approximately 0.2, 1 ml of 20% L-arabinose was added for induction of the lambda Red recombineering operon of pCasKP. After induction at 30°C for 2 h, the culture was prepared as electrocompetent cells. For electroporation, 50 μl of electrocompetent cells was thawed on ice for several minutes. Then, the cells were mixed with no more than 5 μl of pSGKp-Spec-500CrrB. The mixture was transferred into a 2-mm electroporation cuvette (Bio-Rad) and electroporated at 2.5 kV, 200 Ώ, and 25 μF. After being pulsed, the cells were recovered in 1 ml of antibiotic-free LB broth and incubated at 30°C for 2 h before being plated onto LB agar plates supplemented with the 30 μg/ml apramycin + 100 μg/ml spectinomycin. The plates were incubated at 30°C overnight. The colonies were confirmed by PCR, and the successfully introduced sequence was confirmed by the Sanger sequence. Finally, the strain for new rounds of point mutation, the pSGKp-Spec-500CrrB plasmid, was recycled by cultivation in the presence of sucrose.

In order to make point mutation, the pCasKP-harboring ATCC13883 strain with the artificial N20 into *crrB* was introduced to the pSGKp-Spec-CT500 by electroporation as described above, and the cells were recovered and plated onto LB agar plates supplemented with 30 μg/ml apramycin and 100 μg/ml spectinomycin as described above. Finally, the colonies were confirmed by PCR, and the successful point mutation was confirmed by Sanger sequencing. The primers used in this study are listed in Supplementary Table S2.

### Whole-genome sequencing

Genomic DNA was extracted using the Bacterial Genome Extraction Kit (TIANGEN, China). Genomic fragment libraries were constructed with the Paired-End Sample Preparation Kit (Illumina). Sequencing was performed on the Illumina HiSeq 2500 platform (Illumina, San Diego, CA, United States). Contig assembly was executed with the *de novo* SPAdes Genome Assembler (version 3.12.0) (Antipov et al., 2016). The mutations were identified using Snippy (https://github.com/tseemann/snippy).

### Protein expression and purification

The gene sequence coding truncated CrrB (135–353 amino acids, CrrB^T^) and its P151L mutant CrrB^T^P151L were synthesized using the PCR-based Accurate Synthesis (PAS) method. Restriction endonuclease recognition sites for *Nde*I and *Xho*I were introduced to insert the DNA into the expression vector pET-28a (+). The recombinant plasmid was transformed into *E. coli* TOP10 cells for cloning and plasmid preparation, and *E. coli* ArcticExpress cells for protein expression. The expression of the recombinant His-tagged CrrB^T^ and CrrB^T^P151L was induced by isopropyl-β-D-thiogalactoside (IPTG) at 16°C overnight. The proteins were found mainly in the supernatant. Affinity purification using the Ni-NTA column (Qiagen, Germany) was performed to purify the soluble CrrB^T^ and CrrB^T^_P151L_.

### ATP hydrolysis experiment

ATP hydrolytic activity of proteins was measured using Kinase-Glo^®^ Luminescent Kinase Assay (Promega, United States). A 50 µl reaction mixture (4 µg protein, 2 µM ATP, 40 mM Tris, pH 7.5, 20 mM MgCl_2_, 0.1 mg/ml BSA) with drugs (20 µg/ml 666-15 or 1.25 µg/ml PB alone or 1.25 µg/ml PB + 5, 10, 20 µg/ml 666-15) was incubated for 1 h at 37°C within a 96-well plate, and then mixed with 50 µl substrate reagent and incubated for 10 min at room temperature. The luminescence signal was recorded using an Infinite M200 PRO Multimode Microplate Reader (Tecan).

### Surface plasmon resonance experiment

A sensor chip CM5 (29-1049-88, Sweden) was used to immobilize CrrB^T^ protein by means of amino coupling. 666-15 was dissolved in the running buffer (50 mM hydroxyethyl piperazine ethyl sulfonic acid (HEPES), pH 7.5, 150 mM NaCl, 0.05% Tween 20, 5% DMSO). The channel with the sensor chip only was used as a blank, while the channel with the sensor chip and CrrB^T^ was used as an active control. A serial concentration of 666-15 (0.79, 1.56, 3.125, 6.25, 12.5, 25, 50 µM) was used to create curves. The affinity constant was calculated using Biacore T200 evaluation software (GE, United States).

### Quantitative reverse transcription-PCR analysis

*K. pneumoniae* ATCC13883 cells treated with PB (2 µg/ml) alone or a combination of 666-15 (50 µg/ml) and PB (2 µg/ml) were grown to the mid-log phase (OD_600_ = 0.8). Total RNA was extracted using the Bacterial Total RNA extraction kit (TIANGEN, Beijing, China). The purified RNA (2 µg) was used for reverse transcription to obtain cDNA with RT Master Mix (MCE, United States). SYBR Green qPCR Master Mix was used for Quantitative PCR (qPCR) on an Applied Biosystems real-time PCR system (ABI 7500). The 2^−ΔΔ*C*t^ method was used for determining the fold-changes of gene expression. The expression of 16S rRNA was applied as an internal control. The primers used in this study are listed in Supplementary Table S3.

### Membrane integrity assay

PBS (0.01 M, pH 7.4) was used for washing and resuspending *K. pneumoniae* ATCC13883 cells (OD_600_ = 0.5). PI (10 nM) was then incubated with PB (2 µg/ml) alone or with a combination of 666-15 (3.13, 6.25, 12.5 µg/ml) and PB (2 µg/ml). Fluorescence was measured (excitation and emission wavelengths were 535 and 615 nm, respectively) after incubation at 37°C for 30 min on an Infinite M200 PRO Multimode Microplate Reader (Tecan).

### Membrane polarization assay

HEPES (pH 7.0, 5 mM glucose) was used for washing and resuspending *K. pneumoniae* ATCC13883 (OD_600_ = 0.5) after PB (2 µg/ml) alone or the combination of 666-15 (25, 50, 100 µg/ml) and PB (2 µg/ml) treatment. DiSC_3_(5) (0.5 μM) was then added and incubated at 37 °C for 20 min. The membrane potential was measured (excitation and emission wavelengths were 622 and 670 nm, respectively) on an Infinite M200 PRO Multimode Microplate Reader (Tecan).

### Confocal laser scanning microscopy

Overnight cultured *K. pneumoniae* ATCC13883 cell cultures were washed three times and resuspended in saline (0.01 M). After 1 h incubation with PB (2 µg/ml) alone or the combination of 666-15 (50 µg/ml) and PB (2 µg/ml), the bacteria were harvested and then washed and resuspended in saline. A total volume of 1 ml with SYTO_9_ (1.67 mM, 1.5 μl) and PI (10 mM, 1.5 μl) was incubated for sample staining (room temperature in the dark for 15 min). Fluorescence images were obtained by using a confocal laser scanning microscope (Leica TCS SP8).

### Scanning electron microscope

*K. pneumoniae* ATCC13883 cell cultures at the mid-log phase were washed and resuspended in 0.01 M saline (OD_600_ = 0.5). After 1 h incubation with PB (2 µg/ml) or 666-15 (50 µg/ml) alone or the combination of 666-15 (50 µg/ml) and PB (2 µg/ml), bacteria were fixed in 2.5% glutaraldehyde, dehydrated, and dried by critical point drying in CO_2_. The dried sample was covered with a conductive gold film and the images were captured at 3 kV accelerating voltage using a JEOL JSM-7900F SEM in the secondary electron mode.

### Proteome analysis

Proteome analysis of *K. pneumoniae* ATCC13883 cells treated with PB (2 µg/ml) alone or the combination of 666-15 (50 µg/ml) and PB (2 µg/ml) was performed according to our previous report (Zhang et al., 2020). Briefly, a 100 ml culture (OD_600_ = 0.8) was centrifuged (12,000 rpm, 3 min) and washed with precooled PBS. A high-intensity ultrasonic processor (Scientz) was used for sample sonication (three times on ice) in lysis buffer (8 M urea, 1% protease inhibitor cocktail). After centrifugation (12,000 rpm, 4°C, 10 min), the supernatant was collected for further study. The protein concentration was determined using the Bradford method. An aliquot of 50 µg of extracted proteins was incubated with dithiothreitol (DTT) (200 mM) (37°C, 1 h), and then diluted eight times with 50 mM ammonium bicarbonate (ABC) buffer. Trypsin (trypsin/protein = 1:25) was then added and incubated at 37°C overnight.

The digestion was terminated by adding 50 μl of 0.1% formic acid (FA). The C18 columns were washed using 100 μl of 100% acetonitrile (ACN) and then centrifuged at 1,200 rpm for 3 min. The columns were washed again with 100 μl of 0.1% FA and then centrifuged at 1,200 rpm for 3 min. The samples (≤30 μg) were added to the column and then centrifuged at 1,200rpm for 3 min. The columns were washed twice with 100 μl of 0.1% FA and centrifuged at 1,200 rpm for 3 min. Gradients of ACN (6%, 9%, 12%, 15%, 18%, 21%, 25%, 30%, 35%, and 50%) were used for elution. Thermo Orbitrap Fusion mass spectrometry was used for label-free mass spectrometry. The scan events were set as a full MS scan of 250–1450 *m/z* at a mass resolution of 120,000. The normalized collision energy was set to 30% and an activation time of 50 ms. Linear ion trap fast mode was used for the second stage with automatic gain control (AGC) of 7,000, maximum injection time of 35 ms, and dynamic exclusion time of 18 s. The Swiss-Prot and UniProt databases were used in this experiment. The resulting MS/MS data were processed using Maxquant 1.5.2.8. The search parameters were as follows: precursor ion mass tolerance, ± 15 ppm; fragment ion mass tolerance, ± 0.5 Da; max missed cleavages, 2; static modification, carbox yamidomethylation (57.021 Da) of Cys residues; dynamic modifications, oxidation modification (+15.995 Da) of Met residues. *P* value (Student’s *t*-test with a Benjamini–Hochberg false discovery rate adjustment) < 0.05 and a fold change >1.2 were considered significant.

The GO (Gene Ontology) and KEGG (Kyoto Encyclopedia of Genes and Genomes) annotations were taken from the UniProt database. Enrichment analysis was performed using the hypergeometric distribution with the following formula:

$$p-value=\sum_{i=m}^{M}\frac{\left(\begin{array}{l} M\\ i \end{array}\right)\left(\begin{array}{l} N-M\\ n-i \end{array}\right)}{\left(\begin{array}{l} N\\ n \end{array}\right)}=1-\sum_{i=0}^{m-1}\frac{\left(\begin{array}{l} M\\ i \end{array}\right)\left(\begin{array}{l} N-M\\ n-i \end{array}\right)}{\left(\begin{array}{l} N\\ n \end{array}\right)}.$$

where *N* is the total number of background annotated proteins; *M* is the number of background proteins with a special annotation term; *n* is the number of analyzed proteins; and *m* is the number of analyzed proteins with a special annotation term.

The degree of enrichment was calculated with the formula -log10 (*p value*). The enrichment results were visualized using the bubble diagram, in which the ratio (k/M), enrichment degree, and the number of proteins (*m*) for each enriched term were displayed.

### Lipopolysaccharide extraction and quantitative analysis

*K. pneumoniae* ATCC13883 cells were grown until an OD_600_ of 0.2, and then treated with PB (2 µg/ml) alone or the combination of 666-15 (10, 30, 50, 70 µg/ml) and PB (2 µg/ml). Bacteria were centrifuged (12,000 rpm for 10 min) when the OD_600_ was 0.6. The precooled PBS was used to wash and resuspend bacterial cells. After the addition of sodium dodecyl sulfate (SDS) lysis buffer and centrifugation (12,000 rpm for 10 min at 4°C), the supernatant was harvested for LPS quantification using an ELISA kit (Cloud-Clone, Wuhan, China) according to the manufacturer’s instructions.

### Lipid A extraction and structural analysis

The cell cultures of *K. pneumoniae* ATCC13883 (OD_600_ = 0.6) were 1:100 diluted and treated with PB (2 µg/ml) alone or the combination of 666-15 (50 µg/ml) and PB (2 µg/ml) in 100 ml of fresh LB medium until an OD_600_ of 0.6. PBS was used to wash (20 ml × 3) and resuspend (4 ml) the bacterial cells. Chloroform (5 ml) and methanol (10 ml) were added to the samples and turned and mixed, and then incubated for 20 min at room temperature. After centrifugation (3,500 rpm for 15 min), we used 20 ml of single-phase Bligh-Dyer mixture (chloroform/methanol/PBS, pH 7.4; 1:2:0.8 v/v) to wash the precipitate. Then, 5.4 ml of mild acid hydrolysis buffer (50 mM sodium acetate, pH 4.5; 1% SDS) was used to treat the precipitate and suspend using an ultrasonic disintegrator. The sample was then boiled for 45 min and cooled to room temperature. After the addition of chloroform (6 ml) and methanol (6 ml), the lower phase was extracted by centrifugation (2,500 rpm for 10 min). The lower phase of the pre-equilibrated two-phase Bligh-Dyer mixture (chloroform/methanol/water; 2:2:1.8 v/v) was then added and mixed. The lower phase of the solution was harvested by centrifugation (2,500 rpm for 10 min). The upper phase of the pre-equilibrated two-phase Bligh-Dyer mixture (22.8 ml) was added to the sample and mixed. After centrifugation (2,500 rpm for 10 min), the lower phase was harvested and dried. Structural analysis of lipid A was performed in a negative mode on a Q-Exactive Hybrid Quadrupole-Orbitrap Mass Spectrometer (Thermo Fisher) (Henderson et al., 2013; Aye et al., 2020).

### Mouse infection model

A lethal pneumonia mice model was used for evaluating the *in vivo* activity of 666-15. Female BALB/c mice 6-8 weeks old (20 ± 2 g) and specific-pathogen-free were purchased from Hunan SJA Laboratory Animal Limited Company (Changsha, China). The inoculation of 1.0×10^8^ colony-forming units (CFUs) of clinical PB-resistant *K. pneumoniae* (P2418-1, the whole-genome sequencing data was deposited in GenBank under BioProject PRJNA517992) using noninvasive intratracheal instillation under direct vision was performed after intraperitoneally anesthetized with pentobarbital sodium (75 mg/kg). PB and 666-15 were administered by intraperitoneal injection alone at doses of 0.2 and 10 mg/kg, respectively. For the combination treatment, we used 1, 5, or 10 mg/kg 666-15 in combination with 0.2 mg/kg PB.

The survival of the mice was observed after 3 and 5 days of infection. All the animal experiments in this study were performed in compliance with the Regulations for the Administration of Affairs Concerning Experimental Animals approved by the State Council of the People’s Republic of China. All animal experiments in this study were approved by the Animal Ethical and Experimental Committee of the Army Military Medical University (approval No. 2011-04) based on the National Institutes of Health guide for the care and use of laboratory animals.

### Statistical analysis

Statistical analysis was performed using GraphPad Prism software version 8.0 (La Jolla, California). A value of *p* < 0.05 is considered to be statistically significant.

## Results

### 666-15 enhances the antibacterial activity of polymyxin B

**FIGURE 1:**
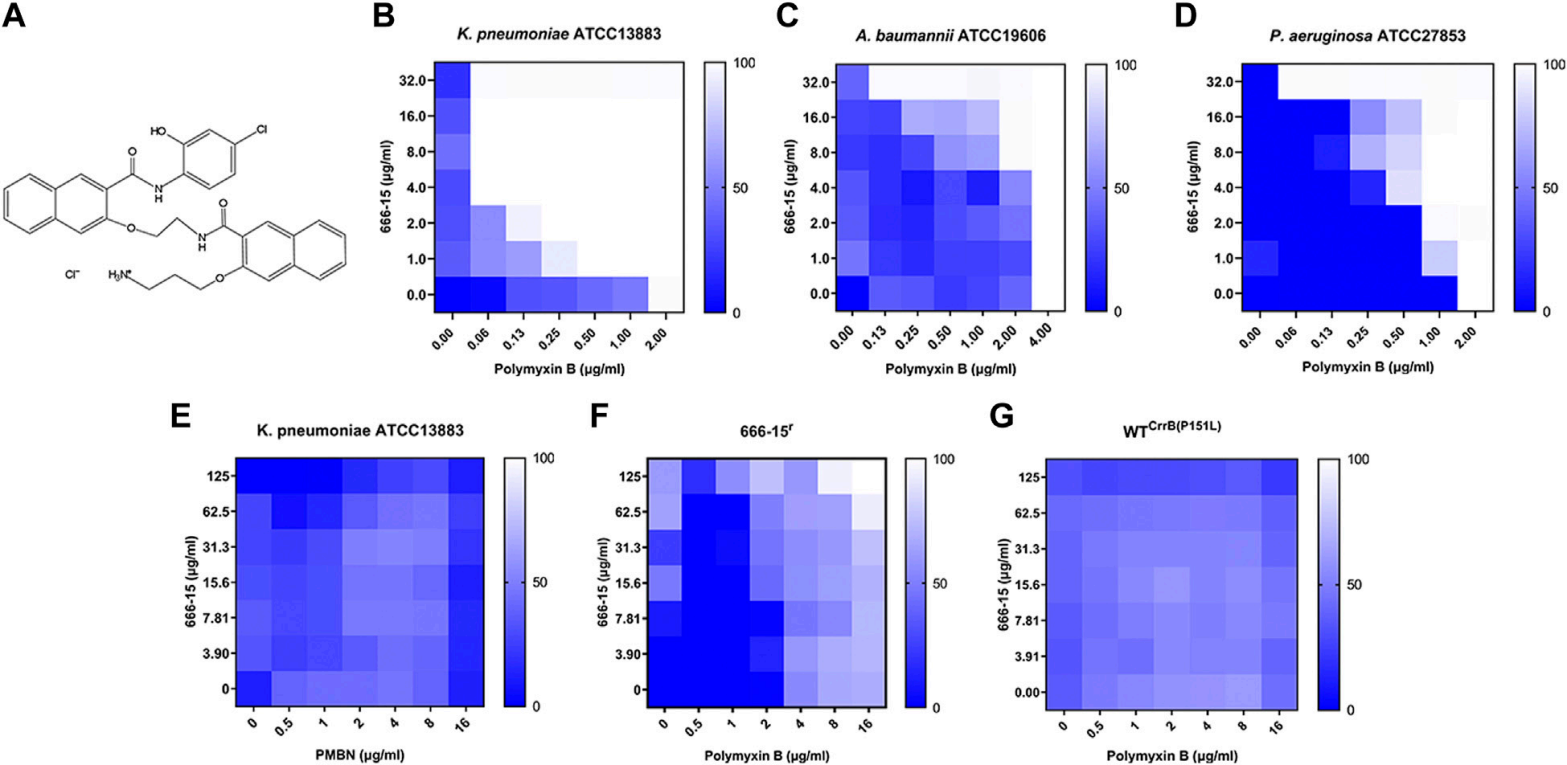
**(A)** Chemical structure of compound 666-15. 666-15 enhances the activity of polymyxin B (PB) against **(B)** *Klebsiella pneumoniae* ATCC13883, **(C)** *Acinetobacter baumannii* ATCC19606, and **(D)** *Pseudomonas aeruginosa* ATCC27853. **(E)** Chequerboard experiment of 666-15 and polymyxin B nonapeptide (PBMN) against *Klebsiella pneumoniae* ATCC13883. The chequerboard experiment of 666-15 and PB against the **(F)** spontaneous mutant 666-15^r^ and **(G)** artificial mutant WT ^CrrB(P151L)^.

We first used the FICI experiment to screen PB synergists from the Bioactive Compound Library against *K. pneumoniae* ATCC13883. Of these compounds, 666-15 showed a synergistic effect on PB (FICI ≤0.281) (Figure 1A). The synergistic effects were also found in *Acinetobacter baumannii* ATCC19606 (FICI ≤0.281) and *Pseudomonas aeruginosa* ATCC27853 (FICI ≤0.281), while 666-15 itself had no antibacterial activity (MIC >100 µg/ml) (Figures 1B–D). No synergistic effect was found between 666-15 and polymyxin B nonapeptide (PBMN) against *K. pneumoniae* ATCC13883, suggesting that the increase in membrane permeability induced by PBMN had no effect on the activity of 666-15 (Figure 1E). This result also indicated that the synergistic effect between 666-15 and PB was due to the enhancement of the antibacterial activity of PB by 666-15. In this case, 666-15 should be considered an antibiotic adjuvant (Wright, 2016). No synergistic effects were observed between 666-15 and other antibiotics (Supplementary Table S4).

Amino acid substitutions in CrrB decrease the enhancement activity of compound 666-15.

To elucidate the mode of action of 666-15, we first successfully obtained a strain that was resistant to the enhancement effect of 666-15 (666-15^r^) through serial passaging. Meanwhile, a shift in the MIC values of PB (2 to >16 µg/ml) was also observed in 666-15^r^ and WT^CrrB(P151L)^ (Figures 1F,G). Whole-genome sequencing combined with genome comparative analysis revealed that 666-15^r^ harbored a mutation from cytosine to thymine at position 452 of *crrB* (encoding CrrB carrying a proline-to-leucine substitution at position 151, P151L) compared to the parental strain *K. pneumoniae* ATCC13883 (Table 1).

**TABLE 1:** Mutation identified in a strain (666-15^r^) that was resistant to the enhancement of 666-15 through serial passaging.

Locus_Tag	Gene	Codon position	Amino acid position	Product
FIU12_20585	*crrB*	452C>T	Pro151Leu	Sensor protein CrrB

CrrB is a protein that can positively regulate PB resistance (McConville et al., 2020). It is reasonable that 666-15 enhances the activity of PB by directly acting on CrrB. However, it has been reported that PB could also induce the mutation of *crrB* (Sun et al., 2020). As 666-15^r^ was isolated under exposure to the combination of PB and 666-15, another possibility was that the mutation of CrrB was a response to PB.

### Effects of 666-15 on the activity of CrrB

To verify the interaction between 666-15 and CrrB, we first produced recombinant truncated CrrB (CrrB^T^) protein with HisKA (histidine kinase) and HATPase (histidine kinase ATPase) domains and its mutant (CrrB^T^P151L). Interestingly, when we harvested the purified recombinant protein, we found that CrrB^T^P151L mainly formed a dimer, while CrrB^T^ had both dimer and monomer forms (Supplementary Figure S1).

**FIGURE 2:**
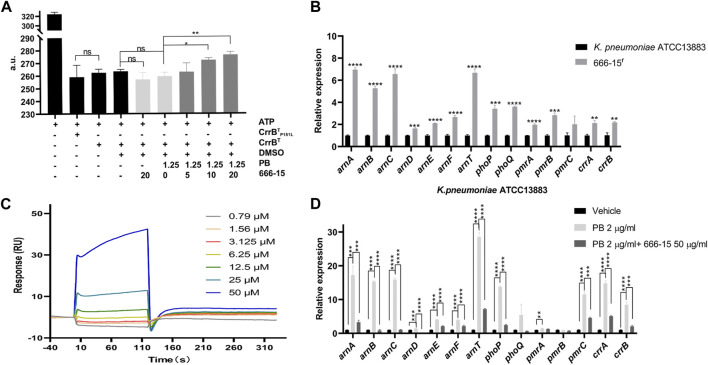
Interactions between 666-15 and CrrB^T^. **(A)** Effects of 666-15 on the ATP hydrolyzation activity of recombinant proteins. **(B)** Comparison of transcription levels of genes regulated by the CrrA/B two-component system between 666-15^r^ that was resistant to the enhancement of 666-15 and *K. pneumoniae* ATCC13883. **(C)** Surface plasmon resonance analysis of the interaction between 666-15 and CrrB^T^. *K*_*D*_ values were determined from the ratio between the kinetic rate constants (*k*_a_ */k*_d_). **(D)** Effects of 666-15 on the transcription levels of genes regulated by the CrrA/B two-component system in the presence of PB. For **(A,B,D)**, the mean of three biological replicates are shown and error bars represent the standard deviation (SD) (*n* = 3); *p < 0.05; **p < 0.01; ***p < 0.001; ****p < 0.0001. *P* values were determined using an unpaired *t*-test between two groups or non-parametric one-way ANOVA among multiple groups.

Previous studies have shown that CrrB, as a member of the CrrA/B two-component system, plays a role in phosphorylating CrrA and inducing the expression of the *phoP/Q*, *pmrA/B,* and *arnBCADTEF* operons (Cheng et al., 2016; Jayol et al., 2017; Jeannot et al., 2017; Cheng et al., 2018). To confirm the activity of the recombinant proteins, we first identified the ability of CrrB^T^ and CrrB^T^P151L proteins to hydrolyze ATP (Figure 2A). Surprisingly, no difference was found in the abilities of CrrB^T^ and CrrB^T^P151L to hydrolyze ATP. The transcription levels of *arnA-F*, *arnT*, *phoP*, *phoQ*, *pmrA*, *pmrB, crrA,* and *crrB* were significantly increased in 666-15^r^ compared with those in *K. pneumoniae* ATCC13883 (Figure 2B). This finding may explain why the mutation in CrrB led to PB resistance.

To identify the interaction between 666-15 and CrrB^T^, we performed SPR experiments and found moderate affinity, with an association rate constant (*k*_*a*_) of 65.5 M^−1^s^−1^, dissociation rate constant (*k*_*d*_) of 0.726 s^−1,^ and equilibrium dissociation constant (*K*_*D*_) of 1.79 × 10^-5^ M (Figure 2C). These results indicated binding of 666-15 and CrrB^T^ and suggested that 666-15 might affect the CrrB^T^ function. No binding of 666-15 and CrrB^T^P151L was observed. However, the effect of 666-15 on the ATP hydrolyzation activity of CrrB^T^ could be observed only in the combination with PB, and no difference was observed when the compound was used alone (Figure 2A). We also found that compared to PB treatment alone, the combination treatment of 666-15 with PB could significantly decrease the transcription levels of *arnA-F*, *arnT*, *phoP, pmrC, crrA,* and *crrB* in *K. pneumoniae* ATCC13883 (Figure 2D). These results indicated that 666-15 treatment reduced the regulation of the *arnBCADTEF* operon by affecting CrrB activity in the presence of PB.

### Proteomic changes in *K. pneumoniae* ATCC13883 in response to 666-15

**FIGURE 3:**
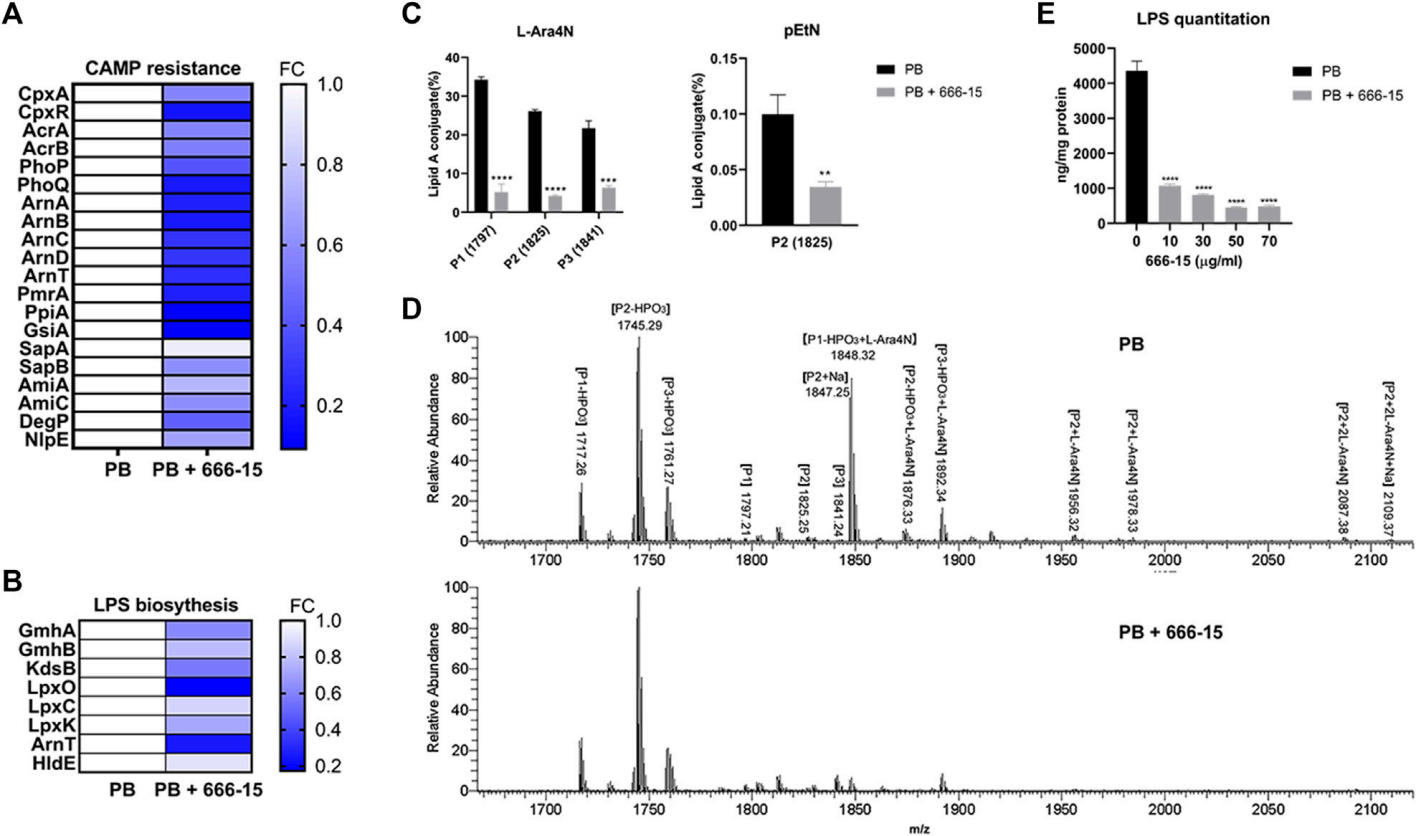
Effects of 666-15 on lipid A modification and lipopolysaccharide (LPS) biosynthesis. **(A)** Differentially expressed proteins (DEPs) involved in cationic antimicrobial peptide (CAMP) resistance. **(B)** Differentially expressed proteins (DEPs) involved in LPS biosynthesis. **(C)** The effect of 666-15 on 4-amino-4-deoxy-L-arabinose (L-Ara4N) and phosphoethanolamine (pEtN) modification of lipid A in the presence of PB. **(D)** Mass spectra of lipid A from *K. pneumoniae* ATCC13883 with PB or the combination of PB and 666-15 treatment. P1, P2, and P3 represent *m/z* of 1797, 1825, and 1841, respectively. The structures are shown in Supplementary Table S7. **(E)** The effect of 666-15 on LPS biosynthesis. For **(A,B)**, a *p*-value (Student’s *t*-test with a Benjamini–Hochberg false discovery rate adjustment) < 0.05 and a fold change >1.2 were considered significant; For **(C,E)**, the mean of three biological replicates are shown and error bars represent the standard deviation (SD) (*n* = 3); *p < 0.05; **p < 0.01; ***p < 0.001; ****p < 0.0001. *P* values were determined using an unpaired *t*-test between two groups or non-parametric one-way ANOVA among multiple groups.

We profiled the proteomic changes in *K. pneumoniae* ATCC13883 treated with PB (2 µg/ml) alone or the combination of 666-15 (50 µg/ml) with PB (2 µg/ml). Differentially expressed proteins (DEPs) involved in the cationic antimicrobial peptide (CAMP) resistance are shown in Figure 3A. Compared with that in the PB treatment group, the expression of ArnABCDT, PhoP/Q, and PmrA proteins regulated by CrrA/B was significantly decreased after treatment with the PB and 666-15 combination. This result is consistent with the effects of 666-15 on transcription levels (Figure 2D). Moreover, we also found changes in the levels of other proteins involved in the CAMP resistance pathway, such as CpxA, CpxR, AcrA/B, and SapA/B (Figure 3A). The expression of proteins in the LPS synthesis pathway related to PB resistance was also affected by 666-15 in the presence of PB (Figure 3B). The changes in the levels of other DEPs are shown in Supplementary Table S5.

### Effects of 666-15 on lipid modification and lipopolysaccharide synthesis

The changes in proteomic and transcriptional levels showed that the lipid A modification pathway was inhibited by 666-15 in the presence of PB. To investigate whether this inhibition could eventually prevent lipid A modification, we used LC–MS/MS to measure the proportion of modified lipid A in *K. pneumoniae* ATCC13883 treated with PB (2 µg/ml) alone or the combination of 666-15 (50 µg/ml) with PB (2 µg/ml).

Compared with PB alone, the combination of PB and 666-15 significantly reduced the proportion of lipid A species with L-Ara4N moieties (based on *m/z* 1797, 1825, 1841) (Figures 3C,D). A decreased proportion of lipid A species with a pEtN moiety (based on *m/z* 1825) was also observed (Figure 3C). All the lipid A structures presented in this study are shown in Supplementary Tables S6 and S7. In addition, compared with PB alone, the combination of PB and 666-15 reduced the content of bacterial LPS (Figure 3E).

### Effects of 666-15 on membrane integrity and potential

**FIGURE 4:**
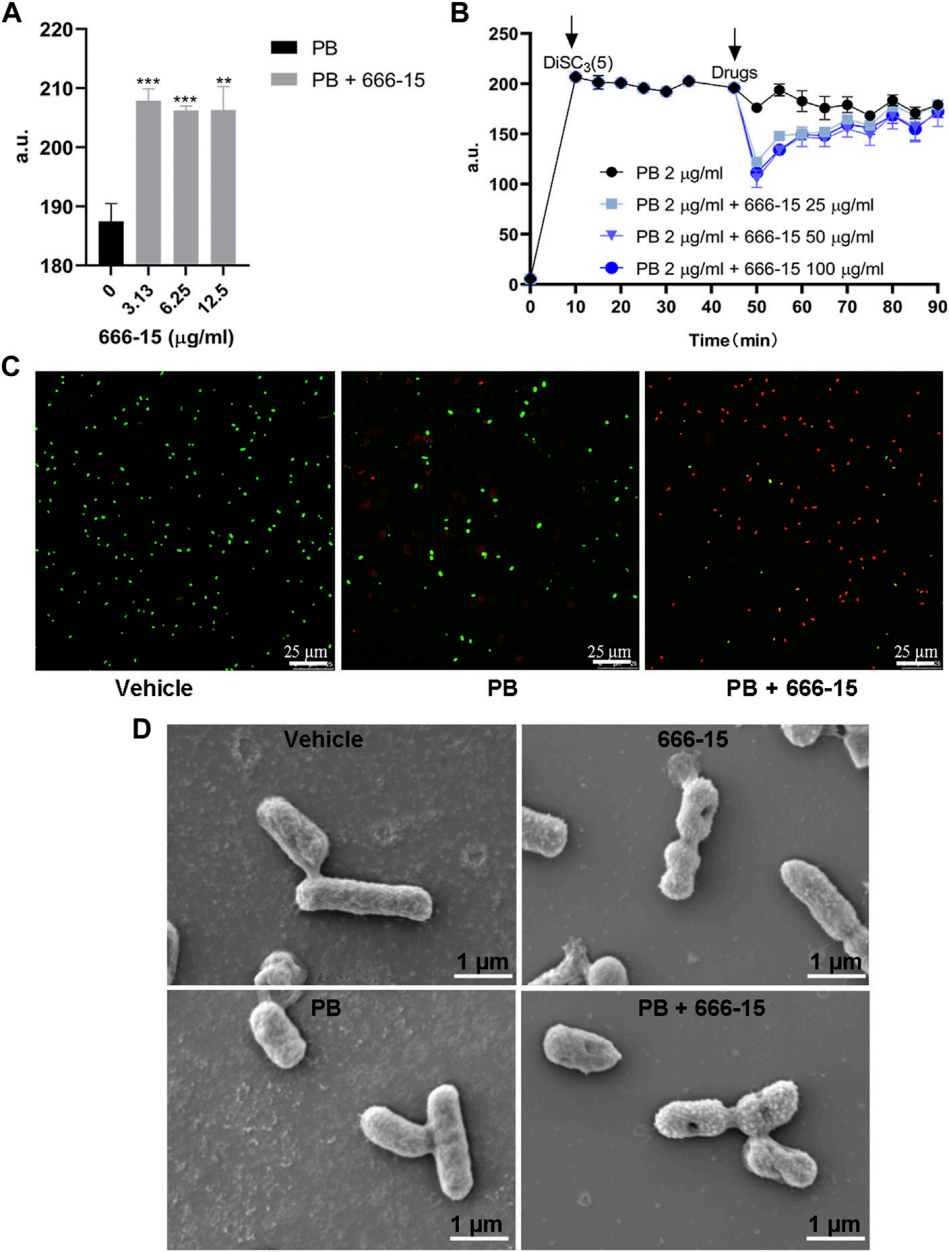
The effects of 666-15 on the permeability of the **(A)** Inner membrane probed with propidium iodide (PI) and **(B)** membrane potential of the inner membrane probed with 3,3-dipropylthiadicarbocyanine iodide DiSC_3_(5). a.u., arbitrary unit. **(C)** Confocal images of *K. pneumoniae* ATCC13883 under the treatment of PB alone or the combination of PB and 666-15 for 1 h. Intracellular nucleic acid was stained with PI (red), representing dead cells, while the membrane was stained with SYTO9 (green), representing viable cells (bar: 25 µm). **(D)** Scanning electron microscopy images of *K. pneumoniae* treated with PB (2 µg/ml) or 666-15 (50 µg/ml) monotherapy and in combination (bar: 1 µm). For **(A,B)**, the mean of three biological replicates are shown and error bars represent the standard deviation (SD) (*n* = 3); **p < 0.01; ***p < 0.001. *P* values were determined using non-parametric one-way ANOVA.

Intrinsic mechanisms, including permeability barriers such as the cell membrane in Gram-negative bacteria, are potential targets for antibiotic adjuvants. To identify whether 666-15 affects this mechanism, we first tested the effects of 666-15 on membrane integrity. Our results showed that the combination of 666-15 and PB resulted in increased cell membrane disruption compared with that obtained with PB alone (Figure 4A). The potential of the membrane was not affected by 666-15 (Figure 4B). Confocal laser microscopy showed that 666-15 resulted in increased intracellular nucleic acid (red) staining and decreased membrane (green) staining (Figure 4C).

After confirming the effects of 666-15 on membrane integrity and potential, SEM was conducted to examine the effect of PB and 666-15 monotherapy and in combination on the membrane structure of *K. pneumoniae* at 1 h (Figure 4D). Compared with the untreated group, no difference in the bacterial cell membrane under PB monotherapy was found. Under 666-15 monotherapy, some bacteria have a depression in the center of the cell membrane and a rough surface. These phenomena can also be observed more clearly under combination therapy.

### The activity of 666-15 *in vivo*

**FIGURE 5:**
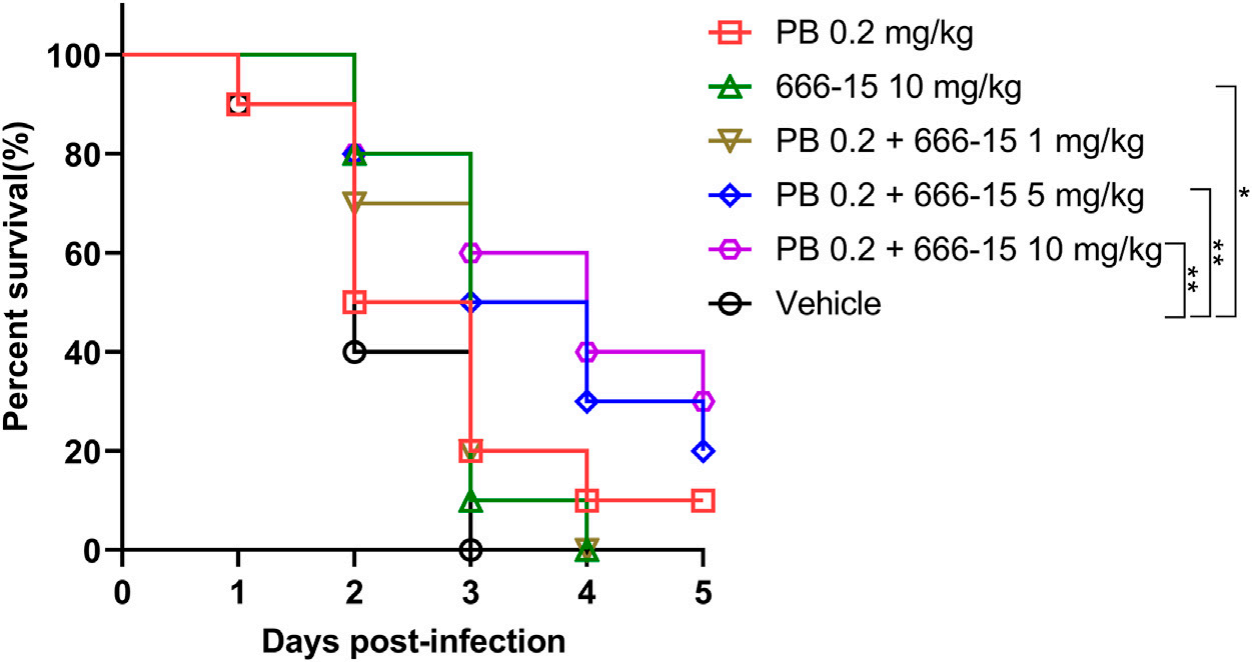
Activity of 666-15 *in vivo*. Infected female mice with PB-resistant *K. pneumoniae* (1.0 × 10^8^ CFU) using intratracheal instillation. Intraperitoneal injection was used for the treatment of 666-15 or PB alone and in the combination of 666-15 with PB (*n* = 10 for each group). *P* values were determined using the two-sided, log [rank] (Mantel–Cox) test.

A clinical PB-resistant *K. pneumoniae* strain P2418-1 (MIC = 8 μg/ml) was used to evaluate the activity of 666-15 *in vivo*. Three days after infection, all mice in the solvent group were dead, in contrast to 10% and 20% survival rates in the treatment groups with 666-15 (10 mg/kg) or PB (0.2 mg/kg) alone, respectively, and 20%, 50% and 60% survival rates in the treatment groups with combination therapy (0.2 mg/kg PB + 1, 5 or 10 mg/kg 666-15, respectively) (Figure 5). Five days after infection, all mice in the treatment groups with 666-15 (10 mg/kg) alone or in combination (0.2 mg/kg PB + 1 mg/kg 666-15) were dead, in contrast to 10%, 20% and 30% survival rates in the treatment groups with PB (0.2 mg/kg) alone or in combination (0.2 mg/kg PB + 5 or 10 mg/kg 666-15).

## Discussion

In *Enterobacteriaceae* (*Klebsiella, Escherichia, Enterobacter,* and *Salmonella)*, the majority of PB resistance mechanisms involve the addition of cationic groups (L-Ara4N, pEtN, and/or galactosamine) to LPS. The LPS modification-associated genes code for enzymes (PmrC, PmrE, and ArnBCADTEF) that directly mediate LPS modifications; the two-component systems (PmrA/B and PhoP/Q) that regulate these enzymes; and the regulators (MgrB and CrrA/B) of these two-component systems (Poirel et al., 2017; Moffatt et al., 2019). Therefore, all of the key factors mentioned above related to LPS modification are targets for the development of PB enhancers.

In recent years, the incidence of colistin-resistant *K. pneumoniae* infections has increased rapidly, while the emergence of colistin-resistant hypervirulent *K. pneumoniae* poses a severe challenge to public health (Wang et al., 2017; Mobasseri et al., 2019; Liu et al., 2022). Herein, we described the discovery of the small molecule 666-15 that enhances the activity of PB against *K. pneumoniae*. Through the isolation of resistant strains and whole-genome sequencing, we found that 666-15 had no effect on the activity of PB against 666-15^r^ carrying the CrrB P151L mutation. Previous studies have shown that amino acid substitution at position 151 of CrrB from proline to serine (P151S) was responsible for PB resistance via the upregulation of *pmrA/B* and activation of the *arnBCADTEF* operon (Cheng et al., 2016; Jayol et al., 2017; Jeannot et al., 2017). In this study, compared to that against *K. pneumonia* ATCC13883, we found that the MIC of PB against 666-15^r^ was also increased. The change in transcription in 666-15^r^ revealed that the P151L mutation also resulted in the upregulation of expression of *pmrA/B* and the *arnBCADTEF* operon, which is consistent with previous reports about P151S amino acid substitutions (Cheng et al., 2016; McConville et al., 2020).

The structural basis of the signal transduction in the two-component system (TCS) has been well elucidated (Yamada and Shiro, 2008). A sensor histidine kinase (HK) and a response regulator (RR) form the typical TCS system of bacteria. The HisKA domain of the HK consists of two subdomains, a dimerization, and a catalytic domain, which are responsible for dimer formation, ATP hydrolysis, and autophosphorylation, thus facilitating the subsequent transfer of the phosphoryl group to the RR. It is important to note that the function of hydrolyzed ATP is achieved in the dimer form (Yamada and Shiro, 2008). For the CrrA/B TCS, CrrB acts as an HK and CrrA as a RR. In this study, we observed the difference in the dimer ratio between CrrB^T^ and CrrB^T^P151L and transcription level changes of the downstream targets of the CrrA/B TCS between *K. pneumoniae* ATCC13883 and 666-15^r^, leading to the conclusion that the point mutation of P151L resulted in the enhancement of the CrrA/B TCS function, thus reasonably explaining the mechanism of PB resistance.

Given that the catalytic domain of HisKA is responsible for phosphorylating the special His residue in the dimerization domain by using ATP, we can evaluate the effect of 666-15 on the CrrB function by detecting ATP consumption. However, there was no difference in ATP hydrolysis activity between CrrB^T^ and CrrB^T^P151L, indicating that P151L did not improve the ability of CrrB to utilize ATP. Therefore, we speculated that P151L might play a role by affecting downstream signal transduction. This problem was not clarified in this study, which was a limitation. Moreover, the effect of 666-15 on the consumption of ATP by CrrB could be observed only in combination with PB, which may be due to the low activity of CrrB in the absence of external stimuli. The SPR result confirmed the direct interaction between 666-15 and CrrB^T^. These results suggest that 666-15 plays an inhibitory role through directly binding CrrB and that bacteria can resist this inhibition through P151L mutation. Regarding how this mutation causes loss of 666-15 function, this study failed to clarify this detail. The mechanism may occur through a structural change that CrrB fails to bind or through enhancement of its own function (not ATP hydrolysis), which is worthy of further study and also a limitation of this study. Proteome and lipid A structural analysis confirmed that 666-15 leads to changes in the protein levels of the CrrA/B-regulated target, and the corresponding lipid A modification level is also inhibited. However, we also observed the effect of 666-15 on the content of bacterial LPS. Since some organisms become more resistant as LPS levels decrease, we believe that it would impact PB resistance (Moffatt et al., 2010). Therefore, the effect of 666-15 on bacterial PB susceptibility should be double-sided but it is ultimately shown to be more susceptible. The efficacy and previously confirmed safety of 666-15 *in vivo* demonstrated its potential in clinical application and support the further development of a PB synergist based on this compound (Li et al., 2016).

In conclusion, we discovered that a small compound enhances the activity of PB against *K. pneumoniae* by inhibiting the CrrA/B TCS. As far as we know, this is the first inhibitor that targets CrrB. Our study opens a new way to develop PB synergists by targeting the factors that regulate lipid A modification.

## Data Availability

The datasets presented in this study can be found in online repositories. The names of the repository/repositories and accession number(s) can be found in the article/Supplementary Material.

## Glossary

ACN: acetonitrile
AGC: automatic gain control
CAMP: cationic antimicrobial peptide
DEPs: differentially expressed proteins
CFUs: colony-forming units.
PB: polymyxin
BCREB: cyclic adenosine monophosphate response element-binding protein
MDR: multidrug-resistant
PR: polymyxin resistance
PK-PD: Pharmacokinetic-pharmacodynamic
LPS: lipopolysaccharide
pEtN: phosphoethanolamine
L-Ara4N: 4-amino-L-arabinose
MTT: 3-(4,5)-dimethylthiazol(-z-y1)-2,5-di-phenytetrazoliumromide
PBS: phosphate-buffered saline
PI: propidium iodide
DiSC3(5): 3,3-Dipropylthiadi-carbocyanine iodide
MIC: minimal inhibitory concentration
FICI: fractional inhibitory concentration index
PAS: PCR-based Accurate Synthesis
qRT–PCR: quantitative reverse transcription polymerase chain reaction
HEPES: hydroxyethyl piperazine ethyl sulfonic acid
SEM: Scanning electron microscope
TCS: two-component system;
HK: histidine kinase
RR: response regulator
DTT: dithiothreitol
ABC: ammonium bicarbonate
FA: formic acid
SDS: sodium dodecyl sulfate
PBMN: polymyxin B nonapeptide
