# Supplementary material for: A cyclic adenosine monophosphate response element-binding protein inhibitor enhances the antibacterial activity of polymyxin B by inhibiting the ATP hydrolyzation activity of CrrB
Source: Front Pharmacol. 2022 Sep 6;13:949869. doi: 10.3389/fphar.2022.949869 (PMC9485624; doi:10.3389/fphar.2022.949869)
Supplement: Supplementary file 1 [file DataSheet1.ZIP › supplementary material-1.docx]

# Supplementary Figures and Tables

# 1.1 Supplementary Figures

**
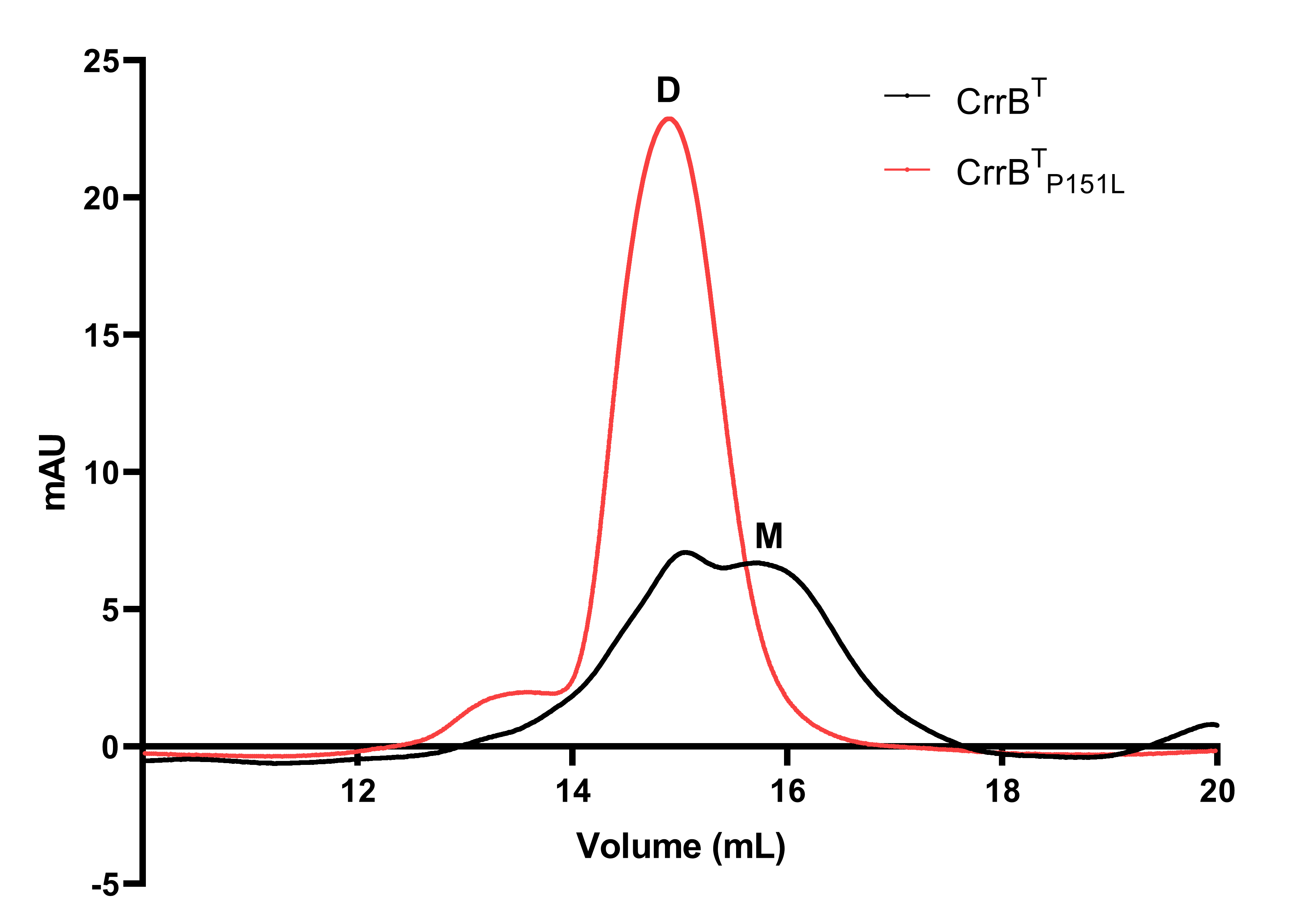
**

**Supplementary Figure 1.** Absorption peaks of truncated CrrB (135-353 amino acids, CrrB^T^) and its P151L mutant (CrrB^T^_P151L_) at 280 nm by using molecular sieve. The dimer protein (D, molecular weight 51 kDa) was located at 15 ml and the monomer (M, molecular weight 25.5 kDa) at 16 ml.

# 1.2 Supplementary Tables

**Supplementary Table 1.** The detailed information of the strains.

| **Strain** | **Species** | **Source** |
| --- | --- | --- |
| **ATCC13883** | *Klebsiella pneumoniae* | American Type Culture Collection |
| **ATCC27853** | *Pseudomonas aeruginosa* | American Type Culture Collection |
| **ATCC19606** | *Acinetobacter baumannii* | American Type Culture Collection |
| **P2418-1** | PB-resistant *Klebsiella pneumoniae* | Shenzhen People’s Hospital |
| **666-15^r^** | spontaneous mutant of ATCC13883 | This work |
| **WT ^CrrB(P151L)^** | artificial mutant of ATCC13883 | This work |

**Supplementary Table 2.** Primers used in CRISPR-Cas9 gene editing.

| **Primers** | **Sequence** |
| --- | --- |
| **CrrB-TF** | CGGAAAAAGCTTGAACATGCC |
| **CrrB-TR** | CAAAACTCGGCAGTATGTGGG |
| **CrrB-HF1** | CGAATTCCTGCAGCCCGGGGGATCCATGAACAAAGAAACGATTCTGAGCC |
| **CrrB-HR1** | ATTGGACGGGTGACCAAGTTCCGGAGTTCATGAGCAATTGC |
| **CrrB-HF2** | AACTTGGTCACCCGTCCAATTGGGAGGCTTCAGGGACTGGTTG |
| **CrrB-HR2** | CCACCGCGGTGGCGGCCGCTCTAGACAGGCCATGAGAGTTTGAAAAGT |
| **crrB-N20F** | ACGCCGGTGACTATCTTACG |
| **Art-CrrB-N20F** | AACTTGGTCACCCGTCCAAT |
| **Infu-crrB-N20F** | ACGCCGGTGACTATCTTACGGTTTTAGAGCTAGAAATAGCAAGTTAAAATAAGGC |
| **Infu-crrB-N20R** | CGTAAGATAGTCACCGGCGTACTAGTATTATACCTAGGACTGAGCTAGC |
| **pSGKP-F1** | TGCAGACTACGGGCCTAAAG |
| **pSGKP-R1** | AGCGCAACGCAATTAATGTG |
| **CT-CrrB-HF2** | ACGCTGGTGACTATCTTACGTGGGAGGCTTCAGGGACTGGTTG |
| **CT-CrrB-HR1** | CGTAAGATAGTCACCAGCGTCCGGAGTTCATGAGCAATTGC |
| **Infu-CTcrrB-N20F** | ACTTGGTCACCCGTCCAAATGTTTTAGAGCTAGAAATAGCAAGTTAAAATAAGGC |

**Supplementary Table 3.** Primers used in qRT-PCR.

| **Gene** | **Forward primers** | **Reverse primers** |
| --- | --- | --- |
| ***16SrRNA*** | AGGCCTAACACATGCAAGTC | TGCAATATTCCCCACTGCTG |
| ***arnA*** | TAACCCGGAAAACGAAGCGA | CGGTCTCTTCCATCTCGACG |
| ***arnB*** | GGACGCTTATGATCGCCAGA | CGTGGCTCAGTTTTTCCAGC |
| ***arnC*** | CTGGTGACCTGTCTGACCAC | AGGCCCATACCGACAAACTG |
| ***arnD*** | GGTATTGCCTACGCCGATCA | CCAGTTCACCACGAACCACT |
| ***arnE*** | GAGCGTCTGGATCTGTCTGG | GCGATGCTGACCGGAATAGA |
| ***arnF*** | GCTGGTACTTTGCCCTCCAT | CCGGCCAGAAGATGGTCAAT |
| ***arnT*** | CTGAAAAAGACGCCCAGCAC | CGCCACCCAACCTAACAGAT |
| ***pmrA*** | ATCTGCGCCTTAACGTCACT | GGGCATACTCTTTCGGCGTA |
| ***pmrB*** | TGGCATGAGAGCAAAGAGCA | TGCTGATATACAGCGCCAGG |
| ***phoP*** | ATCAAGCTGACCGCCTTTGA | AAGCTGGAGCATCAGCGAAT |
| ***phoQ*** | TGCTGTGGACTCAGATGCTT | ATAGTGCAAATGCCGCTGAA |
| ***crrA*** | GTGAGGTTTCCACGCCAGTA | TTTCTCGGGTTTCTCGCCAA |
| ***crrB*** | GGCGTTTTCGAACCAGAACC | TCCTGAAGAACTCACGACGC |
| ***pmrC*** | GCGAAATGATTGCCGTCCTC | GCGGGCTTACGGATTTTCAC |

**Supplementary Table 4.** The activity of 666-15 combined with different classes of antibiotics.

|  | ***K. pneumoniae*ATCC13883** | | ***A. baumannii*ATCC19606** | | ***P. aeruginosa*ATCC27853** | |
| --- | --- | --- | --- | --- | --- | --- |
|  | **MIC (μg/mL)** | **FICI** | **MIC (μg/mL)** | **FICI** | **MIC (μg/mL)** | **FICI** |
| **Polymyxin B** | 2 | ＜0.094 | 4 | <0.281 | 2 | <0.281 |
| **Ofloxacin** | 0.5 | 2 | 0.125 | 2 | 1 | <1 |
| **Tigecycline** | 0.5 | 2 | 0.125 | 2 | NR | ～ |
| **Rifampicin** | 4 | 2 | 0.5 | 2 | 8 | 2 |
| **Meropenem** | 0.25 | 2 | 1 | 2 | 1 | 2 |
| **Gentamicin** | 2 | 2 | 8 | 2 | 8 | 2 |
| **Ceftazidime** | 0.5 | 2 | 8 | 2 | 4 | 2 |

**NR,** Natural resistance

**Supplementary Table 5.** Information of differentially expressed proteins (DEPs).

| **Protein Name** | **Description** | **Fold Change** | ***p Value*** |
| --- | --- | --- | --- |
| **GltI** | Amino acid ABC transporter substrate-binding protein | 0.001671 | 0.000004 |
| **Spy** | ATP-independent periplasmic protein-refolding chaperone | 0.00359 | 0.000001 |
| **B4U21_04180** | DUF1471 domain-containing protein | 0.00583 | 0.000014 |
| **ArtJ_1** | Arginine ABC transporter | 0.007704 | 0.000241 |
| **BANRA_00269** | ABC transporter substrate-binding protein | 0.009924 | 0.000013 |
| **FruB_3** | PTS sugar transporter subunit IIA | 0.010252 | 0.004777 |
| **Sra** | 30S ribosomal protein S22 | 0.010372 | 0.122046 |
| **OsmY** | Molecular chaperone OsmY | 0.011487 | 0.000006 |
| **IpdC** | Indolepyruvate decarboxylase | 0.015016 | 0.000299 |
| **YhbO** | DJ-1/PfpI/YhbO family deglycase/protease | 0.016654 | 0.000245 |
| **KshA_1** | 3-ketosteroid-9-alpha-hydroxylase oxygenase subunit | 0.020716 | 0.000071 |
| **B4U21_23685** | YgdI/YgdR family lipoprotein | 0.023962 | 0.000001 |
| **MglB_2** | D-galactose/ D-glucose-binding protein | 0.025705 | 0.000775 |
| **YdjH_2** | 2-dehydro-3-deoxygluconokinase | 0.027045 | 0.000082 |
| **UxuA** | Mannonate dehydratase | 0.028611 | 0.313628 |
| **BetB_1** | Aldehyde dehydrogenase | 0.029722 | 0.004171 |
| **YqjC** | DUF1090 domain-containing protein | 0.030492 | 0.167518 |
| **ArtJ** | Arginine ABC transporter substrate-binding protein | 0.0331 | 0.000001 |
| **BANRA_01420** | Uncharacterized protein | 0.033212 | 0.000029 |
| **B4U25_16840** | NAD(P)-dependent oxidoreductase | 0.033274 | 0.005543 |
| **YaiA** | Protein YaiA | 0.035658 | 0.000028 |
| **CobQ** | Cobyric acid synthase | 0.036373 | 0.307798 |
| **DppA_4** | ABC transporter substrate-binding protein | 0.03683 | 0.000017 |
| **MglB** | D-galactose-binding periplasmic protein | 0.037203 | 0.000217 |
| **ETE82_04120** | GFA family protein | 0.037358 | 0.007601 |
| **ArtM_5** | ATP-binding cassette domain-containing protein | 0.039866 | 0.000023 |
| **CybC** | Soluble cytochrome b562 | 0.040267 | 0.000663 |
| **Sad_2** | Aldehyde dehydrogenase family protein | 0.04058 | 0.000042 |
| **GltJ** | ABC transporter permease subunit | 0.042232 | 0.000314 |
| **GJJ01_27215** | UPF0229 protein GJJ01_27215 | 0.045805 | 0.000102 |
| **OtsA** | Trehalose-6-phosphate synthase | 0.04588 | 0.000027 |
| **Acs** | Acetyl-coenzyme A synthetase | 0.046508 | 0.000001 |
| **YbiH** | Putative DNA-binding transcriptional regulator | 0.047451 | 0.000989 |
| **DppA_2** | ABC transporter substrate-binding protein | 0.048822 | 0.000022 |
| **RbsB_5** | ABC transporter substrate-binding protein | 0.049067 | 0.000161 |
| **RbsB** | D-ribose ABC transporter substrate-binding protein | 0.049579 | 0.000156 |
| **TusB** | Protein TusB | 0.05003 | 0.00001 |
| **YggG_1** | Beta-barrel assembly-enhancing protease | 0.050036 | 0.000023 |
| **FadE** | Acyl-coenzyme A dehydrogenase | 0.051341 | 0.000015 |
| **CspD** | Cold shock-like protein CspD | 0.053775 | 0.000182 |
| **SprT** | Protein SprT | 0.054392 | 0.000003 |
| **B4U21_25295** | Thioredoxin | 0.054549 | 0.000206 |
| **KefG** | Glutathione-regulated potassium-efflux system ancillary protein KefG | 0.056311 | 0.000002 |
| **GJJ01_07205** | DUF3300 domain-containing protein | 0.06242 | 0.000758 |
| **SodC** | Superoxide dismutase [Cu-Zn] | 0.062479 | 0.000025 |
| **MglB_3** | D-galactose/ D-glucose-binding protein | 0.062723 | 0.001057 |
| **DsbA** | Thiol:disulfide interchange protein | 0.062776 | 0 |
| **HutU** | Urocanate hydratase | 0.062974 | 0.000049 |
| **MalX** | PTS maltose transporter subunit IICB | 0.063349 | 0.036492 |
| **DsbG** | Thiol:disulfide interchange protein | 0.064375 | 0.000027 |
| **EcnB** | Entericidin B | 0.064636 | 0.000045 |
| **DD583_25045** | Methyltransferase domain-containing protein | 0.06477 | 0.000222 |
| **PstS** | Phosphate-binding protein PstS | 0.065593 | 0.000098 |
| **MglA_3** | Ribose import ATP-binding protein RbsA | 0.066066 | 0.000354 |
| **Rmf** | Ribosome modulation factor | 0.066183 | 0.002964 |
| **KatE** | Catalase | 0.06665 | 0.000125 |
| **CsbC** | Galactose-proton symport of transport system | 0.067066 | 0.001607 |
| **FabG_13** | Dehydrogenase | 0.067371 | 0.000045 |
| **AdhP** | Alcohol dehydrogenase | 0.070142 | 0.000229 |
| **OppA** | Oligopeptide ABC transporter substrate-binding protein OppA | 0.07062 | 0.000011 |
| **GJJ01_07200** | DUF2950 family protein | 0.071349 | 0.000071 |
| **DM062_06415** | Putative homeobox protein | 0.072108 | 0.002163 |
| **BANRA_01686** | DUF2076 domain-containing protein | 0.072407 | 0.005084 |
| **B4U25_04750** | GNAT family N-acetyltransferase | 0.072425 | 0.000044 |
| **YccX** | Acylphosphatase | 0.075256 | 0.00131 |
| **DppD** | Dipeptide ABC transporter ATP-binding protein | 0.075558 | 0.001422 |
| **FXN67_18430** | L,D-transpeptidase family protein | 0.076383 | 0.000471 |
| **YciF** | DUF892 family protein | 0.077463 | 0.002821 |
| **CutA** | Divalent-cation tolerance protein CutA | 0.080044 | 0.018194 |
| **YcjI** | Murein peptide amidase A | 0.080784 | 0.000275 |
| **ProY** | Amino acid permease | 0.084507 | 0.000041 |
| **GatZ** | D-tagatose-1,6-bisphosphate aldolase subunit GatZ | 0.084571 | 0.001063 |
| **IolB** | 5-deoxy-glucuronate isomerase | 0.085689 | 0.002406 |
| **IolE** | Inosose dehydratase | 0.08571 | 0.001145 |
| **YhbO_1** | Cysteine protease YraA | 0.086116 | 0.000008 |
| **XylB_1** | Benzyl alcohol dehydrogenase | 0.089013 | 0.000049 |
| **Mak** | Fructokinase | 0.089036 | 0.000029 |
| **BANRA_02377** | DSBA oxidoreductase | 0.089353 | 0.000603 |
| **B4U21_03210** | PTS maltose transporter subunit IIBC | 0.091029 | 0.005964 |
| **YieL** | Endo-1,4-beta-xylanase A | 0.091695 | 0.000369 |
| **PpiA** | Peptidyl-prolyl cis-trans isomerase | 0.092035 | 0.000586 |
| **IolD** | 3D-(3,5/4)-trihydroxycyclohexane-1,2-dione acylhydrolase (Decyclizing) | 0.094213 | 0.000292 |
| **LldP** | L-lactate permease | 0.096046 | 0.000006 |
| **ArgT** | Amino acid ABC transporter substrate-binding protein | 0.096401 | 0.000164 |
| **HiuH** | 5-hydroxyisourate hydrolase | 0.097129 | 0.000828 |
| **HpaE** | 5-carboxymethyl-2-hydroxymuconate semialdehyde dehydrogenase | 0.097327 | 0.000081 |
| **PhoC_2** | Acid phosphatase | 0.098212 | 0.000002 |
| **MglA_3** | Galactose/methyl galactoside import ATP-binding protein MglA | 0.100734 | 0.000192 |
| **GsiA_12** | Dipeptide ABC transporter ATP binding subunit DppF | 0.101506 | 0.000062 |
| **HtpX** | Protease HtpX | 0.102835 | 0.000087 |
| **RpmF** | 50S ribosomal protein L32 | 0.10318 | 0.061282 |
| **TipA** | Albicidin resistance protein | 0.103597 | 0.000022 |
| **GmuB_2** | Oligo-beta-mannoside-specific phosphotransferase enzyme IIB component | 0.104376 | 0.000165 |
| **YcbJ** | Chromosome partition protein MukF | 0.105496 | 0.000261 |
| **RcnB_1** | Nickel/cobalt homeostasis protein RcnB | 0.105548 | 0.000597 |
| **PckA** | Phosphoenolpyruvate carboxykinase (ATP) | 0.106681 | 0.000007 |
| **BhsA_5** | DUF1471 domain-containing protein | 0.107618 | 0.005024 |
| **YfiI** | 1,5-anhydro-D-fructose reductase | 0.108173 | 0.005291 |
| **YfiN** | Diguanylate cyclase | 0.108336 | 0.402015 |
| **FadM** | 4-hydroxybenzoyl-CoA thioesterase | 0.111258 | 0.000054 |
| **B4U25_21355** | DUF2511 domain-containing protein | 0.112516 | 0.001778 |
| **YeaP_1** | Diguanylate cyclase | 0.112853 | 0.000673 |
| **YcgB** | SpoVR family protein | 0.113388 | 0.000009 |
| **MaeB** | NADP-dependent malic enzyme | 0.114726 | 0.000002 |
| **YgaC** | DUF2002 domain-containing protein | 0.115236 | 0.000045 |
| **CitW** | Citrate-sodium symporter | 0.11526 | 0.00009 |
| **GsiB** | Dipeptide-binding ABC transporter, periplasmic substrate-binding component | 0.115466 | 0.00019 |
| **GudX** | Glucarate dehydratase | 0.115741 | 0.000389 |
| **C4Y50_029165** | Four-carbon acid sugar kinase family protein | 0.116128 | 0.000134 |
| **FkpA_1** | Peptidyl-prolyl cis-trans isomerase | 0.116178 | 0.000021 |
| **Dps** | DNA protection during starvation protein | 0.116659 | 0.000327 |
| **YbaK** | Cys-tRNA(Pro)/Cys-tRNA(Cys) deacylase | 0.118793 | 0.004769 |
| **IolC** | 5-dehydro-2-deoxygluconokinase | 0.118998 | 0.001076 |
| **AmiB** | N-acetylmuramoyl-L-alanine amidase | 0.119422 | 0.000105 |
| **SerA_3** | D-3-phosphoglycerate dehydrogenase | 0.120051 | 0.000149 |
| **Mdh** | Malate dehydrogenase | 0.120216 | 0.000019 |
| **Zur** | Transcriptional regulator Zur | 0.120285 | 0.047136 |
| **XecA1** | 2-hydroxypropyl-CoM lyase | 0.120714 | 0.002391 |
| **CdsA** | Phosphatidate cytidylyltransferase | 0.121358 | 0.006032 |
| **AmiD** | AmiD protein | 0.122514 | 0.000084 |
| **Blc_2** | Outer membrane lipoprotein Blc | 0.122542 | 0.000006 |
| **HutI** | Imidazolonepropionase | 0.122636 | 0 |
| **YrdA_2** | Carbonic anhydrase, family 3 | 0.125688 | 0.005027 |
| **AmpH** | AmpH protein | 0.126059 | 0.001018 |
| **YbbA** | ABC transporter ATP-binding protein | 0.128113 | 0.000029 |
| **NudF_1** | ADP-ribose pyrophosphatase | 0.12844 | 0.001164 |
| **MepA** | Penicillin-insensitive murein endopeptidase | 0.129974 | 0.000572 |
| **YgcP** | Glycerol uptake operon antiterminator regulatory protein | 0.130497 | 0.002043 |
| **RbsC_5** | ABC transporter permease | 0.130651 | 0.000359 |
| **ElaA** | Acetyltransferase | 0.131075 | 0.000116 |
| **YbiC_1** | Malate dehydrogenase | 0.1311 | 0.000053 |
| **DD583_18315** | Transketolase (Fragment) | 0.132054 | 0.174425 |
| **DksA** | C4-type zinc finger protein, DksA/TraR family | 0.132446 | 0.000014 |
| **UspA** | Universal stress protein | 0.133034 | 0.009668 |
| **CueO** | Multicopper oxidase CueO | 0.133086 | 0.000424 |
| **PaaY** | Acetyltransferase | 0.133751 | 0.000078 |
| **TesA** | Multifunctional acyl-CoA thioesterase I/protease I/lysophospholipase L1 | 0.133803 | 0.00161 |
| **IolG** | Inositol 2-dehydrogenase | 0.133873 | 0.00009 |
| **TreA** | Periplasmic trehalase | 0.134557 | 0.000004 |
| **GJJ01_10960** | Stress-induced acidophilic repeat motif-containing protein | 0.134566 | 0.003049 |
| **OppA_2** | Oligopeptide ABC transporter substrate-binding protein OppA | 0.134681 | 0.000134 |
| **AstA** | Arginine N-succinyltransferase | 0.137582 | 0.000919 |
| **GudD** | Glucarate dehydratase | 0.139845 | 0.000321 |
| **DsdA** | D-serine dehydratase | 0.139937 | 0.002971 |
| **NdoA** | Naphthalene 1,2-dioxygenase | 0.14008 | 0.045189 |
| **HutH** | Histidine ammonia-lyase | 0.140706 | 0.000175 |
| **Pldh-t** | 3-ketoacyl-ACP reductase | 0.141067 | 0.000034 |
| **B4U21_28700** | Transcriptional regulator | 0.141411 | 0.155661 |
| **MlaC** | ABC transporter | 0.142483 | 0.000261 |
| **YciE_2** | DUF892 family protein | 0.14251 | 0.001616 |
| **Tas** | General stress protein 69 | 0.142588 | 0.000009 |
| **RffA** | dTDP-4-amino-4,6-dideoxygalactose transaminase | 0.143682 | 0.000015 |
| **YsgA** | Carboxymethylenebutenolidase | 0.143698 | 0.000647 |
| **B4U21_28790** | Lipopolysaccharide core biosynthesis protein RfaZ | 0.143889 | 0.000496 |
| **ElaB_1** | Bacterial protein of uncharacterized function (DUF883) | 0.14519 | 0.000159 |
| **B4U21_31695** | Cupin | 0.145315 | 0.000102 |
| **PfkB** | Phosphofructokinase | 0.14532 | 0.000001 |
| **BANRA_01328** | DUF2291 domain-containing protein | 0.145533 | 0.000007 |
| **CpxP** | Cell-envelope stress modulator CpxP | 0.146387 | 0.000156 |
| **NanE** | Putative N-acetylmannosamine-6-phosphate 2-epimerase | 0.146751 | 0.000201 |
| **YfgJ** | Protein of uncharacterized function (DUF1407) | 0.148253 | 0.003866 |
| **ETE82_21525** | Transporter substrate-binding domain-containing protein | 0.14851 | 0.001824 |
| **AstD** | N-succinylglutamate 5-semialdehyde dehydrogenase | 0.149129 | 0.000158 |
| **CysW_4** | ABC transporter permease subunit | 0.149181 | 0.001224 |
| **CydB_1** | Cytochrome bd oxidase subunit II | 0.150159 | 0.019519 |
| **SlyX** | Protein SlyX | 0.150364 | 0.002582 |
| **BANRA_04506** | Antibiotic biosynthesis monooxygenase | 0.153105 | 0.005023 |
| **KdpC** | Potassium-transporting ATPase KdpC subunit | 0.153591 | 0.000127 |
| **YfdX** | Protein YfdX | 0.153605 | 0.007436 |
| **HpcE_1** | 2-hydroxyhepta-2,4-diene-1,7-dioate isomerase | 0.157178 | 0.000251 |
| **MppA** | MppA protein | 0.157229 | 0.000459 |
| **GJJ01_05795** | Nucleotidyltransferase domain-containing protein | 0.157235 | 0.000105 |
| **IolH** | Glyceraldehyde-3-phosphate ketol-isomerase | 0.157266 | 0.000777 |
| **DD581_13095** | Cupin | 0.157672 | 0.006166 |
| **DksA** | RNA polymerase-binding transcription factor DksA | 0.157811 | 0.002787 |
| **B4U21_10035** | UPF0509 protein B4U21_10035 | 0.158519 | 0.000329 |
| **YgdI** | Lipoprotein | 0.159413 | 0.000038 |
| **GJJ01_03830** | Anaerobic C4-dicarboxylate transporter | 0.159598 | 0.018318 |
| **B4U21_31020** | Uncharacterized lipoprotein YifL | 0.159652 | 0.013895 |
| **C4Y50_029935** | Iron-containing redox enzyme family protein | 0.159802 | 0.000104 |
| **PpsA** | Phosphoenolpyruvate synthase | 0.160418 | 0.000004 |
| **RimK** | Probable alpha-L-glutamate ligase | 0.160821 | 0.000106 |
| **ArcB** | Aerobic respiration control sensor protein | 0.161162 | 0.000174 |
| **YniA** | Fructosamine kinase family protein | 0.161339 | 0.000262 |
| **YjbJ** | UPF0337 protein YjbJ | 0.161619 | 0.00009 |
| **Cdh** | CDP-diacylglycerol pyrophosphatase | 0.163426 | 0.00003 |
| **AtkA** | Potassium-transporting ATPase potassium-binding subunit | 0.163661 | 0.000634 |
| **OppB** | Oligopeptide ABC transporter permease OppB | 0.164365 | 0.000861 |
| **Crl** | Sigma factor-binding protein Crl | 0.164884 | 0.000005 |
| **FabG_3** | 3-oxoacyl-ACP reductase | 0.166466 | 0.003458 |
| **Fpr** | Ferredoxin (flavodoxin):NADP(+) oxidoreductase | 0.167596 | 0.000001 |
| **DctA** | C4-dicarboxylate transport protein | 0.168703 | 0.000531 |
| **CpxR** | Copper-sensing two-component system response regulator CpxR | 0.168853 | 0.001061 |
| **GJJ01_23975** | DUF1349 domain-containing protein | 0.169705 | 0.001795 |
| **B4U21_01410** | UPF0325 protein B4U21_01410 | 0.171515 | 0.00001 |
| **Blc** | Outer membrane lipoprotein Blc | 0.172641 | 0.156284 |
| **LpxO** | Aspartyl beta-hydroxylase | 0.172704 | 0.000167 |
| **AdhC2** | Alcohol dehydrogenase | 0.172728 | 0.000072 |
| **FadB** | Fatty acid oxidation complex subunit alpha | 0.172752 | 0.000007 |
| **YfcH** | Cell division inhibitor | 0.172956 | 0.000138 |
| **AphA** | Class B acid phosphatase | 0.173196 | 0.000232 |
| **MliC** | C-type lysozyme inhibitor | 0.173484 | 0.000257 |
| **ArgC** | N-acetyl-gamma-glutamyl-phosphate reductase | 0.17471 | 0.087388 |
| **AroD** | 3-dehydroquinate dehydratase | 0.174877 | 0.000062 |
| **UspG** | Universal stress protein G | 0.175575 | 0.00104 |
| **YhbH** | Ribosomal subunit interface protein | 0.176724 | 0.001549 |
| **ArnB_1** | UDP-4-amino-4-deoxy-L-arabinose--oxoglutarate aminotransferase | 0.177693 | 0.000005 |
| **HlyD** | Macrolide export protein MacA | 0.177792 | 0.000008 |
| **TbpA** | Thiamine-binding periplasmic protein | 0.177899 | 0.000349 |
| **GJJ01_12205** | Lactoylglutathione lyase | 0.178345 | 0.000027 |
| **DpiA** | Transcriptional regulatory protein | 0.178981 | 0.000249 |
| **PhoQ** | Sensor histidine protein kinase/phosphatase PhoQ | 0.180265 | 0.080482 |
| **IaaH** | Amidohydrolase | 0.180965 | 0.000399 |
| **GJJ01_20800** | Deoxyguanosinetriphosphate triphosphohydrolase | 0.182249 | 0.016328 |
| **GlnH** | GlnH protein | 0.183267 | 0.001043 |
| **HutG** | Formimidoylglutamase | 0.183531 | 0.000008 |
| **Hpd** | 3-dehydroshikimate dehydratase | 0.185409 | 0.005152 |
| **AstC_2** | Acetylornithine/succinyldiaminopimelate aminotransferase | 0.186125 | 0.000092 |
| **B4U21_09100** | Manganese catalase | 0.186133 | 0.005206 |
| **BL124_00004595** | Lipoprotein | 0.186205 | 0.022386 |
| **CurA** | Oxidoreductase YncB | 0.186656 | 0.000002 |
| **Can_2** | Carbonic anhydrase | 0.187525 | 0.00048 |
| **GhrA_1** | Glyoxylate/hydroxypyruvate reductase A | 0.187874 | 0.000034 |
| **YceI** | Polyisoprenoid-binding protein | 0.190259 | 0.001523 |
| **RseA** | Anti-sigma-E factor RseA | 0.190875 | 0 |
| **BANRA_01135** | DUF1176 domain-containing protein | 0.190988 | 0.007425 |
| **GabT** | 4-aminobutyrate--2-oxoglutarate transaminase | 0.191238 | 0.000109 |
| **DhaL** | DhaL protein | 0.191756 | 0.000101 |
| **YbhF_3** | ABC transporter multidrug efflux pump | 0.191766 | 0.000127 |
| **FadA** | 3-ketoacyl-CoA thiolase | 0.193597 | 0.000004 |
| **Eco** | Ecotin | 0.195276 | 0.000014 |
| **GstA** | Glutathione S-transferase GstA | 0.195536 | 0.007112 |
| **DhaT_2** | Alcohol dehydrogenase | 0.195795 | 0.000219 |
| **AcrR_1** | TetR family transcriptional regulator | 0.196003 | 0 |
| **Gmr** | EAL domain-containing protein | 0.196015 | 0.000621 |
| **GlnE** | Bifunctional glutamine synthetase adenylyltransferase/adenylyl-removing enzyme | 0.19752 | 0.000037 |
| **YfcF** | Glutathione S-transferase | 0.198245 | 0.00021 |
| **PncC_3** | 2-oxo-tetronate isomerase | 0.198708 | 0.000054 |
| **PpsC_1** | NAD(P)H-quinone oxidoreductase | 0.198802 | 0.000097 |
| **HslJ** | Heat shock protein HslJ | 0.200177 | 0.003696 |
| **PcaC** | 4-carboxymuconolactone decarboxylase | 0.20026 | 0.000019 |
| **B4U25_32810** | Probable metal-binding protein (DUF2387) | 0.200435 | 0.005886 |
| **GJJ01_03350** | Uncharacterized protein conserved in bacteria | 0.201863 | 0.000046 |
| **PhoH** | PhoH protein | 0.204794 | 0.000024 |
| **PspF_2** | PRD domain-containing protein | 0.204834 | 0.001352 |
| **Dpp5** | Tol-Pal system protein TolB | 0.204961 | 0.00006 |
| **ArnA** | Bifunctional polymyxin resistance protein ArnA | 0.206309 | 0.000001 |
| **YjjU** | Patatin family protein | 0.207496 | 0.004987 |
| **KdpC** | KdpC | 0.207596 | 0.000325 |
| **YeaG** | PrkA family serine protein kinase | 0.209041 | 0.000072 |
| **YmrA** | PmrA | 0.209409 | 0.003727 |
| **GlpK** | Glycerol kinase | 0.211418 | 0.000117 |
| **IolS_2** | Aldo/keto reductase | 0.211684 | 0.000031 |
| **CisY** | Citrate synthase | 0.211705 | 0.00002 |
| **UspC** | Universal stress protein | 0.211731 | 0.006422 |
| **BANRA_00130** | DUF1338 domain-containing protein | 0.212278 | 0.000278 |
| **NfnB** | NAD(P)H nitroreductase | 0.214879 | 0.000004 |
| **PuuD** | Gamma-glutamyl-gamma-aminobutyrate hydrolase | 0.215005 | 0.01501 |
| **YpdC_1** | AraC family transcriptional regulator | 0.217708 | 0.007167 |
| **B4U21_06550** | Asp/Glu/hydantoin racemase | 0.218044 | 0.000083 |
| **B4U21_28520** | Epimerase KguE | 0.219833 | 0.000095 |
| **YbjP** | Lipoprotein | 0.220158 | 0.00106 |
| **FadJ** | Fatty acid oxidation complex subunit alpha | 0.220983 | 0.00004 |
| **ModA** | Molybdate ABC transporter substrate-binding protein | 0.224048 | 0.013533 |
| **HipA** | HipA protein | 0.224857 | 0.000006 |
| **B4U21_17675** | UPF0181 protein B4U21_17675 | 0.225087 | 0.228662 |
| **ChaB** | Cation transport regulator | 0.226142 | 0.000606 |
| **ChbB** | PTS N,N'-diacetylchitobiose transporter subunit IIB | 0.227599 | 0.077862 |
| **B4U25_26945** | Uncharacterized protein | 0.229336 | 0.000592 |
| **PotA_5** | ABC transporter ATP-binding protein | 0.229987 | 0.000004 |
| **GJJ01_06250** | Amino acid deaminase | 0.230386 | 0.017711 |
| **MobB** | Molybdopterin-guanine dinucleotide biosynthesis protein B | 0.230872 | 0.294446 |
| **Azr** | Chromate reductase | 0.231012 | 0.000001 |
| **Rna** | Ribonuclease I | 0.231269 | 0.000024 |
| **Tal** | Transaldolase | 0.231802 | 0.000086 |
| **GlgX** | Glycogen debranching enzyme | 0.232366 | 0.000209 |
| **PuuC** | Aldehyde dehydrogenase PuuC | 0.234382 | 0.000087 |
| **ArsC** | Arsenate reductase | 0.238134 | 0.000639 |
| **PxpA** | 5-oxoprolinase subunit A | 0.238652 | 0.000033 |
| **GJJ01_27785** | DUF3833 family protein | 0.240155 | 0.041039 |
| **YbhB** | Kinase inhibitor | 0.240365 | 0.00011 |
| **B4U21_29030** | DUF1454 domain-containing protein | 0.241252 | 0.007096 |
| **YbaY** | Glycoprotein-polysaccharide metabolism | 0.241824 | 0.012013 |
| **LacE_3** | Glycerol-3-phosphate ABC transporter | 0.24237 | 0.00339 |
| **ElaB_1** | Bacterial protein of uncharacterized function (DUF883) | 0.242818 | 0.000058 |
| **FcuA** | Putative TonB-dependent receptor | 0.244485 | 0.221708 |
| **KdgR_2** | Helix-turn-helix domain-containing protein | 0.244708 | 0.000379 |
| **SerA** | 2-oxoglutarate reductase | 0.246688 | 0.000892 |
| **Bcr_1** | Bcr/CflA family efflux transporter | 0.247259 | 0.000715 |
| **YceF** | 7-methyl-GTP pyrophosphatase | 0.248232 | 0.000314 |
| **YbiO** | Mechanosensitive channel protein | 0.24904 | 0.000071 |
| **RutR_3** | HTH-type transcriptional repressor NicS | 0.2497 | 0.000307 |
| **SoxB_1** | D-amino acid dehydrogenase | 0.249719 | 0.000002 |
| **GltP** | Proton/glutamate-aspartate symporter | 0.250951 | 0.000082 |
| **YrdN** | 4-oxalocrotonate tautomerase | 0.251045 | 0.034569 |
| **GldA** | DhaD protein | 0.251065 | 0.00002 |
| **Fbp** | Fructose-1,6-bisphosphatase class 1 | 0.252436 | 0.000001 |
| **ArnT** | Undecaprenyl phosphate-alpha-4-amino-4-deoxy-L-arabinose arabinosyl transferase | 0.252748 | 0.099169 |
| **GlgC** | Glucose-1-phosphate adenylyltransferase | 0.253095 | 0.000017 |
| **IraP** | Anti-adapter protein IraP | 0.253141 | 0.000119 |
| **LivJ** | Branched chain amino acid ABC transporter substrate-binding protein LivJ | 0.254311 | 0.00041 |
| **GlmS_3** | Glucosamine--fructose-6-phosphate aminotransferase | 0.25487 | 0.001771 |
| **YaeP** | UPF0253 protein B4U21_01555 | 0.25525 | 0.000263 |
| **PtrA_2** | Pitrilysin | 0.255561 | 0.000743 |
| **FeaB** | Aldehyde dehydrogenase family protein | 0.257515 | 0.001038 |
| **YvoA_1** | HTH-type transcriptional repressor yvoA | 0.258225 | 0.000011 |
| **CdaR** | Carbohydrate diacid regulon transcriptional regulator CdaR | 0.258435 | 0.000001 |
| **GJJ01_17095** | AsmA family protein | 0.258459 | 0.002568 |
| **YedY** | Protein-methionine-sulfoxide reductase catalytic subunit MsrP | 0.258578 | 0.129441 |
| **G5637_15640** | Putative lyase | 0.258836 | 0.000089 |
| **GhrB** | Glyoxylate/hydroxypyruvate reductase B | 0.260594 | 0.000021 |
| **B4U21_14760** | DUF2526 domain-containing protein | 0.260803 | 0.176016 |
| **LeuB** | 3-isopropylmalate dehydrogenase | 0.26201 | 0.000001 |
| **FtsP** | Cell division protein FtsP | 0.262194 | 0.000172 |
| **PoxB** | Pyruvate dehydrogenase | 0.262724 | 0.000058 |
| **LplT** | Lysophospholipid transporter LplT | 0.263778 | 0.000353 |
| **B4U21_05280** | Zinc ribbon-containing protein | 0.265521 | 0.000006 |
| **MetC** | Cystathionine beta-lyase | 0.265561 | 0.007926 |
| **RocF** | Arginase | 0.265971 | 0.000038 |
| **GcvP** | Glycine dehydrogenase (decarboxylating) | 0.266689 | 0.000001 |
| **PutP** | Sodium/proline symporter | 0.266839 | 0.000161 |
| **GstB_3** | Glutathione S-transferase | 0.266919 | 0.000235 |
| **Gap** | Glyceraldehyde-3-phosphate dehydrogenase | 0.266993 | 0.000861 |
| **CitE2** | Citrate (pro-3S)-lyase subunit beta | 0.267458 | 0.000106 |
| **GsiA_7** | Nickel import ATP-binding protein NikE | 0.267986 | 0.130505 |
| **KduD** | 2-dehydro-3-deoxy-D-gluconate 5-dehydrogenase | 0.268555 | 0.001168 |
| **AraC_1** | Arabinose operon regulatory protein | 0.268839 | 0.00185 |
| **CitF** | Citrate lyase alpha chain | 0.269113 | 0.005947 |
| **YgfA** | 5-formyltetrahydrofolate cyclo-ligase | 0.269228 | 0.000751 |
| **BANRA_02381** | Acetyltransferase | 0.270824 | 0.000139 |
| **GsiC** | Dipeptide transport system permease DppB | 0.272105 | 0.132026 |
| **Rsd** | Regulator of sigma D | 0.275188 | 0.000057 |
| **BANRA_02298** | Divergent polysaccharide deacetylase family protein | 0.275233 | 0.003629 |
| **B4U21_29360** | Putative secreted protein | 0.275308 | 0.001217 |
| **B4U21_30240** | PTS lactose transporter subunit IIB | 0.275412 | 0.015766 |
| **YqeD** | 2-dehydro-3-deoxy-D-gluconate 5-dehydrogenase | 0.275506 | 0.001471 |
| **RpoS2** | Regulator of RpoS | 0.275956 | 0.000131 |
| **ArnC** | Undecaprenyl-phosphate 4-deoxy-4-formamido-L-arabinose transferase | 0.276279 | 0.000012 |
| **GabD_2** | Succinate-semialdehyde dehydrogenase | 0.277082 | 0.000901 |
| **XylC** | 4-aminobutyraldehyde dehydrogenase | 0.2782 | 0.002672 |
| **YfcE** | Phosphoesterase | 0.278763 | 0.000975 |
| **SbmC** | DNA gyrase inhibitor | 0.27888 | 0.000211 |
| **CysZ** | Sulfate transporter CysZ | 0.280306 | 0.000066 |
| **ScrA** | Negative regulator of SacY activity | 0.28035 | 0.017148 |
| **Por_2** | Fructuronate reductase | 0.280376 | 0.000552 |
| **OmpR** | DNA-binding response regulator | 0.280685 | 0.00008 |
| **RpoS** | RNA polymerase sigma factor RpoS | 0.280963 | 0.000741 |
| **YhaH** | DUF805 domain-containing protein | 0.281069 | 0.009303 |
| **KipI_2** | 5-oxoprolinase subunit PxpB | 0.281671 | 0.000293 |
| **YdgJ_3** | Oxidoreductase | 0.282003 | 0.012771 |
| **MmuM** | Homocysteine S-methyltransferase | 0.282291 | 0 |
| **ArnD** | Probable 4-deoxy-4-formamido-L-arabinose-phosphoundecaprenol deformylase ArnD | 0.282721 | 0.00029 |
| **DM059_04510** | Transcriptional repressor | 0.282786 | 0.007416 |
| **ProX** | Glycine betaine/L-proline ABC transporter substrate-binding protein ProX | 0.284443 | 0.029774 |
| **LsrF** | 3-hydroxy-5-phosphonooxypentane-2,4-dione thiolase | 0.284502 | 0.00428 |
| **B4U21_24985** | 26 kDa periplasmic immunogenic protein | 0.285284 | 0.000003 |
| **NhoA** | N-hydroxyarylamine O-acetyltransferase | 0.285308 | 0.000005 |
| **YciS** | Lipopolysaccharide assembly protein A | 0.285692 | 0.002002 |
| **YgdR_2** | Lipoprotein ygdR | 0.285809 | 0.000005 |
| **RlmF** | Ribosomal RNA large subunit methyltransferase F | 0.287161 | 0.007093 |
| **IolC_1** | 2-dehydro-3-deoxygluconokinase | 0.287605 | 0.000103 |
| **ChbA_2** | PTS lactose/cellobiose transporter subunit IIA | 0.287612 | 0.000023 |
| **CitB2** | Transcriptional regulatory protein | 0.288211 | 0.000071 |
| **PaaX** | Phenylacetic acid degradation operon Negative regulatory protein PaaX | 0.288615 | 0.008184 |
| **UreC** | Urease subunit alpha | 0.289176 | 0.000813 |
| **FadH** | 2,4-dienoyl-CoA reductase | 0.290094 | 0.00001 |
| **CsiE** | CsiE protein | 0.291475 | 0.069399 |
| **Pal** | Peptidoglycan-associated protein | 0.293999 | 0.001322 |
| **GatY** | D-tagatose-1,6-bisphosphate aldolase subunit GatY | 0.295352 | 0.000708 |
| **YmdB** | O-acetyl-ADP-ribose deacetylase | 0.29572 | 0.000001 |
| **BglX** | Beta-D-glucoside glucohydrolase | 0.295869 | 0.000002 |
| **D1220_26550** | C-lysozyme inhibitor | 0.296016 | 0.000109 |
| **AhpC** | Alkyl hydroperoxide reductase C | 0.297206 | 0.000987 |
| **OpuBC** | ABC transporter substrate-binding protein | 0.298853 | 0.000152 |
| **FepB** | Fe2+-enterobactin ABC transporter substrate-binding protein | 0.300961 | 0.020664 |
| **GdhA_1** | Glutamate dehydrogenase | 0.301161 | 0.000024 |
| **PtsH** | Phosphocarrier protein HPr | 0.302172 | 0.0198 |
| **YpwA** | Metal-dependent carboxypeptidase | 0.302518 | 0.000008 |
| **YiaD** | Inner membrane lipoprotein YiaD | 0.30515 | 0.000642 |
| **GJJ01_02240** | Glutathione S-transferase | 0.305434 | 0.010971 |
| **SurA** | Chaperone SurA | 0.306148 | 0.000001 |
| **B4U21_08235** | DUF1311 domain-containing protein | 0.307647 | 0.002 |
| **YdcF** | Protein ydcF | 0.308248 | 0.000094 |
| **YbdG_2** | Mechanosensitive ion channel | 0.308515 | 0.006787 |
| **YqjG** | Glutathione S-transferase | 0.308625 | 0.000088 |
| **SucD** | Succinate--CoA ligase [ADP-forming] subunit alpha | 0.310071 | 0.000015 |
| **AttT** | AttT protein | 0.311615 | 0.005462 |
| **EmtA** | Endo-type membrane-bound lytic murein transglycosylase A | 0.31284 | 0.0194 |
| **GJJ01_02105** | HAD-IC family P-type ATPase | 0.313046 | 0.000014 |
| **DhaK_2** | Dihydroxyacetone kinase, ATP-dependent | 0.315617 | 0.000037 |
| **PspA** | Phage shock protein A | 0.316038 | 0.001437 |
| **YdgH** | DUF1471 domain-containing protein | 0.316345 | 0.000103 |
| **YtfK** | DUF1107 domain-containing protein | 0.316478 | 0.057832 |
| **RihC** | Non-specific ribonucleoside hydrolase RihC | 0.316494 | 0.006557 |
| **SltY** | Peptidoglycan lytic exotransglycosylase | 0.318498 | 0.000029 |
| **ErfK** | L,D-transpeptidase | 0.318876 | 0.000356 |
| **YncE_2** | Putative receptor | 0.318995 | 0.000006 |
| **IlvN** | Acetolactate synthase | 0.319224 | 0.00595 |
| **CopA** | Copper-exporting P-type ATPase A | 0.321082 | 0.020756 |
| **PtsP** | Phosphoenolpyruvate--protein phosphotransferase | 0.321585 | 0.020539 |
| **AtpE** | ATP synthase subunit c | 0.322608 | 0.065129 |
| **YcaC_2** | Amidohydrolase | 0.322634 | 0.190808 |
| **YbdK** | Putative glutamate--cysteine ligase 2 | 0.322726 | 0.004366 |
| **YheI** | ABC transporter ATP-binding protein | 0.325952 | 0.000194 |
| **DmlR_2** | D-malate degradation protein R | 0.32617 | 0.045564 |
| **B4U21_31735** | Thiamin phosphate synthase | 0.326684 | 0.016301 |
| **YqcA** | Flavodoxin | 0.326869 | 0.000034 |
| **DmlR_15** | LysR family transcriptional regulator | 0.327893 | 0.238262 |
| **ETE82_15245** | SIS domain-containing protein | 0.330463 | 0.007802 |
| **DsbD_2** | Cytochrome c-type biogenesis protein DsbD | 0.33067 | 0.001358 |
| **TraT** | TraT complement resistance protein | 0.330683 | 0.001957 |
| **PuuE** | 4-aminobutyrate--2-oxoglutarate transaminase | 0.330712 | 0.000672 |
| **YgaD_1** | C-terminal domain of CinA type S | 0.33137 | 0.004088 |
| **YjjV** | Deoxyribonuclease | 0.331484 | 0.006685 |
| **CydA_1** | Cytochrome bd-I ubiquinol oxidase subunit 1 | 0.332082 | 0.000497 |
| **UreE** | Urease accessory protein UreE | 0.332851 | 0.000126 |
| **B4U21_29690** | Outer membrane lipoprotein YidQ | 0.332876 | 0.00309 |
| **B4U21_15465** | CadC family transcriptional regulator | 0.333616 | 0.000506 |
| **B4U25_27875** | DUF4440 domain-containing protein | 0.336379 | 0.000375 |
| **NpdA** | NAD-dependent protein deacylase | 0.336451 | 0.000033 |
| **DppA_5** | ABC transporter substrate-binding protein | 0.33744 | 0.005263 |
| **PpnP** | Pyrimidine/purine nucleoside phosphorylase | 0.337571 | 0.000346 |
| **CfxP** | Phosphoribulokinase | 0.337826 | 0.000384 |
| **Dcp** | Dipeptidyl carboxypeptidase Dcp | 0.338667 | 0.000484 |
| **GmuD_2** | 6-phospho-beta-glucosidase | 0.339183 | 0.010975 |
| **GsiA_14** | ABC transporter ATP-binding protein | 0.339885 | 0.087936 |
| **AndAa** | Anthranilate 1,2-dioxygenase system ferredoxin--NAD(+) reductase component | 0.340029 | 0.000697 |
| **GcvT** | Aminomethyltransferase | 0.340591 | 0.000012 |
| **GJJ01_06285** | GNAT family N-acetyltransferase | 0.340983 | 0.010077 |
| **YnhG** | L,D-transpeptidase YnhG | 0.341107 | 0.000072 |
| **LimB_2** | LLM class flavin-dependent oxidoreductase | 0.341132 | 0.000273 |
| **FrsA** | Esterase FrsA | 0.341294 | 0.000089 |
| **NfuA** | Fe/S biogenesis protein NfuA | 0.341946 | 0.000078 |
| **TilS** | tRNA(Ile)-lysidine synthase | 0.342665 | 0.000013 |
| **ApbE_1** | FAD:protein FMN transferase | 0.343985 | 0.004242 |
| **BANRA_02142** | DUF2756 domain-containing protein | 0.344665 | 0.07231 |
| **PldA** | Phospholipase A1 | 0.345747 | 0.054895 |
| **GltK_1** | Amino acid ABC transporter permease | 0.346488 | 0.003309 |
| **BANRA_03358** | Inner membrane protein YqjK | 0.346909 | 0.030048 |
| **yhcB** | Cytochrome d ubiquinol oxidase subunit 3 | 0.348007 | 0.000015 |
| **sodA** | Superoxide dismutase | 0.348201 | 0.002054 |
| **ridA_2** | Endoribonuclease L-PSP | 0.34847 | 0.002043 |
| **efeO** | Iron uptake system component EfeO | 0.348689 | 0.000253 |
| **YdiB_1** | Shikimate 5-dehydrogenase | 0.348752 | 0.027415 |
| **HpaG** | 2-hydroxyhepta-2,4-diene-1,7-dioate isomerase | 0.349276 | 0.006786 |
| **Aat** | Leucyl/phenylalanyl-tRNA--protein transferase | 0.35105 | 0.001574 |
| **YihX** | Alpha-D-glucose 1-phosphate phosphatase YihX | 0.351938 | 0.000187 |
| **FdhF_1** | CbbBc protein | 0.35248 | 0.014027 |
| **AcnB** | Aconitate hydratase B | 0.353133 | 0 |
| **YccF** | Inner membrane protein YccF | 0.353139 | 0.002339 |
| **BglG** | Beta-glucoside operon transcriptional antiterminator | 0.353332 | 0.01136 |
| **GJJ01_00275** | Methyltransferase domain-containing protein | 0.353669 | 0.00749 |
| **MsrB** | Peptide methionine sulfoxide reductase MsrB | 0.353908 | 0.004879 |
| **AcrB_3** | Efflux pump membrane transporter | 0.355919 | 0.000712 |
| **OsmY_3** | BON domain-containing protein | 0.356398 | 0.000398 |
| **ApaG** | Protein ApaG | 0.35722 | 0.000157 |
| **YjcE** | Na(+)/H(+) exchanger protein | 0.357545 | 0.000337 |
| **RffM** | UDP-N-acetyl-D-mannosaminuronic acid transferase | 0.358713 | 0.00041 |
| **B4U21_14460** | TetR family transcriptional regulator | 0.359794 | 0.000999 |
| **SucC** | Succinate--CoA ligase [ADP-forming] subunit beta | 0.360304 | 0.000001 |
| **AceB** | Malate synthase | 0.361172 | 0.021739 |
| **YccU** | CoA-binding protein | 0.361626 | 0.000335 |
| **SlyA_1** | MarR family transcriptional regulator | 0.361641 | 0.000263 |
| **HemF** | Oxygen-dependent coproporphyrinogen-III oxidase | 0.362165 | 0.179324 |
| **FumC** | Fumarate hydratase class II | 0.362902 | 0.003778 |
| **BglT** | Glycoside hydrolase | 0.36319 | 0.006915 |
| **GloB** | Hydroxyacylglutathione hydrolase | 0.363659 | 0.000657 |
| **BglH_2** | 6-phospho-beta-glucosidase | 0.363985 | 0.003727 |
| **CpoB** | Cell division coordinator CpoB | 0.364086 | 0.000529 |
| **YciI** | YciI family protein | 0.364341 | 0.009834 |
| **SthA** | Soluble pyridine nucleotide transhydrogenase | 0.366483 | 0.000865 |
| **B4U21_32950** | Putative cytoplasmic protein | 0.366589 | 0.412813 |
| **RibE** | Riboflavin synthase | 0.366667 | 0.000027 |
| **ArtP** | Arginine ABC transporter ATP-binding protein ArtP | 0.366942 | 0.000278 |
| **PaaF** | Phenylacetate-coenzyme A ligase | 0.368538 | 0.00244 |
| **YxeP_5** | Amidohydrolase | 0.3687 | 0.022233 |
| **GstB_3** | Glutathione S-transferase | 0.368712 | 0.000191 |
| **RbnZ** | Ribonuclease BN | 0.368993 | 0.004316 |
| **AcnA** | Aconitate hydratase | 0.369062 | 0.000066 |
| **MprA** | McrB protein | 0.369515 | 0.001124 |
| **YjaB_1** | Acetyltransferase | 0.369974 | 0.000327 |
| **SppA** | Protease 4 | 0.370199 | 0.000067 |
| **YoaC** | DUF1889 domain-containing protein | 0.371009 | 0.002705 |
| **YfbT** | Putative phosphatase | 0.371048 | 0.00012 |
| **YajI** | DUF3251 domain-containing protein | 0.372091 | 0.000286 |
| **Skp** | Chaperone protein Skp | 0.372362 | 0.000305 |
| **YfbU** | UPF0304 protein B4U21_20295 | 0.372468 | 0.000023 |
| **FadR_1** | HTH-type transcriptional regulator BetI | 0.372838 | 0.001507 |
| **RamA_2** | Amidase | 0.372939 | 0.068495 |
| **YfeY** | Outer membrane lipoprotein YfeY | 0.373437 | 0.000015 |
| **Ggt** | Gamma-glutamyltransferase | 0.37387 | 0.000103 |
| **OdhB** | Dihydrolipoyllysine-residue succinyltransferase component of 2-oxoglutarate dehydrogenase complex | 0.374159 | 0.00022 |
| **GlpD** | Glycerol-3-phosphate dehydrogenase | 0.374481 | 0.000018 |
| **FdxH** | Formate dehydrogenase iron-sulfur subunit | 0.374766 | 0.000441 |
| **CutC** | Copper homeostasis protein CutC | 0.374921 | 0.000146 |
| **AsnB** | Asparagine synthase (glutamine-hydrolyzing) | 0.375172 | 0 |
| **DmlA** | D-malate dehydrogenase [decarboxylating] | 0.376004 | 0.000048 |
| **BsaA_1** | Thioredoxin/glutathione peroxidase BtuE | 0.376591 | 0.000015 |
| **CysQ** | 3'(2'),5'-bisphosphate nucleotidase CysQ | 0.377125 | 0.000193 |
| **RihA** | Pyrimidine-specific ribonucleoside hydrolase RihA | 0.37825 | 0.000002 |
| **LdcC** | Lysine decarboxylase 2, constitutive | 0.379757 | 0.000024 |
| **CodA_2** | Cytosine deaminase | 0.379798 | 0.125254 |
| **RbsD** | D-ribose pyranase | 0.381135 | 0.13485 |
| **B4U21_10820** | Uncharacterized protein | 0.381155 | 0.089169 |
| **RdoA** | Stress response kinase A | 0.381546 | 0.000068 |
| **DgkA** | Diacylglycerol kinase | 0.38208 | 0.154268 |
| **B4U21_33640** | Exported protein | 0.382195 | 0.001469 |
| **CbiH** | Cobalt-precorrin-3B C(17)-methyltransferase | 0.382197 | 0.000294 |
| **ProB** | Glutamate 5-kinase | 0.383234 | 0.165574 |
| **FadI** | 3-ketoacyl-CoA thiolase | 0.38434 | 0.002307 |
| **LspA** | Lipoprotein signal peptidase | 0.384471 | 0.404363 |
| **YnfB** | UPF0482 protein B4U21_12195 | 0.38559 | 0.00073 |
| **OmpA** | Integral membrane protein YfiB | 0.386246 | 0.005779 |
| **YbbN** | Co-chaperone YbbN | 0.386605 | 0.000192 |
| **AhpF** | Alkyl hydroperoxide reductase subunit F | 0.387389 | 0.00004 |
| **LacI_2** | Lac operon transcriptional repressor | 0.387681 | 0.000374 |
| **Glk** | Glucokinase | 0.389488 | 0.000062 |
| **YceB** | Lipoprotein | 0.391371 | 0.000121 |
| **GarD_1** | Altronate dehydratase | 0.392183 | 0.016863 |
| **ETE82_06505** | Iron-containing alcohol dehydrogenase | 0.393371 | 0.000226 |
| **YtfL** | DUF21 domain-containing protein | 0.393388 | 0.000081 |
| **PhoP** | DNA-binding transcriptional regulator PhoP | 0.394789 | 0.000146 |
| **RseB** | Anti-sigma E factor | 0.394852 | 0.000054 |
| **UshA** | Bifunctional UDP-sugar hydrolase/5'-nucleotidase | 0.396035 | 0.000728 |
| **Rof** | Rho-binding antiterminator | 0.396266 | 0.000763 |
| **Tag** | 3-methyl-adenine DNA glycosylase I | 0.396722 | 0.000785 |
| **NrdF** | Ribonucleoside-diphosphate reductase subunit beta | 0.397475 | 0.073457 |
| **DmlR_9** | LysR family transcriptional regulator | 0.397901 | 0.023969 |
| **YobH** | Uncharacterized protein YobH | 0.397993 | 0.003101 |
| **PecS** | MarR family transcriptional regulator | 0.398079 | 0.074282 |
| **PcaF** | 3-oxoadipyl-CoA thiolase | 0.398885 | 0.001482 |
| **LuxS** | S-ribosylhomocysteine lyase | 0.398933 | 0.000126 |
| **B4U25_18720** | DNA-binding response regulator | 0.401896 | 0.000431 |
| **QorA_1** | Bifunctional zinc-containing alcohol dehydrogenase/quinone oxidoreductase | 0.403854 | 0.001136 |
| **GJJ01_02085** | MBL fold metallo-hydrolase | 0.404196 | 0.300604 |
| **NfsA** | NADPH-dependent oxidoreductase | 0.405275 | 0.075248 |
| **DkgA** | 2,5-didehydrogluconate reductase DkgA | 0.406031 | 0.021013 |
| **Dxs_3** | Transketolase | 0.406824 | 0.000362 |
| **CreB** | DNA-binding response regulator | 0.407024 | 0.008601 |
| **Idi** | Isopentenyl-diphosphate Delta-isomerase | 0.407028 | 0.000858 |
| **PdxH** | Pyridoxine/pyridoxamine 5'-phosphate oxidase | 0.407436 | 0.000274 |
| **LeuA_1** | 2-isopropylmalate synthase | 0.408463 | 0.031444 |
| **CodB_2** | Cytosine permease | 0.408603 | 0.002934 |
| **GJJ01_19385** | Dihydrofolate reductase | 0.409227 | 0.009147 |
| **PepQ** | Xaa-Pro dipeptidase | 0.40929 | 0.000004 |
| **DhaK2** | DhaK2 protein | 0.409488 | 0.000071 |
| **CycA2** | CycA2 protein | 0.410425 | 0.003734 |
| **Mqo** | Probable malate:quinone oxidoreductase | 0.41068 | 0.000037 |
| **PuuB** | FAD-binding oxidoreductase | 0.411362 | 0.002167 |
| **RfaG** | Glycosyltransferase | 0.411794 | 0.000217 |
| **SseA** | Sulfurtransferase | 0.412166 | 0.000074 |
| **BANRA_00128** | E3 ubiquitin--protein ligase | 0.41246 | 0.000169 |
| **B4U21_02160** | L,D-transpeptidase family protein | 0.412744 | 0.012824 |
| **OppD** | ABC transporter ATP-binding protein | 0.413792 | 0.000661 |
| **PrlC** | Oligopeptidase A | 0.414021 | 0.00001 |
| **KefF** | Glutathione-regulated potassium-efflux system ancillary protein KefF | 0.414126 | 0.008596 |
| **PtsO** | HPr family phosphocarrier protein | 0.415529 | 0.070997 |
| **OprM_2** | Efflux transporter outer membrane subunit | 0.415795 | 0.210517 |
| **LptA** | Lipopolysaccharide export system protein LptA | 0.416125 | 0.023737 |
| **OsmY_2** | Divisome-associated lipoprotein YraP | 0.416285 | 0.000543 |
| **IcaR** | TetR family transcriptional regulator | 0.417263 | 0.00001 |
| **bcp** | Thioredoxin peroxidase | 0.417577 | 0.000023 |
| **BVX91_22805** | ISNCY family transposase | 0.417618 | 0.046062 |
| **AspA** | Aspartate ammonia-lyase | 0.418893 | 0.000018 |
| **Fic_1** | Cell filamentation protein Fic | 0.420071 | 0.011076 |
| **YgdG** | Flap endonuclease Xni | 0.421141 | 0.404513 |
| **TrpA** | Tryptophan synthase alpha chain | 0.422108 | 0.002934 |
| **YbgI** | GTP cyclohydrolase 1 type 2 | 0.422122 | 0.000285 |
| **MraZ** | Transcriptional regulator MraZ | 0.422278 | 0.000286 |
| **GabD_1** | NADP-dependent succinate-semialdehyde dehydrogenase | 0.422366 | 0.002067 |
| **PabC** | Aminodeoxychorismate lyase | 0.423101 | 0.004305 |
| **OpgB** | Phosphatidylglycerol--membrane-oligosaccharide glycerophosphotransferase | 0.423354 | 0.000251 |
| **GrxC** | Glutaredoxin | 0.42381 | 0.000198 |
| **TrpC** | Multifunctional fusion protein | 0.423994 | 0.005084 |
| **YeaD** | Putative glucose-6-phosphate 1-epimerase | 0.42557 | 0.000001 |
| **MdtE_2** | Efflux RND transporter periplasmic adaptor subunit | 0.426114 | 0.077934 |
| **WecF** | TDP-N-acetylfucosamine:lipid II N-acetylfucosaminyltransferase | 0.42623 | 0.000728 |
| **YhjH_1** | Cyclic di-GMP phosphodiesterase YhjH | 0.426835 | 0.000617 |
| **RppH** | RNA pyrophosphohydrolase | 0.427375 | 0.000228 |
| **UspA_2** | Universal stress protein | 0.427546 | 0.001298 |
| **YqhD** | Alcohol dehydrogenase | 0.427732 | 0.022365 |
| **DD581_14885** | Acetyltransferase | 0.427964 | 0.000035 |
| **B4U21_15085** | RND transporter | 0.428631 | 0.128534 |
| **GcvR** | Glycine cleavage system transcriptional repressor | 0.429137 | 0.019015 |
| **GpmB** | Probable phosphoglycerate mutase GpmB | 0.429946 | 0.000211 |
| **YcgE_2** | HTH-type transcriptional regulator MlrA | 0.430791 | 0.17532 |
| **BANRA_02356** | AsmA family protein | 0.430924 | 0.000064 |
| **OsmE** | DNA-binding transcriptional activator OsmE | 0.431205 | 0.002228 |
| **Icd** | Isocitrate dehydrogenase [NADP] | 0.431344 | 0.000005 |
| **PpiB** | Peptidyl-prolyl cis-trans isomerase | 0.431514 | 0.003876 |
| **G7Z27_14405** | Dimethyl sulfoxide reductase subunit A | 0.432379 | 0.064307 |
| **MlaB** | Lipid asymmetry maintenance protein MlaB | 0.432383 | 0.000792 |
| **KdpE** | DNA-binding response regulator | 0.433096 | 0.000228 |
| **DcuA** | Anaerobic C4-dicarboxylate transporter | 0.435171 | 0.10123 |
| **NuoK** | NADH-quinone oxidoreductase subunit K | 0.435709 | 0.00127 |
| **TatA** | Sec-independent protein translocase protein TatA | 0.438741 | 0.000059 |
| **YecD** | Hydrolase | 0.439175 | 0.000002 |
| **PyrG_1** | CTP synthase (glutamine hydrolyzing) | 0.439315 | 0.000116 |
| **AraB_1** | FGGY-family pentulose kinase | 0.439343 | 0.025738 |
| **TktB_1** | Transketolase | 0.439585 | 0.000066 |
| **SpeG** | SpeG protein | 0.439642 | 0.070322 |
| **DeoC** | Deoxyribose-phosphate aldolase | 0.439704 | 0.041886 |
| **DegP** | Periplasmic serine endoprotease DegP-like | 0.439717 | 0.000021 |
| **YhaK** | Pirin family protein | 0.441034 | 0.008557 |
| **YciO** | Threonylcarbamoyl-AMP synthase | 0.44147 | 0.004299 |
| **MtlD** | Mannitol-1-phosphate 5-dehydrogenase | 0.441816 | 0.034971 |
| **FucA_3** | Aldolase | 0.442625 | 0.009996 |
| **NhaA** | Na(+)/H(+) antiporter NhaA | 0.442927 | 0.02608 |
| **GJJ01_01620** | FtsX-like permease family protein | 0.443085 | 0.13899 |
| **Hda** | DnaA regulatory inactivator Hda | 0.443399 | 0.001562 |
| **RlmA** | 23S rRNA (Guanine(745)-N(1))-methyltransferase | 0.443781 | 0.009748 |
| **PhrB** | Deoxyribodipyrimidine photo-lyase | 0.44414 | 0.075023 |
| **AldA** | Aldehyde dehydrogenase | 0.445348 | 0.003547 |
| **GdhA_1** | Glutamate dehydrogenase | 0.445993 | 0.000367 |
| **B4U21_29825** | Phosphopantetheinyl transferase | 0.446303 | 0.01156 |
| **GvcA** | Gcv operon activator | 0.446733 | 0.006813 |
| **YfcD** | Uncharacterized Nudix hydrolase YfcD | 0.446895 | 0.000956 |
| **GcvH** | Glycine cleavage system H protein | 0.447315 | 0.003342 |
| **Pat** | GNAT family N-acetyltransferase | 0.447763 | 0.024326 |
| **AscG_2** | HTH-type transcriptional regulator AscG | 0.449206 | 0.004024 |
| **YybR_1** | HxlR family transcriptional regulator | 0.449502 | 0.072723 |
| **YajL** | DJ-1 family protein | 0.450154 | 0.000182 |
| **B4U21_25860** | Siderophore-interacting protein | 0.450312 | 0.00006 |
| **NudL** | Uncharacterized Nudix hydrolase NudL | 0.450464 | 0.001043 |
| **YdiA** | Putative phosphoenolpyruvate synthase regulatory protein | 0.451468 | 0.004276 |
| **UlaD** | 3-keto-L-gulonate-6-phosphate decarboxylase | 0.451543 | 0.024018 |
| **BANRA_02140** | Ketosteroid isomerase-related protein | 0.4516 | 0.002138 |
| **TrmJ** | tRNA (cytidine/uridine-2'-O-)-methyltransferase TrmJ | 0.452452 | 0.000466 |
| **IlvA** | L-threonine dehydratase | 0.452619 | 0.003169 |
| **YfbR** | 5'-deoxynucleotidase B6R99_06510 | 0.455124 | 0.029196 |
| **MmsA** | 3-oxopropanoate dehydrogenase | 0.456801 | 0.008746 |
| **RimJ** | 30S ribosomal protein S5 alanine N-acetyltransferase | 0.457504 | 0.044955 |
| **CysK** | Cysteine synthase | 0.457785 | 0.000083 |
| **CobD** | L-threonine-O-3-phosphate decarboxylase | 0.45802 | 0.00308 |
| **ArcA** | Aerobic respiration control protein arcA | 0.458287 | 0.000196 |
| **HexR_3** | DNA-binding transcriptional regulator HexR | 0.458367 | 0.025028 |
| **ETE94_29105** | GTP-binding protein | 0.458556 | 0.001261 |
| **GppA** | Guanosine-5'-triphosphate,3'-diphosphate pyrophosphatase | 0.459641 | 0.000368 |
| **YnfD** | DUF1161 domain-containing protein | 0.460565 | 0.06912 |
| **CobA** | Siroheme synthase | 0.460779 | 0.003365 |
| **Amn** | AMP nucleosidase | 0.461368 | 0.000094 |
| **CpdB** | 2',3'-cyclic-nucleotide 2'-phosphodiesterase/3'-nucleotidase | 0.461766 | 0.011031 |
| **NamA** | FMN oxidoreductase | 0.462022 | 0.000371 |
| **MtnD** | Acireductone dioxygenase | 0.463043 | 0.0054 |
| **CobO** | Corrinoid adenosyltransferase | 0.464587 | 0.002969 |
| **PbpD** | Biosynthetic peptidoglycan transglycosylase | 0.464771 | 0.001766 |
| **EmrA** | Inner membrane protein yibH | 0.46556 | 0.000282 |
| **PutA** | Bifunctional protein PutA | 0.465794 | 0.00005 |
| **YiiM** | 6-N-hydroxylaminopurine resistance protein | 0.465908 | 0.003479 |
| **DsbC** | Thiol:disulfide interchange protein | 0.466553 | 0.000605 |
| **CreA** | Protein CreA | 0.467089 | 0.004446 |
| **YfiC** | tRNA1(Val) (adenine(37)-N6)-methyltransferase | 0.468219 | 0.040278 |
| **Rnk** | Regulator of nucleoside diphosphate kinase | 0.468673 | 0.000019 |
| **B4U21_28050** | Phenolic acid decarboxylase | 0.470316 | 0.035181 |
| **B4U21_13655** | ASCH domain-containing protein | 0.47062 | 0.016167 |
| **GlpQ** | Glycerophosphodiester phosphodiesterase | 0.471464 | 0.000138 |
| **GJJ01_14605** | 2-dehydropantoate 2-reductase | 0.471567 | 0.030523 |
| **MtnA** | Methylthioribose-1-phosphate isomerase | 0.471777 | 0.009656 |
| **YhcM_1** | Cell division protein ZapE | 0.472126 | 0.00009 |
| **CysM** | Cysteine synthase | 0.473175 | 0.007024 |
| **YjgR** | DUF853 family protein | 0.47322 | 0.00014 |
| **RhaR** | HTH-type transcriptional activator RhaR | 0.474109 | 0.091166 |
| **HcaR_2** | LysR family transcriptional regulator | 0.475975 | 0.016147 |
| **NlpD** | Lipoprotein NlpD | 0.476077 | 0.00006 |
| **PepP** | Xaa-Pro aminopeptidase | 0.476365 | 0.009683 |
| **RfbA** | Glucose-1-phosphate thymidylyltransferase | 0.47649 | 0.001009 |
| **IhfB** | Integration host factor subunit beta | 0.478014 | 0.000561 |
| **GsiA_1** | Dipeptide transport ATP-binding protein DppD | 0.478341 | 0.137526 |
| **SrlE_2** | PTS glucitol/sorbitol transporter subunit IIB | 0.48015 | 0.000535 |
| **B4U25_39520** | Transcriptional regulator | 0.481252 | 0.002626 |
| **OxyR_1** | LysR family transcriptional regulator | 0.481324 | 0.005454 |
| **GalK** | Galactokinase | 0.481765 | 0.000385 |
| **Sdh** | Oxidoreductase | 0.481772 | 0.236396 |
| **RidA_1** | 2-iminobutanoate/2-iminopropanoate deaminase | 0.482109 | 0.000604 |
| **YqeF** | 3-ketoacyl-CoA thiolase | 0.482941 | 0.021869 |
| **UxaB** | Altronate oxidoreductase | 0.48343 | 0.292845 |
| **PspB** | Envelope stress response membrane protein PspB | 0.483576 | 0.001197 |
| **DcrB** | DUF1795 domain-containing protein | 0.484024 | 0.000924 |
| **ETE82_15865** | Formate dehydrogenase | 0.484358 | 0.017954 |
| **ManY_3** | Mannose/fructose/sorbose family PTS transporter subunit IIC | 0.484584 | 0.000144 |
| **NuoN** | NADH-quinone oxidoreductase subunit N | 0.484821 | 0.000949 |
| **YjjA** | DUF2501 domain-containing protein | 0.486374 | 0.000271 |
| **AspC** | Aminotransferase | 0.487582 | 0.000011 |
| **B4U25_17585** | DUF3313 domain-containing protein | 0.487976 | 0.007484 |
| **PgsA** | CDP-diacylglycerol--glycerol-3-phosphate 3-phosphatidyltransferase | 0.488136 | 0.001572 |
| **AsnA** | Aspartate--ammonia ligase | 0.488191 | 0.000178 |
| **AaeR** | D-malate degradation protein R | 0.488227 | 0.010231 |
| **SucA** | Oxoglutarate dehydrogenase (succinyl-transferring) | 0.490007 | 0.000001 |
| **YggN** | DUF2884 domain-containing protein | 0.490266 | 0.000418 |
| **SinR_2** | Cupin domain-containing protein | 0.490389 | 0.002384 |
| **YdjH_2** | 2-dehydro-3-deoxygluconokinase | 0.49041 | 0.000453 |
| **GlpR_1** | DeoR family transcriptional regulator | 0.490536 | 0.00113 |
| **PepN** | Aminopeptidase N | 0.491031 | 0.000024 |
| **SmpA** | Outer membrane protein assembly factor BamE | 0.491761 | 0.000513 |
| **HupB** | DNA-binding protein HU-beta | 0.492573 | 0.000128 |
| **LldD** | L-lactate dehydrogenase | 0.492861 | 0.000047 |
| **RutR** | HTH-type transcriptional regulator RutR | 0.493647 | 0.003806 |
| **YcgF_1** | BLUF domain/cyclic diguanylate phosphodiesterase (EAL) domain protein | 0.49367 | 0.0001 |
| **MoeB** | Molybdopterin biosynthesis protein MoeB | 0.49378 | 0.144415 |
| **CvpA** | Colicin V production protein | 0.494108 | 0.001948 |
| **YecE** | DUF72 domain-containing protein | 0.494663 | 0.000379 |
| **Dld** | Quinone-dependent D-lactate dehydrogenase | 0.495997 | 0.000134 |
| **B4U21_09230** | DUF488 domain-containing protein | 0.496924 | 0.000469 |
| **YafJ** | Class II glutamine amidotransferase | 0.497255 | 0.000068 |
| **RaiA** | Ribosomal subunit interface protein | 0.498398 | 0.000128 |
| **TreB** | EIIBC-Tre | 0.500172 | 0.003413 |
| **CytR** | DNA-binding transcriptional regulator CytR | 0.500292 | 0.000318 |
| **TpiA** | Triosephosphate isomerase | 0.50058 | 0.003283 |
| **UspE** | Universal stress protein E | 0.50187 | 0.000011 |
| **B4U21_07625** | DNA-binding protein | 0.501997 | 0.00126 |
| **YbiC_2** | Ldh family oxidoreductase | 0.502363 | 0.022313 |
| **UreG** | Urease accessory protein UreG | 0.503105 | 0.003728 |
| **LysA** | Diaminopimelate decarboxylase | 0.504599 | 0.00429 |
| **YggG_2** | M48 family metalloprotease | 0.504867 | 0.000467 |
| **GarR** | 2-hydroxy-3-oxopropionate reductase | 0.505423 | 0.000298 |
| **B4U25_41320** | DUF2164 domain-containing protein | 0.506877 | 0.232528 |
| **AldB_2** | Aldehyde dehydrogenase | 0.507283 | 0.00514 |
| **TorZ** | Biotin sulfoxide reductase | 0.50833 | 0.041373 |
| **GshA** | Glutamate--cysteine ligase | 0.508631 | 0.00017 |
| **HisJ** | Histidine ABC transporter | 0.509931 | 0.000169 |
| **TopA_1** | DNA topoisomerase 1 | 0.512242 | 0.01655 |
| **B4U21_05600** | DUF2517 domain-containing protein | 0.512443 | 0.041222 |
| **YgdH** | AMP nucleosidase | 0.512544 | 0.000128 |
| **C2U49_27415** | DNA-binding transcriptional regulator | 0.512934 | 0.005892 |
| **YgeA** | Aspartate racemase | 0.512938 | 0.000957 |
| **ElbB** | Glyoxalase | 0.51423 | 0.000071 |
| **GalR** | Galactose operon repressor | 0.514414 | 0.001641 |
| **ThrA** | Bifunctional aspartokinase/homoserine dehydrogenase | 0.514435 | 0.00025 |
| **YgjG** | Putrescine aminotransferase | 0.514599 | 0.126611 |
| **IlvN** | Acetolactate synthase | 0.516071 | 0.003768 |
| **PdxY** | Pyridoxal kinase PdxY | 0.516967 | 0.023053 |
| **FolA** | Dihydrofolate reductase | 0.517338 | 0.000648 |
| **DacC** | Serine-type D-Ala-D-Ala carboxypeptidase | 0.518416 | 0.000027 |
| **HspQ** | Heat shock protein HspQ | 0.518507 | 0.07358 |
| **GlpG** | Rhomboid protease GlpG | 0.518593 | 0.005168 |
| **RbsK** | Ribokinase | 0.519304 | 0.028088 |
| **Rpe** | Ribulose-phosphate 3-epimerase | 0.520248 | 0.001153 |
| **YciH** | Stress response translation initiation inhibitor YciH | 0.520398 | 0.018969 |
| **WecB** | UDP-N-acetylglucosamine 2-epimerase | 0.520454 | 0.002019 |
| **SelD** | Selenide, water dikinase | 0.520473 | 0.000225 |
| **B4U21_11670** | DUF945 domain-containing protein | 0.52056 | 0.001312 |
| **YhbS** | Acetyltransferase | 0.521188 | 0.004165 |
| **B4U21_08460** | Lipoprotein | 0.522452 | 0.012866 |
| **YaiW** | DUF1615 domain-containing protein | 0.522457 | 0.000139 |
| **MsrA** | Peptide methionine sulfoxide reductase MsrA | 0.523262 | 0.008547 |
| **PheA** | Bifunctional chorismate mutase/prephenate dehydratase | 0.523497 | 0.000389 |
| **ClpA** | ATP-dependent Clp protease ATP-binding subunit | 0.524047 | 0.000056 |
| **HemE** | Uroporphyrinogen decarboxylase | 0.524128 | 0.000337 |
| **MviM** | Gfo/Idh/MocA family oxidoreductase | 0.524483 | 0.00169 |
| **TyrB_2** | Aminotransferase | 0.52449 | 0.000066 |
| **FxsA** | Membrane protein FxsA | 0.524561 | 0.018865 |
| **OppF** | ABC transporter ATP-binding protein | 0.524854 | 0.000583 |
| **NagK** | N-acetyl-D-glucosamine kinase | 0.525002 | 0.000091 |
| **ClsB** | Cardiolipin synthase B | 0.525196 | 0.004142 |
| **ThiL** | Thiamine-monophosphate kinase | 0.527696 | 0.026257 |
| **AzoR** | FMN-dependent NADH-azoreductase | 0.529252 | 0.000027 |
| **PqiC** | ABC-type uncharacterized transport system, auxiliary component | 0.529851 | 0.007976 |
| **YajO** | Aldo/keto reductase | 0.531068 | 0.002125 |
| **YihD** | DUF1040 family protein | 0.531233 | 0.007527 |
| **EmrB** | Multidrug efflux MFS transporter permease subunit EmrB | 0.53141 | 0.098909 |
| **WabN** | Deacetylase | 0.531626 | 0.104787 |
| **LsrF_2** | Phospho-2-dehydro-3-deoxyheptonate aldolase | 0.532613 | 0.050423 |
| **UbiF_2** | 2-octaprenyl-3-methyl-6-methoxy-1,4-benzoquinol hydroxylase | 0.533257 | 0.000947 |
| **UbiG** | Ubiquinone biosynthesis O-methyltransferase | 0.533464 | 0.000005 |
| **AroA** | 3-phosphoshikimate 1-carboxyvinyltransferase | 0.533807 | 0.000027 |
| **DedA** | DedA family protein | 0.535015 | 0.020086 |
| **SdhA** | Succinate dehydrogenase flavoprotein subunit | 0.536324 | 0.00023 |
| **MutM** | Formamidopyrimidine-DNA glycosylase | 0.536793 | 0.020209 |
| **YgfZ** | tRNA-modifying protein YgfZ | 0.537841 | 0.000227 |
| **BlaSHV-28** | Beta-lactamase | 0.537883 | 0.004009 |
| **RraA** | Regulator of ribonuclease activity A | 0.53806 | 0.001715 |
| **MdoD** | Glucans biosynthesis protein D | 0.538484 | 0.000023 |
| **MutY** | Adenine DNA glycosylase | 0.538591 | 0.005141 |
| **B4U21_32340** | DUF4156 domain-containing protein | 0.539291 | 0.013035 |
| **ManZ_2** | PTS mannose transporter subunit IID | 0.539988 | 0.000852 |
| **Flk** | Flagella biosynthesis regulator | 0.540052 | 0.078276 |
| **KdpB** | Potassium-transporting ATPase ATP-binding subunit | 0.540115 | 0.002606 |
| **DgoD** | D-galactonate dehydratase | 0.540268 | 0.030455 |
| **LsrG** | (4S)-4-hydroxy-5-phosphonooxypentane-2,3-dione isomerase | 0.541379 | 0.09699 |
| **BglY** | Beta-galactosidase | 0.542491 | 0.001568 |
| **ArtJ_2** | ABC transporter arginine-binding protein 1 | 0.543276 | 0.007396 |
| **ETE60_06755** | Sucrose-6-phosphate hydrolase | 0.544196 | 0.113206 |
| **Bfr** | Bacterioferritin | 0.544679 | 0.004195 |
| **FliY_4** | Cystine ABC transporter | 0.545161 | 0.014481 |
| **RssA** | NTE family protein rssA | 0.545258 | 0.067925 |
| **FumA** | Fumarate hydratase class I | 0.545395 | 0.000031 |
| **LolB** | Outer-membrane lipoprotein LolB | 0.545671 | 0.009518 |
| **AcrB** | Efflux pump membrane transporter | 0.54577 | 0.000277 |
| **RimL** | 50S ribosomal protein L7/L12-serine acetyltransferase | 0.545833 | 0.012083 |
| **AmpD** | 1,6-anhydro-N-acetylmuramyl-L-alanine amidase AmpD | 0.547369 | 0.005986 |
| **BglA_1** | 6-phospho-beta-glucosidase | 0.547497 | 0.067984 |
| **B4U21_19045** | Helix-turn-helix domain-containing protein | 0.547689 | 0.185092 |
| **FadL** | Long-chain fatty acid transport protein | 0.547855 | 0.028102 |
| **PdxB** | Erythronate-4-phosphate dehydrogenase | 0.54811 | 0.000059 |
| **YmjA** | DUF2543 domain-containing protein | 0.548124 | 0.000313 |
| **B4U25_31190** | B3/4 domain | 0.548206 | 0.005581 |
| **YhfA** | OsmC family protein | 0.548583 | 0.0003 |
| **SodA** | Superoxide dismutase | 0.548836 | 0.000235 |
| **RpiA** | Ribose-5-phosphate isomerase A | 0.549087 | 0.000399 |
| **Dgt** | Deoxyguanosinetriphosphate triphosphohydrolase | 0.549788 | 0.068795 |
| **BANRA_02439** | DUF3748 domain-containing protein | 0.549815 | 0.000971 |
| **YjgM** | GNAT family N-acetyltransferase | 0.550193 | 0.012717 |
| **GJJ01_09305** | Lipoate--protein ligase family protein | 0.550362 | 0.070877 |
| **Pgl** | 6-phosphogluconolactonase | 0.550533 | 0.000945 |
| **MsrC** | Free methionine-R-sulfoxide reductase | 0.550999 | 0.000016 |
| **YdfG** | Bifunctional NADP-dependent 3-hydroxy acid dehydrogenase/3-hydroxypropionate dehydrogenase YdfG | 0.551126 | 0.000828 |
| **NadE** | NH(3)-dependent NAD(+) synthetase | 0.551189 | 0.000086 |
| **MalH** | 6-phospho-alpha-glucosidase | 0.552394 | 0.08025 |
| **NadD** | Probable nicotinate-nucleotide adenylyltransferase | 0.552882 | 0.00141 |
| **GalP** | Arabinose-proton symporter | 0.553717 | 0.001057 |
| **NudE** | ADP compounds hydrolase nudE | 0.554819 | 0.000604 |
| **YciC** | UPF0259 membrane protein B4U25_11700 | 0.554878 | 0.01396 |
| **Cho** | Cho protein | 0.555708 | 0.044944 |
| **FldC** | (R)-phenyllactyl-CoA dehydratase beta subunit | 0.555771 | 0.001297 |
| **Pgk** | Phosphoglycerate kinase | 0.557159 | 0.000393 |
| **YihI** | Der GTPase-activating protein YihI | 0.558135 | 0.009929 |
| **YegS** | Probable lipid kinase YegS-like | 0.558705 | 0.000142 |
| **FabY** | Acetyltransferase | 0.558731 | 0.027416 |
| **MsyB** | Acidic protein msyB | 0.558794 | 0.036122 |
| **B4U21_24615** | GNAT family N-acetyltransferase | 0.55893 | 0.360454 |
| **HisJ_2** | ABC transporter substrate-binding protein | 0.55905 | 0.0556 |
| **AcrA** | AcrA protein | 0.559624 | 0.000066 |
| **ClpB** | Chaperone protein ClpB | 0.55971 | 0.001193 |
| **GarD_1** | Galactarate dehydratase (L-threo-forming) | 0.560294 | 0.000241 |
| **GlpT** | Glycerol-3-phosphate transporter | 0.561066 | 0.000037 |
| **LysS** | Lysine--tRNA ligase | 0.561127 | 0.000371 |
| **IbpB** | Small heat shock protein IbpB | 0.561348 | 0.023591 |
| **GalS** | DNA-binding transcriptional regulator GalS | 0.56149 | 0.077216 |
| **RlmB** | 23S rRNA (guanosine-2'-O-)-methyltransferase RlmB | 0.561612 | 0.027005 |
| **BANRA_01590** | Uncharacterized protein | 0.561859 | 0.040583 |
| **FusA** | Elongation factor G | 0.561948 | 0.017042 |
| **Dtd** | D-aminoacyl-tRNA deacylase | 0.562907 | 0.00268 |
| **RecD** | RecBCD enzyme subunit RecD | 0.564394 | 0.002161 |
| **GuaC** | GMP reductase | 0.565816 | 0.000058 |
| **ThiQ** | Thiamine import ATP-binding protein ThiQ | 0.565843 | 0.002023 |
| **AscG_1** | AscBF operon repressor | 0.565877 | 0.000313 |
| **LpoB** | Penicillin-binding protein activator LpoB | 0.565912 | 0.0013 |
| **BglH_2** | 6-phospho-beta-glucosidase | 0.565996 | 0.013539 |
| **IspA** | (2E,6E)-farnesyl diphosphate synthase | 0.566413 | 0.03759 |
| **YedJ** | Metal-dependent phosphohydrolase | 0.566555 | 0.000497 |
| **FdhE** | Protein FdhE homolog | 0.566806 | 0.000051 |
| **LacI_1** | Lac operon transcriptional repressor | 0.567017 | 0.000791 |
| **IolE_1** | Putative epimerase/isomerase | 0.567592 | 0.184929 |
| **CpxA** | Histidine kinase | 0.567714 | 0.007175 |
| **SecG** | Protein-export membrane protein SecG | 0.567778 | 0.006381 |
| **ArgH** | Argininosuccinate lyase | 0.567942 | 0.000188 |
| **Fmt** | Methionyl-tRNA formyltransferase | 0.568726 | 0.000158 |
| **Slp** | Outer membrane protein Slp | 0.570591 | 0.002457 |
| **HisP** | Histidine ABC transporter | 0.570651 | 0.005668 |
| **Smg** | Protein Smg | 0.571719 | 0.208561 |
| **YcgE_1** | HTH-type transcriptional regulator ycgE | 0.57264 | 0.000819 |
| **YcjF** | UPF0283 membrane protein F1D54_16305 | 0.572945 | 0.02134 |
| **DapA_2** | Dihydrodipicolinate synthase | 0.573186 | 0.208343 |
| **KdsB** | 3-deoxy-manno-octulosonate cytidylyltransferase | 0.573991 | 0.003589 |
| **YybR_2** | Putative transcriptional regulator | 0.574858 | 0.102618 |
| **YcgM** | 2-keto-4-pentenoate hydratase/2-oxohepta-3-ene-1,7-dioic acid hydratase (Catechol pathway) | 0.575152 | 0.009536 |
| **GreB** | Transcription elongation factor GreB | 0.575808 | 0.034874 |
| **RsmS** | DUF2496 domain-containing protein | 0.575894 | 0.000602 |
| **YegH** | CBS domain-containing protein | 0.576688 | 0.039982 |
| **KsgA** | Ribosomal RNA small subunit methyltransferase A | 0.576895 | 0.000065 |
| **ZipA** | Cell division protein ZipA | 0.577124 | 0.07657 |
| **CorC** | CNNM family magnesium/cobalt transport protein CorC | 0.577482 | 0.000299 |
| **RecF** | DNA replication and repair protein RecF | 0.580158 | 0.08501 |
| **PtsN** | Nitrogen regulatory protein | 0.581031 | 0.015967 |
| **Lon_1** | Lon protease | 0.581933 | 0.00123 |
| **CelA_3** | PTS sugar transporter subunit IIB | 0.583246 | 0.005509 |
| **HemH** | Ferrochelatase | 0.583473 | 0.000817 |
| **PhoB** | Phosphate regulon transcriptional regulatory protein PhoB | 0.584781 | 0.000901 |
| **AroG** | Phospho-2-dehydro-3-deoxyheptonate aldolase | 0.584888 | 0.001342 |
| **RlmC** | 23S rRNA (uracil(747)-C(5))-methyltransferase RlmC | 0.585504 | 0.005732 |
| **YjeR** | Oligoribonuclease | 0.585605 | 0.00058 |
| **MacB** | Macrolide export ATP-binding/permease protein MacB | 0.58588 | 0.082188 |
| **QorB** | NAD(P)H-binding protein | 0.586202 | 0.002041 |
| **GalM** | Aldose 1-epimerase | 0.586714 | 0.000402 |
| **MurQ_1** | Glucokinase regulatory protein | 0.587449 | 0.077392 |
| **UxaC** | Uronate isomerase | 0.588217 | 0.037991 |
| **BANRA_01598** | dTDP-glucose pyrophosphorylase | 0.588386 | 0.210569 |
| **XylB** | Xylulose kinase | 0.589398 | 0.016798 |
| **RffG_1** | SDR family NAD(P)-dependent oxidoreductase | 0.589682 | 0.000215 |
| **Nfi** | Endonuclease V | 0.589939 | 0.006887 |
| **SbcD** | Nuclease SbcCD subunit D | 0.591355 | 0.001138 |
| **FrlB_3** | Phosphosugar isomerase | 0.59219 | 0.361035 |
| **DjlA** | Co-chaperone protein DjlA | 0.592708 | 0.002051 |
| **Crp** | Catabolite activator protein | 0.592766 | 0.000029 |
| **B4U21_12410** | DUF4177 domain-containing protein | 0.592876 | 0.105051 |
| **B4U21_18205** | 4-deoxy-L-threo-5-hexosulose-uronate ketol-isomerase | 0.592981 | 0.007877 |
| **AroF** | Phospho-2-dehydro-3-deoxyheptonate aldolase | 0.593884 | 0.137242 |
| **GshB** | Glutathione synthetase | 0.59389 | 0.000252 |
| **Afr_3** | 1,5-anhydro-D-fructose reductase | 0.593942 | 0.000745 |
| **MgsA** | Methylglyoxal synthase | 0.594737 | 0.130287 |
| **YajR_2** | MFS transporter | 0.594828 | 0.017926 |
| **SlyB** | Outer membrane lipoprotein SlyB | 0.594912 | 0.0168 |
| **MltC** | Membrane-bound lytic murein transglycosylase C | 0.594951 | 0.019518 |
| **Tdk** | Thymidine kinase | 0.595621 | 0.000054 |
| **Tpx** | Thiol peroxidase | 0.596161 | 0.000468 |
| **YbeY** | Endoribonuclease YbeY | 0.597466 | 0.003081 |
| **CcpA_1** | LacI family transcriptional regulator | 0.597667 | 0.227506 |
| **RodA** | Peptidoglycan glycosyltransferase MrdB | 0.598022 | 0.05463 |
| **KstR2_2** | TetR family transcriptional regulator | 0.598234 | 0.270964 |
| **YhdN** | Aldo/keto reductase | 0.598388 | 0.010236 |
| **GlcR_3** | DeoR family transcriptional regulator | 0.599379 | 0.072523 |
| **DegQ** | Periplasmic serine endoprotease DegP-like | 0.599574 | 0.014661 |
| **GJJ01_04245** | Cupin domain-containing protein | 0.600198 | 0.006671 |
| **HtrB** | Lipid A biosynthesis lauroyltransferase | 0.601248 | 0.296474 |
| **AmiC_2** | N-acetylmuramoyl-L-alanine amidase | 0.602247 | 0.000057 |
| **SapB** | Antimicrobial peptide ABC transporter permease SapB | 0.602346 | 0.148184 |
| **YetF_1** | DUF421 domain-containing protein | 0.603087 | 0.104955 |
| **LexA** | LexA repressor | 0.604717 | 0.000435 |
| **NorG** | Aminotransferase class I/II-fold pyridoxal phosphate-dependent enzyme | 0.605219 | 0.250245 |
| **YggX** | Probable Fe(2+)-trafficking protein | 0.605267 | 0.000559 |
| **SeqA** | Negative modulator of initiation of replication | 0.605332 | 0.000518 |
| **PyrC** | Dihydroorotase | 0.606231 | 0.000566 |
| **GJJ01_10080** | MCE family protein | 0.606783 | 0.087057 |
| **RnhA** | Ribonuclease H | 0.607945 | 0.033548 |
| **Pyk** | Pyruvate kinase | 0.607971 | 0.000145 |
| **YdfH** | FCD domain-containing protein | 0.608033 | 0.001398 |
| **YggL** | DUF469 domain-containing protein | 0.608553 | 0.461795 |
| **YfgF_2** | Diguanylate cyclase/cyclic diguanylate phosphodiesterase | 0.609337 | 0.014707 |
| **YgiN** | Antibiotic biosynthesis monooxygenase | 0.609551 | 0.000399 |
| **SlyD** | Peptidyl-prolyl cis-trans isomerase | 0.609817 | 0.000066 |
| **GJJ01_09125** | AsmA2 domain-containing protein | 0.610238 | 0.001622 |
| **UvrB** | UvrABC system protein B | 0.610878 | 0.029962 |
| **GalE** | UDP-glucose 4-epimerase | 0.61094 | 0.000027 |
| **NagZ** | Beta-hexosaminidase | 0.611967 | 0.013219 |
| **YhhX** | Oxidoreductase | 0.612311 | 0.015955 |
| **ExoX** | Exodeoxyribonuclease X | 0.613286 | 0.224197 |
| **CyaY** | Iron-sulfur cluster assembly protein CyaY | 0.61455 | 0.000791 |
| **RbsR_5** | Ribose operon repressor | 0.615348 | 0.052782 |
| **ArgG** | Argininosuccinate synthase | 0.616029 | 0.008471 |
| **GlnP** | GlnP protein | 0.616238 | 0.007726 |
| **YjiA_1** | CobW/P47K family protein | 0.617514 | 0.004895 |
| **QorA_3** | NADPH:quinone reductase | 0.617672 | 0.001729 |
| **AstE_2** | Succinylglutamate desuccinylase | 0.617712 | 0.424883 |
| **PncA** | Bifunctional nicotinamidase/pyrazinamidase | 0.619391 | 0.014007 |
| **CbpA** | Curved DNA-binding protein | 0.620005 | 0.000522 |
| **ETE82_03295** | Succinate-semialdehyde dehydrogenase | 0.620504 | 0.000983 |
| **Psd** | Phosphatidylserine decarboxylase proenzyme | 0.620709 | 0.000016 |
| **ZapD** | Cell division protein ZapD | 0.621235 | 0.002438 |
| **LolA** | Outer-membrane lipoprotein carrier protein | 0.62255 | 0.019763 |
| **MetA** | Homoserine O-succinyltransferase | 0.622705 | 0.267653 |
| **LysC** | Aspartokinase | 0.62318 | 0.000746 |
| **BL124_00032005** | UPF0294 protein BL124_00032005 | 0.623218 | 0.052542 |
| **MzrA** | Modulator protein MzrA | 0.623646 | 0.149938 |
| **NuoG** | NADH-quinone oxidoreductase | 0.623672 | 0.000161 |
| **YcgJ** | Methyltransferase domain-containing protein | 0.624628 | 0.030267 |
| **HslV** | ATP-dependent protease subunit HslV | 0.625358 | 0.043566 |
| **YggS** | Pyridoxal phosphate homeostasis protein | 0.626256 | 0.000033 |
| **GmhA** | Phosphoheptose isomerase | 0.626371 | 0.000486 |
| **PnbA** | Carboxylic ester hydrolase | 0.627262 | 0.003358 |
| **PanE** | 2-dehydropantoate 2-reductase | 0.627458 | 0.08054 |
| **HdfR** | HTH-type transcriptional regulator HdfR | 0.627515 | 0.000132 |
| **HemA** | Glutamyl-tRNA reductase | 0.628165 | 0.071839 |
| **HemD** | Uroporphyrinogen-III synthase | 0.629074 | 0.034272 |
| **RseC** | RseC protein | 0.629462 | 0.497569 |
| **GJJ01_03680** | Putative transport protein F1D54_09695 | 0.629556 | 0.003605 |
| **BL124_00002440** | NAD(P)-dependent oxidoreductase | 0.630724 | 0.000849 |
| **MetH** | Methionine synthase | 0.631718 | 0.000424 |
| **Ugd** | UDP-glucose 6-dehydrogenase | 0.632404 | 0.000225 |
| **SgrR_2** | HTH-type transcriptional regulator SgrR | 0.632959 | 0.002325 |
| **PurM** | Phosphoribosylformylglycinamidine cyclo-ligase | 0.633613 | 0.011224 |
| **YcdY** | Chaperone protein YcdY | 0.634091 | 0.004585 |
| **B4U21_01020** | UPF0231 protein B4U21_01020 | 0.634245 | 0.009683 |
| **SlyA** | Transcriptional regulator SlyA | 0.63637 | 0.00014 |
| **AroC** | Chorismate synthase | 0.636597 | 0.004254 |
| **Mlc_2** | Making large colonies protein | 0.636617 | 0.006356 |
| **SdhB** | Succinate dehydrogenase iron-sulfur subunit | 0.637567 | 0.01227 |
| **UxuA_2** | Mannonate dehydratase | 0.637668 | 0.033028 |
| **MurD** | UDP-N-acetylmuramoylalanine--D-glutamate ligase | 0.638661 | 0.007566 |
| **FeoC** | Probable [Fe-S]-dependent transcriptional repressor | 0.638871 | 0.538889 |
| **BamD** | Outer membrane protein assembly factor BamD | 0.639001 | 0.000656 |
| **LeuC** | 3-isopropylmalate dehydratase large subunit | 0.639186 | 0.001159 |
| **XeaX** | Carnitine monooxygenase reductase subunit | 0.639207 | 0.038872 |
| **Dcm** | Cytosine-specific methyltransferase | 0.639361 | 0.00708 |
| **LeuD** | 3-isopropylmalate dehydratase small subunit | 0.639412 | 0.353398 |
| **LsrR_1** | Cytochrome C biogenesis protein CcdA | 0.639774 | 0.067117 |
| **FbaA** | Fructose-bisphosphate aldolase | 0.640102 | 0.000511 |
| **NarI** | NarI protein | 0.641082 | 0.013131 |
| **B4U21_32995** | 2-dehydro-3-deoxyphosphooctonate aldolase | 0.641134 | 0.022775 |
| **YejG** | YejG protein | 0.641245 | 0.084871 |
| **GltB** | GltB protein | 0.641932 | 0.0002 |
| **RlmJ** | Ribosomal RNA large subunit methyltransferase J | 0.642894 | 0.004239 |
| **TusA_2** | Putative sulfurtransferase YedF | 0.642908 | 0.301132 |
| **EnvZ** | Histidine kinase | 0.642918 | 0.130216 |
| **YhjJ** | Insulinase family protein | 0.643129 | 0.007104 |
| **AnmK** | Anhydro-N-acetylmuramic acid kinase | 0.643563 | 0.015834 |
| **Prc** | Carboxy terminal-processing peptidase | 0.644445 | 0.003052 |
| **NhaR** | LysR family transcriptional regulator | 0.645126 | 0.007844 |
| **PssA** | CDP-diacylglycerol--serine O-phosphatidyltransferase | 0.645386 | 0.005127 |
| **PlsX** | Phosphate acyltransferase | 0.645883 | 0.004909 |
| **YqiB** | DUF1249 domain-containing protein | 0.64689 | 0.091344 |
| **NlhH** | Alpha/beta hydrolase fold domain-containing protein | 0.647776 | 0.107439 |
| **DkgB** | 2,5-didehydrogluconate reductase DkgB | 0.647874 | 0.000052 |
| **RspB** | Putative dehydrogenase, NAD(P)-binding, starvation-sensing protein | 0.64824 | 0.052191 |
| **YicI** | Alpha-xylosidase | 0.648432 | 0.415002 |
| **OsmC** | OsmC family peroxiredoxin | 0.648959 | 0.031352 |
| **PflA** | Pyruvate formate-lyase-activating enzyme | 0.649481 | 0.000192 |
| **MutT** | 7,8-dihydro-8-oxoguanine-triphosphatase | 0.649784 | 0.001404 |
| **GarL** | 5-keto-4-deoxy-D-glucarate aldolase | 0.650062 | 0.043057 |
| **YheO** | Putative regulator | 0.650391 | 0.000217 |
| **CitD2** | Citrate lyase acyl carrier protein | 0.650605 | 0.002643 |
| **XthA** | Exodeoxyribonuclease III | 0.65063 | 0.000024 |
| **FhlA** | FhlA protein | 0.650855 | 0.01793 |
| **ClpP** | ATP-dependent Clp protease proteolytic subunit | 0.65093 | 0.001992 |
| **GbpR** | Galactose-binding protein regulator | 0.652251 | 0.026579 |
| **MgtA** | Magnesium-transporting ATPase, P-type 1 | 0.652333 | 0.079714 |
| **PptA** | Tautomerase PptA | 0.653026 | 0.010977 |
| **Crr** | Crr protein | 0.654351 | 0.000194 |
| **DmlR_21** | HTH-type transcriptional regulator PgrR | 0.654513 | 0.41976 |
| **YcaR** | UPF0434 protein B4U21_07450 | 0.655417 | 0.00117 |
| **YacG** | DNA gyrase inhibitor YacG | 0.655483 | 0.001602 |
| **HosA** | HosA protein | 0.65587 | 0.003668 |
| **Ddl** | D-alanine--D-alanine ligase | 0.656016 | 0.002042 |
| **RsmJ** | Ribosomal RNA small subunit methyltransferase J | 0.656728 | 0.000848 |
| **IlvE** | Branched-chain-amino-acid aminotransferase | 0.657054 | 0.000055 |
| **SerC** | Phosphoserine aminotransferase | 0.658004 | 0.00002 |
| **DeoC2** | Deoxyribose-phosphate aldolase | 0.658344 | 0.0007 |
| **SerB** | O-phosphoserine phosphohydrolase | 0.658465 | 0.000147 |
| **BL124_00018790** | UPF0257 lipoprotein BL124_00018790 | 0.659107 | 0.005342 |
| **DapL_1** | Alanine transaminase | 0.659523 | 0.000405 |
| **DiaA** | DnaA initiator-associating protein DiaA | 0.660135 | 0.001883 |
| **PhnA** | Alkylphosphonate utilization operon protein PhnA | 0.660437 | 0.024225 |
| **FtsE** | Cell division ATP-binding protein FtsE | 0.660547 | 0.000466 |
| **PurN** | Phosphoribosylglycinamide formyltransferase | 0.660806 | 0.001701 |
| **PriC** | Primosomal replication protein N | 0.661614 | 0.334078 |
| **YiiS_1** | DUF406 domain-containing protein | 0.662084 | 0.004106 |
| **Hpt** | Hypoxanthine phosphoribosyltransferase | 0.66211 | 0.043424 |
| **MtfA** | Protein MtfA | 0.662979 | 0.100317 |
| **TldD** | Metalloprotease TldD | 0.663418 | 0.000219 |
| **Kbl** | 2-amino-3-ketobutyrate coenzyme A ligase | 0.663552 | 0.008756 |
| **YhhW_2** | Cupin domain-containing protein | 0.664076 | 0.030313 |
| **YhgF** | 30S ribosomal protein S1 | 0.664182 | 0.000072 |
| **NuoI** | NADH-quinone oxidoreductase subunit I | 0.664355 | 0.050477 |
| **HflC** | Protein HflC | 0.664498 | 0.001525 |
| **YgbJ** | 2-(Hydroxymethyl)glutarate dehydrogenase | 0.665096 | 0.002976 |
| **BANRA_02075** | UPF0270 protein BANRA_02075 | 0.666269 | 0.164532 |
| **NlpE** | Copper homeostasis protein CutF / Lipoprotein NlpE | 0.666657 | 0.002369 |
| **TsaA** | Alkyl hydroperoxide reductase C | 0.667659 | 0.00119 |
| **YrbK** | Lipopolysaccharide export system protein LptC | 0.667695 | 0.221464 |
| **ApbE2** | FAD:protein FMN transferase | 0.668435 | 0.000633 |
| **BssS** | Biofilm formation regulator BssS | 0.668513 | 0.148881 |
| **KefC** | Glutathione-regulated potassium-efflux system protein KefC | 0.668898 | 0.073421 |
| **Cdd** | Cytidine deaminase | 0.669119 | 0.048629 |
| **RibA** | GTP cyclohydrolase-2 | 0.669776 | 0.001038 |
| **MsgA** | DNA damage-inducible protein I | 0.670073 | 0.002002 |
| **PepD** | Aminoacyl-histidine dipeptidase | 0.671253 | 0.003916 |
| **TopB** | DNA topoisomerase 3 | 0.671776 | 0.005772 |
| **FolX** | D-erythro-7,8-dihydroneopterin triphosphate epimerase | 0.672994 | 0.011724 |
| **YceH** | UPF0502 protein B6R99_10675 | 0.673899 | 0.005465 |
| **Lyx_1** | Carbohydrate kinase | 0.674398 | 0.043128 |
| **RuvB** | Holliday junction ATP-dependent DNA helicase RuvB | 0.674493 | 0.00184 |
| **HtpG** | Chaperone protein HtpG | 0.674651 | 0.002636 |
| **DmlR_10** | HTH-type transcriptional regulator DmlR | 0.674993 | 0.001943 |
| **TrpB** | Tryptophan synthase beta chain | 0.676309 | 0.006133 |
| **YdhF** | Aldo/keto reductase | 0.676684 | 0.01879 |
| **FtsI** | Peptidoglycan D,D-transpeptidase FtsI | 0.676707 | 0.01972 |
| **YbfF** | Acyl-CoA esterase | 0.677197 | 0.024813 |
| **BirA** | Bifunctional ligase/repressor BirA | 0.677321 | 0.037165 |
| **YbiP** | Phosphoethanolamine transferase | 0.678067 | 0.187966 |
| **TrxA** | Thioredoxin | 0.678918 | 0.003097 |
| **PurD** | Phosphoribosylamine--glycine ligase | 0.679038 | 0.270438 |
| **GlyA** | Serine hydroxymethyltransferase | 0.67937 | 0.00214 |
| **AcpP** | Acyl carrier protein | 0.679733 | 0.002115 |
| **CpdA_1** | 3',5'-cyclic adenosine monophosphate phosphodiesterase CpdA | 0.680367 | 0.001705 |
| **SbmA** | Microcin B17 transporter | 0.682244 | 0.213543 |
| **YcfH** | Deoxyribonuclease | 0.682274 | 0.001408 |
| **YbdG** | Mechanosensitive ion channel | 0.682371 | 0.01462 |
| **Can** | Carbonic anhydrase | 0.684318 | 0.243035 |
| **GrxA** | GlrX1 protein | 0.684753 | 0.015302 |
| **AcrA_1** | Efflux RND transporter periplasmic adaptor subunit | 0.686601 | 0.025946 |
| **YrbF** | ABC transporter ATP-binding protein | 0.687568 | 0.002289 |
| **YajQ** | UPF0234 protein B4U25_03790 | 0.68845 | 0.001071 |
| **DeoB** | Phosphopentomutase | 0.688474 | 0.000679 |
| **McbR** | GntR family transcriptional regulator | 0.688519 | 0.113116 |
| **GrpE** | Protein GrpE | 0.689116 | 0.001888 |
| **PurC** | Phosphoribosylaminoimidazole-succinocarboxamide synthase | 0.690437 | 0.001081 |
| **AsnC** | AsnC protein | 0.690591 | 0.083056 |
| **SupH** | Cof-type HAD-IIB family hydrolase | 0.690665 | 0.01252 |
| **HisF** | Imidazole glycerol phosphate synthase subunit HisF | 0.690748 | 0.013802 |
| **PtsG_1** | EIICBA-Glc | 0.69104 | 0.00174 |
| **YbiB** | DNA-binding protein YbiB | 0.692072 | 0.002046 |
| **YfhA** | Quorum-sensing regulator protein F | 0.692125 | 0.039229 |
| **GroES** | 10 kDa chaperonin | 0.6927 | 0.001306 |
| **YdaM_2** | Diguanylate cyclase | 0.693322 | 0.210271 |
| **DnaK** | Chaperone protein DnaK | 0.694007 | 0.000817 |
| **YajR_1** | MFS transporter | 0.694201 | 0.003754 |
| **FabR** | HTH-type transcriptional repressor FabR | 0.694733 | 0.075562 |
| **FruR_2** | Catabolite repressor/activator | 0.694927 | 0.001028 |
| **LrpC** | AsnC family transcriptional regulator | 0.695518 | 0.576131 |
| **TrpS** | Tryptophan--tRNA ligase | 0.695673 | 0.000525 |
| **MetQ_1** | Lipoprotein | 0.695726 | 0.009653 |
| **YibT** | YibT protein | 0.695957 | 0.297642 |
| **MurQ** | N-acetylmuramic acid 6-phosphate etherase | 0.696253 | 0.017299 |
| **AroB** | 3-dehydroquinate synthase | 0.696464 | 0.000073 |
| **NuoA** | NADH-quinone oxidoreductase subunit A | 0.69737 | 0.000857 |
| **NuoC** | NADH-quinone oxidoreductase subunit C/D | 0.698144 | 0.000102 |
| **MalG** | MalG protein | 0.698934 | 0.084247 |
| **TehB** | Tellurite resistance methyltransferase TehB | 0.698983 | 0.002932 |
| **YaeQ** | YaeQ family protein | 0.699537 | 0.005129 |
| **GpmA** | 2,3-bisphosphoglycerate-dependent phosphoglycerate mutase | 0.700032 | 0.005497 |
| **Asd** | Aspartate-semialdehyde dehydrogenase | 0.700639 | 0.004262 |
| **FolM** | Dihydromonapterin reductase | 0.700783 | 0.001545 |
| **GlcR_4** | DeoR family transcriptional regulator | 0.701045 | 0.031928 |
| **Mpl** | UDP-N-acetylmuramate--L-alanyl-gamma-D-glutamyl-meso-2,6-diaminoheptandioate ligase | 0.701473 | 0.001119 |
| **FtsW** | Probable peptidoglycan glycosyltransferase FtsW | 0.701721 | 0.008462 |
| **ArgD** | Acetylornithine/succinyldiaminopimelate aminotransferase | 0.703042 | 0.013006 |
| **BaiA1** | Bile acid 7-dehydroxylase 1/3 | 0.703323 | 0.238363 |
| **IolS_3** | Aldo-keto reductase | 0.704229 | 0.121753 |
| **BN49_2482** | Bacterial regulatory proteins, tetR family | 0.704566 | 0.327506 |
| **BepA_1** | Beta-barrel assembly-enhancing protease | 0.704886 | 0.120124 |
| **RamA_2** | (R)-stereoselective amidase | 0.705498 | 0.055366 |
| **ManX** | EIIAB-Man | 0.706511 | 0.001258 |
| **HolB** | DNA polymerase III subunit delta' | 0.707066 | 0.012276 |
| **B4U21_24675** | YCII-related domain | 0.707509 | 0.41737 |
| **Tal** | Transaldolase | 0.708712 | 0.001292 |
| **BglA_1** | 6-phospho-beta-glucosidase | 0.70902 | 0.001372 |
| **YdiI** | 1,4-dihydroxy-2-naphthoyl-CoA hydrolase | 0.709339 | 0.010784 |
| **TrpD** | Anthranilate phosphoribosyltransferase | 0.709682 | 0.027867 |
| **EntA** | 2,3-dihydro-2,3-dihydroxybenzoate dehydrogenase | 0.70997 | 0.010789 |
| **YqjI** | PadR family transcriptional regulator | 0.710328 | 0.028444 |
| **PhoU** | Phosphate-specific transport system accessory protein PhoU | 0.710507 | 0.009319 |
| **Gor** | Glutathione reductase | 0.712191 | 0.000107 |
| **SpeE** | Polyamine aminopropyltransferase | 0.712233 | 0.000493 |
| **Map** | Methionine aminopeptidase | 0.713082 | 0.023949 |
| **HisE** | Histidine biosynthesis bifunctional protein HisIE | 0.71373 | 0.131135 |
| **ModC** | Molybdenum import ATP-binding protein ModC | 0.716724 | 0.131279 |
| **LpxK** | Tetraacyldisaccharide 4'-kinase | 0.716951 | 0.003664 |
| **PrfB** | Peptide chain release factor 2 | 0.717278 | 0.000049 |
| **GreA** | Transcription elongation factor GreA | 0.717773 | 0.000228 |
| **Pad1_2** | Flavin prenyltransferase UbiX | 0.717992 | 0.014497 |
| **GlxK_1** | Glycerate 2-kinase | 0.718002 | 0.103053 |
| **Mfd** | Transcription-repair-coupling factor | 0.718038 | 0.101966 |
| **NadC** | Quinolinate phosphoribosyltransferase [decarboxylating] | 0.720032 | 0.008745 |
| **MltA** | Membrane-bound lytic murein transglycosylase A | 0.720044 | 0.016736 |
| **Pgi** | Glucose-6-phosphate isomerase | 0.720483 | 0.000316 |
| **RsxC** | Ion-translocating oxidoreductase complex subunit C | 0.720643 | 0.024685 |
| **HemC** | Porphobilinogen deaminase | 0.721509 | 0.003307 |
| **YtfP** | AIG2 family protein | 0.722444 | 0.00439 |
| **YjbR_2** | MmcQ/YjbR family DNA-binding protein | 0.725575 | 0.039409 |
| **AcrR_1** | AcrR (Fragment) | 0.726798 | 0.232874 |
| **ZntB** | Zinc transport protein ZntB | 0.728662 | 0.228869 |
| **MoaE2** | MPT synthase subunit 2 | 0.728706 | 0.474336 |
| **MinE** | Cell division topological specificity factor | 0.729472 | 0.00841 |
| **Agp** | 3-phytase | 0.729786 | 0.315437 |
| **B4U21_28540** | DUF3053 domain-containing protein | 0.730128 | 0.012525 |
| **YghU** | Disulfide-bond oxidoreductase YghU | 0.730164 | 0.004852 |
| **AbgR_1** | DNA-binding transcriptional regulator | 0.731393 | 0.450897 |
| **YciA** | Acyl-CoA thioester hydrolase YciA | 0.731895 | 0.089714 |
| **FumB2** | Fumarate hydratase class I | 0.732764 | 0.000449 |
| **DdlA** | D-alanine--D-alanine ligase | 0.733201 | 0.009044 |
| **YrdB** | DUF1488 domain-containing protein | 0.734226 | 0.382832 |
| **GltA_1** | Citrate synthase | 0.734316 | 0.282388 |
| **GlpR_4** | FucR protein | 0.734431 | 0.360858 |
| **C2U49_04825** | GNAT family N-acetyltransferase | 0.735067 | 0.582758 |
| **MurC** | UDP-N-acetylmuramate--L-alanine ligase | 0.735846 | 0.001817 |
| **IhfA** | Integration host factor subunit alpha | 0.736194 | 0.015486 |
| **TruD** | tRNA pseudouridine synthase D | 0.736439 | 0.011727 |
| **GJJ01_23945** | 4-carboxymuconolactone decarboxylase | 0.737159 | 0.037432 |
| **PntB** | NAD(P) transhydrogenase subunit beta | 0.737397 | 0.002834 |
| **HslU** | ATP-dependent protease ATPase subunit HslU | 0.737466 | 0.000314 |
| **Dxr** | 1-deoxy-D-xylulose 5-phosphate reductoisomerase | 0.737906 | 0.009336 |
| **GJJ01_26265** | YbaK/prolyl-tRNA synthetase associated domain-containing protein | 0.737928 | 0.085792 |
| **AlaA** | Alanine transaminase AlaA | 0.737941 | 0.003622 |
| **Tkt** | Transketolase (Fragment) | 0.738128 | 0.001153 |
| **YeiP** | Elongation factor P-like protein | 0.738378 | 0.016085 |
| **QseD** | Cell density-dependent motility repressor | 0.738783 | 0.186817 |
| **WalR_2** | DNA-binding response regulator | 0.739503 | 0.084991 |
| **CitG2** | Probable 2-(5''-triphosphoribosyl)-3'-dephosphocoenzyme-A synthase | 0.739816 | 0.292909 |
| **GJJ01_08160** | Adenylate cyclase | 0.740263 | 0.004172 |
| **PstA** | Phosphate transport system permease protein PstA | 0.741171 | 0.022171 |
| **SpeD** | S-adenosylmethionine decarboxylase proenzyme | 0.741403 | 0.123162 |
| **GJJ01_03295** | MBL fold metallo-hydrolase | 0.741863 | 0.010052 |
| **Epd** | D-erythrose-4-phosphate dehydrogenase | 0.74288 | 0.00239 |
| **Dcd** | dCTP deaminase | 0.74311 | 0.000696 |
| **YfjD** | DUF21 domain-containing protein | 0.743965 | 0.013056 |
| **BamA** | Outer membrane protein assembly factor BamA | 0.744185 | 0.020226 |
| **Tdh** | L-threonine 3-dehydrogenase | 0.744205 | 0.030511 |
| **RpmG** | 50S ribosomal protein L33 | 0.744227 | 0.419425 |
| **GJJ01_11005** | GNAT family N-acetyltransferase | 0.744428 | 0.129095 |
| **AmiA** | N-acetylmuramoyl-L-alanine amidase | 0.7448 | 0.193419 |
| **GlnD** | Bifunctional uridylyltransferase/uridylyl-removing enzyme | 0.745396 | 0.016313 |
| **DnaN** | Beta sliding clamp | 0.746454 | 0.000406 |
| **YhjD** | inner membrane protein YhjD | 0.746457 | 0.011358 |
| **FdnG** | Formate dehydrogenase-N subunit alpha | 0.746838 | 0.184027 |
| **Cof_2** | Cof protein | 0.747001 | 0.001275 |
| **FtsB** | Cell division protein FtsB | 0.747045 | 0.006551 |
| **Ung** | Uracil-DNA glycosylase | 0.747133 | 0.035625 |
| **NarX** | Sensor protein | 0.748243 | 0.450299 |
| **RnpA** | Ribonuclease P protein component | 0.749787 | 0.000071 |
| **YihG** | Acyltransferase | 0.749819 | 0.743978 |
| **KdsA** | 2-dehydro-3-deoxyphosphooctonate aldolase | 0.749845 | 0.000227 |
| **ApaH** | Bis(5'-nucleosyl)-tetraphosphatase, symmetrical | 0.749989 | 0.194878 |
| **LpoA** | Penicillin-binding protein activator LpoA | 0.750683 | 0.001616 |
| **TonB** | Protein TonB | 0.750912 | 0.114462 |
| **PdxK** | Pyridoxal kinase | 0.75343 | 0.357221 |
| **GlpR_8** | DEOR protein | 0.754321 | 0.04263 |
| **C3F39_01005** | Diguanylate cyclase | 0.754445 | 0.140544 |
| **GlpR_6** | DNA-binding transcriptional repressor | 0.754519 | 0.007741 |
| **PlsY** | Glycerol-3-phosphate acyltransferase | 0.75464 | 0.018073 |
| **UvrD** | DNA helicase | 0.754693 | 0.001651 |
| **YhjQ** | Cell division protein | 0.754844 | 0.523655 |
| **MobA** | Molybdenum cofactor guanylyltransferase | 0.755209 | 0.528929 |
| **Mdh** | L-lactate dehydrogenase | 0.755243 | 0.001112 |
| **PanC** | Pantothenate synthetase | 0.755296 | 0.010651 |
| **IlvI_1** | Acetolactate synthase | 0.75608 | 0.066924 |
| **YbgC** | 4-hydroxybenzoyl-CoA thioesterase | 0.756566 | 0.017178 |
| **PdxA** | 4-hydroxythreonine-4-phosphate dehydrogenase | 0.757322 | 0.072454 |
| **DmlR_5** | HTH-type transcriptional regulator DmlR | 0.758087 | 0.000347 |
| **SdiA** | Regulatory activator SdiA | 0.759715 | 0.079871 |
| **LutR_2** | D-Galactonate repressor DgoR | 0.760421 | 0.024617 |
| **icaB** | Polysaccharide deacetylase | 0.760462 | 0.059351 |
| **GJJ01_13115** | CinA-like protein | 0.761322 | 0.065556 |
| **PhoR** | Phosphate regulon sensor protein PhoR | 0.762596 | 0.483451 |
| **YbjS** | 3 beta-hydroxysteroid dehydrogenase/Delta 5-->4-isomerase | 0.762976 | 0.02448 |
| **Ttr** | Acetyltransferase | 0.763011 | 0.030543 |
| **DapA** | 4-hydroxy-tetrahydrodipicolinate synthase | 0.763305 | 0.000058 |
| **RluB** | Pseudouridine synthase | 0.763393 | 0.002921 |
| **NuoB** | NADH-quinone oxidoreductase subunit B | 0.763495 | 0.010136 |
| **YqfB** | N(4)-acetylcytidine amidohydrolase | 0.763994 | 0.000142 |
| **SpoT** | Bifunctional (P)ppGpp synthase/hydrolase SpoT | 0.764195 | 0.036983 |
| **RbsA_5** | Ribose import ATP-binding protein RbsA | 0.764657 | 0.010433 |
| **IspD** | 2-C-methyl-D-erythritol 4-phosphate cytidylyltransferase | 0.764716 | 0.112867 |
| **HpcB** | 3,4-dihydroxyphenylacetate 2,3-dioxygenase | 0.764918 | 0.265907 |
| **RpmE** | 50S ribosomal protein L31 | 0.766174 | 0.084615 |
| **PanB** | 3-methyl-2-oxobutanoate hydroxymethyltransferase | 0.766338 | 0.040197 |
| **GalF** | Alpha-D-glucosyl-1-phosphate uridylyltransferase | 0.767302 | 0.00003 |
| **SpeA** | Biosynthetic arginine decarboxylase | 0.767575 | 0.006459 |
| **WecC** | UDP-N-acetyl-D-mannosamine dehydrogenase | 0.769496 | 0.00272 |
| **PncB** | Nicotinate phosphoribosyltransferase | 0.770924 | 0.060468 |
| **YbhK** | Putative gluconeogenesis factor | 0.771209 | 0.003001 |
| **MiaA** | tRNA dimethylallyltransferase | 0.771585 | 0.002737 |
| **YfeX** | Dyp-type peroxidase | 0.771979 | 0.006521 |
| **MarR** | DNA-binding transcriptional repressor MarR | 0.77333 | 0.178755 |
| **FumD** | Fumarase D | 0.773775 | 0.069168 |
| **TusA** | Sulfur carrier protein TusA | 0.774166 | 0.06528 |
| **BL124_00024950** | O-antigen ligase domain-containing protein | 0.774608 | 0.179691 |
| **BANRA_00238** | Family 10 glycosylhydrolase | 0.775044 | 0.514487 |
| **GluQRS** | Glutamyl-Q tRNA(Asp) synthetase | 0.776534 | 0.098001 |
| **ZapB** | Cell division protein ZapB | 0.777636 | 0.508017 |
| **TesB** | Acyl-CoA thioesterase 2 | 0.778559 | 0.042113 |
| **MipA** | MipA/OmpV family protein | 0.778954 | 0.13795 |
| **TrxB** | Thioredoxin reductase | 0.779701 | 0.02974 |
| **GmhB** | D,D-heptose 1,7-bisphosphate phosphatase | 0.780051 | 0.123251 |
| **SopA** | Plasmid-partitioning protein SopA | 0.780484 | 0.009127 |
| **SuhB_2** | Inositol-1-monophosphatase | 0.780495 | 0.644144 |
| **B4U25_10275** | UPF0227 protein B4U25_10275 | 0.780636 | 0.018654 |
| **Mug** | G/U mismatch-specific DNA glycosylase | 0.780673 | 0.034992 |
| **SspA** | SspA protein | 0.780935 | 0.002413 |
| **YniC_2** | 2-deoxyglucose-6-phosphatase | 0.781755 | 0.002418 |
| **YgfF** | 1,6-dihydroxycyclohexa-2,4-diene-1-carboxylate dehydrogenase | 0.782016 | 0.032346 |
| **HinT** | HIT-like protein hinT | 0.782645 | 0.046668 |
| **Nfo** | Probable endonuclease 4 | 0.785163 | 0.02429 |
| **RarA** | Replication-associated recombination protein A | 0.785238 | 0.059791 |
| **CysH** | Phosphoadenosine phosphosulfate reductase | 0.785599 | 0.115672 |
| **Fur** | Ferric uptake regulation protein | 0.786091 | 0.009922 |
| **ManA** | Mannose-6-phosphate isomerase | 0.7861 | 0.001895 |
| **Tmk** | Thymidylate kinase | 0.786623 | 0.001397 |
| **FabF_1** | 3-oxoacyl-[acyl-carrier-protein] synthase 2 | 0.787907 | 0.034893 |
| **RibD** | Riboflavin biosynthesis protein RibD | 0.788444 | 0.031353 |
| **NrdA** | Ribonucleoside-diphosphate reductase | 0.789055 | 0.005326 |
| **RadA** | DNA repair protein RadA | 0.789537 | 0.041541 |
| **Era** | GTPase Era | 0.790491 | 0.005781 |
| **RelB** | RelB/StbD replicon stabilization protein (Antitoxin to RelE/StbE) | 0.791988 | 0.56305 |
| **RsxG** | Ion-translocating oxidoreductase complex subunit G | 0.79266 | 0.15816 |
| **NarJ** | NarJ protein | 0.793236 | 0.003859 |
| **QuiA** | Glucose dehydrogenase | 0.794529 | 0.000714 |
| **HemX** | HemX protein | 0.794619 | 0.016893 |
| **UgpQ** | Glycerophosphodiester phosphodiesterase | 0.795707 | 0.092197 |
| **DtpA** | Dipeptide and tripeptide permease A | 0.796017 | 0.045749 |
| **B4U21_32230** | Plasmid stability protein | 0.796221 | 0.097399 |
| **AvtA** | Aspartate aminotransferase | 0.796362 | 0.01786 |
| **GlnB** | GlnB protein | 0.796492 | 0.003847 |
| **DhaM** | Phosphoenolpyruvate--glycerone phosphotransferase | 0.796903 | 0.02632 |
| **BioB** | Biotin synthase | 0.796943 | 0.613207 |
| **Gsp_2** | Glutathionylspermidine synthase | 0.797259 | 0.044536 |
| **MdoH** | Glucans biosynthesis glucosyltransferase H | 0.797372 | 0.000939 |
| **GapA** | Glyceraldehyde-3-phosphate dehydrogenase | 0.797501 | 0.034769 |
| **YxeP_1** | Amidohydrolase | 0.798856 | 0.627459 |
| **YcdX** | Probable phosphatase B6R99_10555 | 0.799091 | 0.042393 |
| **FrdC** | Fumarate reductase subunit C | 0.799617 | 0.082947 |
| **OtsB** | Trehalose 6-phosphate phosphatase | 0.799815 | 0.074191 |
| **MdtP_2** | MdtP family multidrug efflux transporter outer membrane subunit | 0.801013 | 0.463254 |
| **GJJ01_17595** | Glycosyltransferase | 0.801209 | 0.087292 |
| **HflX** | GTPase HflX | 0.801338 | 0.002255 |
| **ProP** | Glycine betaine/L-proline transporter ProP | 0.801917 | 0.016579 |
| **PcaB** | 3-carboxy-cis,cis-muconate cycloisomerase | 0.802354 | 0.516687 |
| **WecD** | dTDP-fucosamine acetyltransferase | 0.80283 | 0.38074 |
| **YcgL** | YcgL domain-containing protein B4U21_17620 | 0.803169 | 0.081855 |
| **YifE** | UPF0438 protein YifE | 0.803246 | 0.010649 |
| **AmpG** | AmpG permease | 0.804541 | 0.323351 |
| **MalP_1** | Alpha-1,4 glucan phosphorylase | 0.806204 | 0.062276 |
| **SfsA** | Sugar fermentation stimulation protein homolog | 0.806342 | 0.099976 |
| **Uup** | ATP-binding protein Uup | 0.806531 | 0.094772 |
| **MtlK_2** | D-arabinitol 4-dehydrogenase | 0.80692 | 0.192311 |
| **Zwf** | Glucose-6-phosphate 1-dehydrogenase | 0.810102 | 0.006132 |
| **CsiR** | CsiR, transcriptional repressor of CsiD | 0.810445 | 0.394461 |
| **B4U21_28315** | Protein of uncharacterized function (DUF2629) | 0.811559 | 0.492748 |
| **Fes** | Enterobactin esterase | 0.812108 | 0.130508 |
| **MdtN** | Multidrug transporter subunit MdtN | 0.812326 | 0.05429 |
| **GlmM** | Phosphoglucosamine mutase | 0.812463 | 0.002564 |
| **EmrK_1** | HlyD family efflux transporter periplasmic adaptor subunit | 0.813858 | 0.145274 |
| **NtrC** | DNA-binding transcriptional regulator NtrC | 0.813884 | 0.035081 |
| **NsrR_2** | HTH-type transcriptional repressor NsrR | 0.817223 | 0.008489 |
| **DnaT_1** | Primosomal protein 1 | 0.817558 | 0.282902 |
| **PcaI** | 3-oxoacid CoA-transferase subunit A | 0.818076 | 0.677658 |
| **PpiC_1** | Peptidylprolyl isomerase | 0.818293 | 0.095865 |
| **RecA** | Protein RecA | 0.818753 | 0.040177 |
| **DnaX** | DNA polymerase III subunit gamma/tau | 0.819687 | 0.043463 |
| **AbgR_1** | HTH-type transcriptional regulator AbgR | 0.820723 | 0.014082 |
| **PdhR_1** | DNA-binding transcriptional repressor LldR | 0.821288 | 0.006377 |
| **RsmF** | Ribosomal RNA small subunit methyltransferase F | 0.821296 | 0.003073 |
| **B4U25_30705** | Arylsulfatase | 0.823022 | 0.030593 |
| **DapD** | 2,3,4,5-tetrahydropyridine-2,6-dicarboxylate N-succinyltransferase | 0.824566 | 0.001021 |
| **IspF** | 2-C-methyl-D-erythritol 2,4-cyclodiphosphate synthase | 0.825455 | 0.110162 |
| **YdgJ_2** | Gfo/Idh/MocA family oxidoreductase | 0.825655 | 0.507597 |
| **GloA** | Lactoylglutathione lyase | 0.826548 | 0.000737 |
| **ChbF_1** | 6-phospho-beta-glucosidase | 0.826667 | 0.128775 |
| **ZntR** | HTH-type transcriptional regulator ZntR | 0.826748 | 0.70312 |
| **ElyC** | Envelope biogenesis factor ElyC | 0.828425 | 0.077686 |
| **PepB** | Peptidase B | 0.829102 | 0.1547 |
| **IspB** | Octaprenyl diphosphate synthase | 0.83 | 0.021676 |
| **SanA** | Outer membrane permeability protein SanA | 0.830757 | 0.561472 |
| **RamR** | HTH-type transcriptional repressor Bm3R1 | 0.83143 | 0.41059 |
| **ArgA** | Amino-acid acetyltransferase | 0.831498 | 0.747223 |
| **ThrC** | Threonine synthase | 0.833085 | 0.077157 |
| **TyrA** | T-protein | 0.833572 | 0.002555 |
| **Ppa_2** | Inorganic pyrophosphatase | 0.833984 | 0.013993 |
| **MinD_2** | Iron-sulfur cluster carrier protein | 0.834466 | 0.010707 |
| **CsrA** | Translational regulator CsrA | 0.834532 | 0.487834 |
| **RpmA** | 50S ribosomal protein L27 | 0.835349 | 0.08297 |
| **HemN** | Coproporphyrinogen-III oxidase | 0.835794 | 0.097353 |
| **Hmp** | Flavohemoprotein | 0.835839 | 0.371677 |
| **Rnr** | Ribonuclease R | 0.836009 | 0.004947 |
| **GJJ01_27790** | DUF1722 domain-containing protein | 0.83703 | 0.242057 |
| **InfA** | Translation initiation factor IF-1 | 0.837444 | 0.287856 |
| **PqiB** | Intermembrane transport protein PqiB | 0.837759 | 0.07475 |
| **ThiF_1** | HesA/MoeB/ThiF family protein | 0.837888 | 0.205551 |
| **GlpR_1** | DeoR/GlpR family transcriptional regulator | 0.837953 | 0.004391 |
| **Fdx** | 2Fe-2S ferredoxin | 0.838645 | 0.164538 |
| **LtaE** | Low specificity L-threonine aldolase | 0.838967 | 0.070015 |
| **MaeA** | NAD-dependent malic enzyme | 0.838982 | 0.199489 |
| **DmlR_16** | D-malate degradation protein R | 0.839166 | 0.385344 |
| **PstB** | Phosphate import ATP-binding protein PstB | 0.839707 | 0.076703 |
| **GJJ01_02200** | GNAT family N-acetyltransferase | 0.840651 | 0.653801 |
| **Fre** | Fre protein | 0.84069 | 0.004784 |
| **NadR** | Multifunctional transcriptional regulator/nicotinamide-nucleotide adenylyltransferase/ribosylnicotinamide kinase NadR | 0.841386 | 0.309216 |
| **Rnb** | Exoribonuclease 2 | 0.841697 | 0.037821 |
| **Ppk1** | Polyphosphate kinase | 0.841765 | 0.016681 |
| **ZntA** | Lead, cadmium, zinc and mercury-transporting ATPase | 0.841771 | 0.052523 |
| **Adk** | Adenylate kinase | 0.841949 | 0.016648 |
| **AroH** | Phospho-2-dehydro-3-deoxyheptonate aldolase | 0.842564 | 0.009543 |
| **DmlR_8** | LysR family transcriptional regulator | 0.8429 | 0.699216 |
| **AroE** | Shikimate dehydrogenase (NADP(+)) | 0.84299 | 0.077933 |
| **Udp** | Uridine phosphorylase | 0.843208 | 0.155855 |
| **NudF_1** | ADP-ribose diphosphatase | 0.843931 | 0.03622 |
| **RhmD** | L-rhamnonate dehydratase | 0.844105 | 0.549246 |
| **RimP** | Ribosome maturation factor RimP | 0.844151 | 0.021361 |
| **AdaA_2** | AraC family transcriptional regulator | 0.844309 | 0.144271 |
| **LptE** | LPS-assembly lipoprotein LptE | 0.844422 | 0.106359 |
| **RlmD** | 23S rRNA (uracil(1939)-C(5))-methyltransferase RlmD | 0.844475 | 0.022854 |
| **SufA** | Fe-S cluster assembly scaffold SufA | 0.846215 | 0.385455 |
| **YghZ** | Ion-channel protein | 0.84659 | 0.061845 |
| **Ppx** | Exopolyphosphatase | 0.847378 | 0.051225 |
| **SohB** | Protease SohB | 0.847577 | 0.061613 |
| **FepC** | FepC protein | 0.847794 | 0.387007 |
| **MacA** | MacA protein | 0.847982 | 0.014428 |
| **RsmG** | Ribosomal RNA small subunit methyltransferase G | 0.848331 | 0.082439 |
| **Efp** | Elongation factor P | 0.848362 | 0.010414 |
| **B4U21_26100** | Lipoprotein | 0.849213 | 0.107752 |
| **IolI** | Inosose isomerase | 0.850459 | 0.141233 |
| **YejK** | Nucleoid-associated protein B6R99_06920 | 0.850592 | 0.001906 |
| **MsbB** | Lipid A biosynthesis myristoyltransferase | 0.850656 | 0.04965 |
| **YtfQ** | ABC transporter periplasmic-binding protein YtfQ | 0.850892 | 0.330827 |
| **CyaA** | Adenylate cyclase | 0.851295 | 0.465825 |
| **XerD_1** | Tyrosine recombinase XerD | 0.851556 | 0.270855 |
| **FabH** | 3-oxoacyl-[acyl-carrier-protein] synthase 3 | 0.851638 | 0.111659 |
| **Lnt** | Apolipoprotein N-acyltransferase | 0.85174 | 0.119588 |
| **MalY** | Cystathionine beta-lyase | 0.851936 | 0.12051 |
| **YdfZ** | Putative selenium delivery protein YdfZ | 0.852356 | 0.18859 |
| **TolR** | Tol-Pal system protein TolR | 0.852724 | 0.005859 |
| **TrmJ** | tRNA (cytidine/uridine-2'-O-)-methyltransferase TrmJ | 0.853077 | 0.188869 |
| **MoaA** | GTP 3',8-cyclase | 0.853263 | 0.215064 |
| **GJJ01_14630** | Dicarboxylate transport | 0.853361 | 0.295177 |
| **CmoM** | tRNA 5-carboxymethoxyuridine methyltransferase | 0.85424 | 0.082154 |
| **YrbA** | Acid stress protein IbaG | 0.85471 | 0.126033 |
| **FtsZ** | Cell division protein FtsZ | 0.854991 | 0.023329 |
| **SorC_2** | Helix-turn-helix domain-containing protein | 0.855309 | 0.028112 |
| **IleS** | Isoleucine--tRNA ligase | 0.857596 | 0.056256 |
| **MazG** | Nucleoside triphosphate pyrophosphohydrolase | 0.858044 | 0.105 |
| **B4U21_18600** | Molybdopterin-dependent oxidoreductase | 0.858192 | 0.07147 |
| **RcsD** | Phosphotransferase RcsD | 0.85954 | 0.363008 |
| **PtsJ** | Transcriptional regulator PtsJ | 0.859869 | 0.339193 |
| **FhuA_2** | Ferrioxamine receptor | 0.859877 | 0.455476 |
| **NrdB** | Ribonucleoside-diphosphate reductase | 0.860201 | 0.014005 |
| **TyrS** | Tyrosine--tRNA ligase | 0.860614 | 0.047839 |
| **CspC** | Cold shock protein CspC | 0.860659 | 0.00343 |
| **B4U21_27925** | DUF2500 domain-containing protein | 0.860974 | 0.512755 |
| **RluD** | Pseudouridine synthase | 0.861564 | 0.005986 |
| **MoeA** | Molybdopterin molybdenumtransferase | 0.862213 | 0.069048 |
| **GumN** | Conjugal transfer protein TraB | 0.862332 | 0.65004 |
| **CycB** | Maltodextrin-binding protein | 0.863602 | 0.605739 |
| **FolC** | Dihydrofolate synthase/folylpolyglutamate synthase | 0.863635 | 0.062733 |
| **Add** | Adenosine deaminase | 0.863847 | 0.165691 |
| **IspU** | Ditrans,polycis-undecaprenyl-diphosphate synthase ((2E,6E)-farnesyl-diphosphate specific) | 0.864627 | 0.144851 |
| **LpxC** | UDP-3-O-acyl-N-acetylglucosamine deacetylase | 0.864885 | 0.176307 |
| **YciU** | UPF0263 protein B4U21_16780 | 0.865172 | 0.27711 |
| **Lpp** | Major outer membrane lipoprotein Lpp | 0.865933 | 0.273537 |
| **FhuC_4** | Manganese ABC transporter, ATP-binding protein SitB | 0.866308 | 0.27009 |
| **GldA_2** | Glycerol dehydrogenase | 0.866316 | 0.019903 |
| **YbhC** | Pectinesterase | 0.866318 | 0.201333 |
| **SpeC** | Ornithine decarboxylase | 0.866566 | 0.491164 |
| **UvrA** | UvrABC system protein A | 0.868664 | 0.006128 |
| **ChbG** | Chitooligosaccharide deacetylase | 0.869148 | 0.158703 |
| **FdoG** | FdoG protein | 0.869393 | 0.001575 |
| **YcjX** | Predicted ATPase | 0.869473 | 0.00849 |
| **PrtB** | Oligopeptidase B | 0.87054 | 0.252586 |
| **YvdD** | Cytokinin riboside 5'-monophosphate phosphoribohydrolase | 0.871784 | 0.090413 |
| **B4U21_26220** | UPF0306 protein B4U21_26220 | 0.871806 | 0.211655 |
| **FtsX** | Cell division protein FtsX | 0.872939 | 0.101308 |
| **SerS** | Serine--tRNA ligase | 0.873281 | 0.0004 |
| **RlpA** | Endolytic peptidoglycan transglycosylase RlpA | 0.873595 | 0.053794 |
| **PyrI** | Aspartate carbamoyltransferase regulatory chain | 0.874113 | 0.140875 |
| **BamC** | Outer membrane protein assembly factor BamC | 0.874449 | 0.200109 |
| **YhbJ** | RNase adapter protein RapZ | 0.874774 | 0.323131 |
| **DppB_1** | Dipeptide ABC transporter permease DppB | 0.875181 | 0.304745 |
| **FabD** | Malonyl CoA-acyl carrier protein transacylase | 0.876226 | 0.003478 |
| **TolQ** | Tol-Pal system protein TolQ | 0.876299 | 0.146148 |
| **TreR** | HTH-type transcriptional regulator TreR | 0.876551 | 0.361695 |
| **OprM_1** | Multidrug resistance outer membrane protein MdtP | 0.877158 | 0.458039 |
| **RraB** | Regulator of ribonuclease activity B | 0.87723 | 0.113558 |
| **RuvX** | Putative pre-16S rRNA nuclease | 0.877545 | 0.22393 |
| **YecJ** | DUF2766 domain-containing protein | 0.878582 | 0.290299 |
| **YwaD** | Aminopeptidase | 0.878693 | 0.546953 |
| **SelU** | tRNA 2-selenouridine synthase | 0.878835 | 0.118728 |
| **UppP** | Undecaprenyl-diphosphatase | 0.87935 | 0.484249 |
| **ProA** | Gamma-glutamyl phosphate reductase | 0.88037 | 0.030185 |
| **TamA** | Autotransporter assembly factor TamA | 0.882719 | 0.374361 |
| **YcdB** | Deferrochelatase/peroxidase | 0.882764 | 0.32121 |
| **LplA** | Lipoate-protein ligase A | 0.883216 | 0.386835 |
| **PyrE** | Orotate phosphoribosyltransferase | 0.88327 | 0.159404 |
| **AroP** | AroP protein | 0.883296 | 0.184847 |
| **FabF** | 3-oxoacyl-[acyl-carrier-protein] synthase 2 | 0.883524 | 0.082695 |
| **RoxA_1** | 50S ribosomal protein L16 3-hydroxylase | 0.883662 | 0.325186 |
| **OmpA** | Outer membrane protein A | 0.884621 | 0.218399 |
| **LptG** | LPS export ABC transporter permease LptG | 0.884716 | 0.134777 |
| **Lrp** | Leucine-responsive regulatory protein | 0.885052 | 0.11856 |
| **LysN_1** | 2-aminoadipate transaminase | 0.885143 | 0.328001 |
| **YecC** | Arginine transport ATP-binding protein ArtM | 0.88519 | 0.009314 |
| **MnmA** | tRNA-specific 2-thiouridylase MnmA | 0.885826 | 0.058029 |
| **SdaA** | L-serine dehydratase | 0.886156 | 0.038613 |
| **Cmk** | Cytidylate kinase | 0.887148 | 0.043045 |
| **UbiA** | 4-hydroxybenzoate octaprenyltransferase | 0.887582 | 0.817755 |
| **MutH** | DNA mismatch repair protein MutH | 0.888736 | 0.440005 |
| **PckA** | Phosphoenolpyruvate carboxykinase (ATP) | 0.88945 | 0.090097 |
| **CysS** | Cysteine--tRNA ligase | 0.889528 | 0.228995 |
| **UbiB** | Probable protein kinase UbiB | 0.890081 | 0.480488 |
| **DapE** | Succinyl-diaminopimelate desuccinylase | 0.890094 | 0.141573 |
| **HslR** | Heat shock protein 15 | 0.89054 | 0.755427 |
| **SbcB** | Exodeoxyribonuclease I | 0.892048 | 0.011649 |
| **EngB** | Probable GTP-binding protein EngB | 0.892656 | 0.271239 |
| **LepB** | Signal peptidase I | 0.893099 | 0.160027 |
| **QueF** | NADPH-dependent 7-cyano-7-deazaguanine reductase | 0.8933 | 0.213613 |
| **FolP_2** | Dihydropteroate synthase | 0.894911 | 0.150641 |
| **BANRA_02412** | DUF1198 family protein | 0.895162 | 0.673767 |
| **KatG** | Catalase-peroxidase | 0.895286 | 0.118619 |
| **YeeZ** | NAD(P)-dependent oxidoreductase | 0.895683 | 0.381177 |
| **Pgm** | Alpha-D-glucose phosphate-specific phosphoglucomutase | 0.896014 | 0.754065 |
| **NnrD** | Bifunctional NAD(P)H-hydrate repair enzyme | 0.897061 | 0.618545 |
| **HldD** | ADP-L-glycero-D-manno-heptose-6-epimerase | 0.897455 | 0.011926 |
| **HflK** | Protein HflK | 0.897829 | 0.182253 |
| **KdsD** | Arabinose 5-phosphate isomerase | 0.898566 | 0.114666 |
| **BdhA_2** | D-beta-hydroxybutyrate dehydrogenase | 0.898679 | 0.108049 |
| **DgoA_1** | 2-dehydro-3-deoxy-6-phosphogalactonate aldolase | 0.899006 | 0.811534 |
| **NuoF** | NADH-quinone oxidoreductase subunit F | 0.899105 | 0.109433 |
| **YvoA_2** | 2-aminoethylphosphonate uptake and metabolism regulator | 0.8994 | 0.004831 |
| **UbiD** | 3-octaprenyl-4-hydroxybenzoate carboxy-lyase | 0.899496 | 0.108681 |
| **YtfB** | OapA protein | 0.899617 | 0.638768 |
| **IspH** | 4-hydroxy-3-methylbut-2-enyl diphosphate reductase | 0.900386 | 0.123484 |
| **PucG** | Alanine--glyoxylate aminotransferase family protein | 0.900544 | 0.294395 |
| **B4U21_16400** | Signal peptide protein | 0.900588 | 0.646133 |
| **CarA** | Carbamoyl-phosphate synthase small chain | 0.900612 | 0.065803 |
| **B4U25_40120** | Peptidyl-prolyl cis-trans isomerase | 0.900643 | 0.360738 |
| **RimN** | Threonylcarbamoyl-AMP synthase | 0.900807 | 0.062345 |
| **FbaA_2** | Class II fructose-bisphosphate aldolase | 0.901111 | 0.308233 |
| **SecF** | Protein-export membrane protein SecF | 0.901739 | 0.177434 |
| **SelO** | Protein adenylyltransferase SelO | 0.90188 | 0.524535 |
| **MtlA** | EIICBA-Mtl | 0.9021 | 0.146598 |
| **GJJ01_24345** | MBL fold metallo-hydrolase | 0.90236 | 0.519904 |
| **PdhR_4** | FCD domain-containing protein | 0.902997 | 0.017654 |
| **RpsF** | 30S ribosomal protein S6 | 0.903364 | 0.443161 |
| **BANRA_01257** | 6-deoxy-6-sulfogluconolactonase | 0.903798 | 0.097655 |
| **HldE** | Bifunctional protein HldE | 0.904203 | 0.039912 |
| **PurF** | Amidophosphoribosyltransferase | 0.905136 | 0.135675 |
| **MurE** | UDP-N-acetylmuramoyl-L-alanyl-D-glutamate--2,6-diaminopimelate ligase | 0.906102 | 0.054595 |
| **CvrA** | K(+)/H(+) antiporter NhaP2 | 0.907314 | 0.703819 |
| **B4U21_21150** | Neutral zinc metallopeptidase | 0.907621 | 0.667224 |
| **RcnR** | Metal-sensing transcriptional repressor | 0.907779 | 0.533436 |
| **SorC** | DNA-binding transcriptional regulator | 0.907796 | 0.445352 |
| **DusC** | tRNA-dihydrouridine(16) synthase | 0.907924 | 0.729508 |
| **YidA** | Cof-type HAD-IIB family hydrolase | 0.908563 | 0.241113 |
| **ProC** | Pyrroline-5-carboxylate reductase | 0.909113 | 0.086949 |
| **DamX** | Cell division protein DamX | 0.909156 | 0.387598 |
| **GlpX** | Fructose-1,6-bisphosphatase | 0.910047 | 0.097206 |
| **CbiL** | Cobalt-factor II C(20)-methyltransferase | 0.910376 | 0.58255 |
| **PpnK** | NAD kinase | 0.910851 | 0.367323 |
| **ETE82_00890** | Gluconate 2-dehydrogenase subunit 3 family protein | 0.911111 | 0.54773 |
| **TopA_1** | DNA topoisomerase 1 | 0.912408 | 0.039748 |
| **UreA** | Urease subunit gamma | 0.912674 | 0.732596 |
| **SspB** | ClpXP protease specificity-enhancing factor | 0.912964 | 0.344414 |
| **FadR** | Fatty acid metabolism regulator protein | 0.913039 | 0.001138 |
| **C2U49_03050** | YciK family oxidoreductase | 0.913586 | 0.443567 |
| **BN49_pII0099** | Alpha/beta fold hydrolase | 0.913907 | 0.268855 |
| **MetF** | Methylenetetrahydrofolate reductase | 0.914804 | 0.691882 |
| **Rnc** | Ribonuclease 3 | 0.915568 | 0.270833 |
| **MdlB** | MdlB protein | 0.917437 | 0.366338 |
| **GlnQ** | GlnQ protein | 0.91813 | 0.038223 |
| **RsmD** | Ribosomal RNA small subunit methyltransferase D | 0.918315 | 0.653891 |
| **TolA** | Cell envelope integrity inner membrane protein TolA | 0.918851 | 0.065376 |
| **PtsI** | Phosphoenolpyruvate-protein phosphotransferase | 0.919516 | 0.005903 |
| **HtrB2** | Lipid A biosynthesis lauroyltransferase | 0.919981 | 0.324856 |
| **Cfa_2** | Methyltransferase domain-containing protein | 0.920199 | 0.784922 |
| **BANRA_02721** | DUF1481 domain-containing protein | 0.92027 | 0.855198 |
| **Ssb** | Single-stranded DNA-binding protein | 0.920459 | 0.40037 |
| **B4U25_39220** | Nucleotidyl transferase AbiEii/AbiGii toxin family protein | 0.921355 | 0.779551 |
| **ProY** | ProY protein | 0.921423 | 0.896595 |
| **YbbK** | Protein QmcA | 0.922761 | 0.39807 |
| **AtpF** | ATP synthase subunit b | 0.922904 | 0.07422 |
| **PurT** | Formate-dependent phosphoribosylglycinamide formyltransferase | 0.924146 | 0.124918 |
| **GalU** | UTP--glucose-1-phosphate uridylyltransferase | 0.925453 | 0.023376 |
| **AceA** | Isocitrase | 0.925737 | 0.249629 |
| **CbiC** | CbiC protein | 0.927744 | 0.416942 |
| **GntT** | Gluconate transporter | 0.927799 | 0.677502 |
| **QseG** | Two-component system QseEF-associated lipoprotein QseG | 0.928849 | 0.453859 |
| **HslO** | 33 kDa chaperonin | 0.929831 | 0.01535 |
| **XseA** | Exodeoxyribonuclease 7 large subunit | 0.930421 | 0.569262 |
| **RsgA** | Small ribosomal subunit biogenesis GTPase RsgA | 0.931705 | 0.053384 |
| **TrmA** | tRNA/tmRNA (uracil-C(5))-methyltransferase | 0.931844 | 0.558974 |
| **FtsY** | Signal recognition particle receptor FtsY | 0.932065 | 0.038618 |
| **DnaJ** | Chaperone protein DnaJ | 0.93284 | 0.342553 |
| **DinI_2** | DNA damage-inducible protein I | 0.93284 | 0.914311 |
| **GJJ01_12190** | C-type cytochrome | 0.932965 | 0.623258 |
| **PcaR** | Helix-turn-helix domain-containing protein | 0.933261 | 0.258874 |
| **TsaA** | Putative methyltransferase, YaeB/AF_0241 family | 0.933476 | 0.49491 |
| **SapA** | Antimicrobial peptide ABC transporter substrate-binding protein SapA | 0.933957 | 0.615219 |
| **C3F39_26570** | FAD-binding oxidoreductase | 0.934133 | 0.686346 |
| **Rob** | MDR efflux pump AcrAB transcriptional activator RobA | 0.934398 | 0.013058 |
| **YddE** | Phenazine biosynthesis protein PhzF | 0.934451 | 0.720494 |
| **PdxA2** | 4-hydroxythreonine-4-phosphate dehydrogenase | 0.934632 | 0.106425 |
| **ZapA** | Cell division protein ZapA | 0.934796 | 0.226868 |
| **FadD** | Long-chain-fatty-acid--CoA ligase FadD | 0.935378 | 0.039274 |
| **HrpB** | ATP-dependent helicase HrpB | 0.935861 | 0.294198 |
| **SufC** | ABC transporter ATP-binding protein | 0.937756 | 0.185635 |
| **Tus** | DNA replication terminus site-binding protein | 0.938286 | 0.171576 |
| **Eno** | Enolase | 0.938688 | 0.068138 |
| **AtpB** | ATP synthase subunit a | 0.939939 | 0.722337 |
| **DapF** | Diaminopimelate epimerase | 0.940056 | 0.394481 |
| **B4U25_25590** | DUF2633 domain-containing protein | 0.940164 | 0.708975 |
| **YusV_2** | ATP-binding cassette domain-containing protein | 0.940677 | 0.607974 |
| **MinD** | Cell division inhibitor MinD | 0.9408 | 0.254366 |
| **PlsC** | 1-acyl-sn-glycerol-3-phosphate acyltransferase | 0.941339 | 0.260496 |
| **TrmL** | tRNA (cytidine(34)-2'-O)-methyltransferase | 0.941554 | 0.22626 |
| **PurK** | N5-carboxyaminoimidazole ribonucleotide synthase | 0.943679 | 0.126371 |
| **Map** | Methionine aminopeptidase | 0.944727 | 0.487829 |
| **PpiC_2** | Peptidylprolyl isomerase | 0.944772 | 0.461038 |
| **YdiK** | AI-2E family transporter | 0.944919 | 0.648892 |
| **BhsA_2** | DUF1471 domain-containing protein | 0.945018 | 0.835991 |
| **DkgB_1** | 2,5-diketo-D-gluconic acid reductase B | 0.946549 | 0.28005 |
| **ParE** | DNA topoisomerase 4 subunit B | 0.946589 | 0.213057 |
| **SpeB** | Agmatinase | 0.947423 | 0.270722 |
| **TatB** | Sec-independent protein translocase protein TatB | 0.947866 | 0.785965 |
| **MdoG** | Glucans biosynthesis protein G | 0.949094 | 0.060561 |
| **Ndk** | Nucleoside diphosphate kinase | 0.949095 | 0.235047 |
| **BamB** | Outer membrane protein assembly factor BamB | 0.949741 | 0.581846 |
| **MioC_2** | FMN-binding protein MioC | 0.949911 | 0.515927 |
| **CynR_7** | HTH-type transcriptional regulator CynR | 0.950168 | 0.672324 |
| **FldA** | Flavodoxin | 0.950442 | 0.549968 |
| **FtsL** | Cell division protein FtsL | 0.951897 | 0.307 |
| **HisB** | Histidine biosynthesis bifunctional protein HisB | 0.95243 | 0.157968 |
| **B4U21_09235** | DUF333 domain-containing protein | 0.95285 | 0.654905 |
| **FolE** | GTP cyclohydrolase 1 | 0.953105 | 0.289049 |
| **HolD** | DNA polymerase III subunit psi | 0.95312 | 0.727365 |
| **Pcp** | Pyrrolidone-carboxylate peptidase | 0.953903 | 0.544808 |
| **Por_1** | D-mannonate oxidoreductase | 0.956457 | 0.555015 |
| **ModE** | Molybdenum-dependent transcriptional regulator | 0.957028 | 0.632244 |
| **YbdF** | MmcQ/YjbR family DNA-binding protein | 0.95757 | 0.724293 |
| **CoaE** | Dephospho-CoA kinase | 0.957848 | 0.394989 |
| **RutB_3** | Isochorismatase | 0.958174 | 0.87048 |
| **DcyD** | D-cysteine desulfhydrase | 0.95872 | 0.264433 |
| **UspG_1** | Universal stress protein | 0.96006 | 0.315215 |
| **G5637_19290** | DUF2594 family protein | 0.960086 | 0.725832 |
| **BcsE** | Cellulose biosynthesis protein BcsE | 0.960347 | 0.729081 |
| **MetG** | Methionine--tRNA ligase | 0.960723 | 0.354654 |
| **DapB** | 4-hydroxy-tetrahydrodipicolinate reductase | 0.960755 | 0.653148 |
| **MgtE** | Magnesium transporter MgtE | 0.960869 | 0.834753 |
| **PabB** | Aminodeoxychorismate synthase | 0.961044 | 0.845142 |
| **AtpC** | ATP synthase epsilon chain | 0.96268 | 0.926005 |
| **RluF** | Pseudouridine synthase | 0.962825 | 0.564906 |
| **XylE_1** | 3-methylcatechol 2,3-dioxygenase | 0.963671 | 0.912845 |
| **SrlQ** | Arabinose 5-phosphate isomerase | 0.96478 | 0.10499 |
| **LeuS** | Leucine--tRNA ligase | 0.965014 | 0.269597 |
| **Gsk** | Inosine-guanosine kinase | 0.965526 | 0.818427 |
| **CydD** | Cysteine/glutathione ABC transporter permease/ATP-binding protein CydD | 0.965695 | 0.790154 |
| **HsdS** | Type I restriction endonuclease | 0.966275 | 0.749374 |
| **PanM** | PanD regulatory factor | 0.966453 | 0.711743 |
| **YobD** | UPF0266 membrane protein GJJ01_10160 | 0.966991 | 0.932705 |
| **GspA** | DUF4422 domain-containing protein | 0.967577 | 0.716292 |
| **DsbB** | Disulfide bond formation protein B | 0.967646 | 0.338026 |
| **NifU** | Iron-sulfur cluster assembly scaffold protein IscU | 0.968284 | 0.475041 |
| **YajG** | Lipoprotein | 0.968949 | 0.837219 |
| **YpeA** | Acetyltransferase B4U21_20885 | 0.970215 | 0.693891 |
| **Tal** | Transaldolase | 0.970906 | 0.910798 |
| **PurE** | N5-carboxyaminoimidazole ribonucleotide mutase | 0.972784 | 0.307484 |
| **SutR_4** | Anaerobic benzoate catabolism transcriptional regulator | 0.973374 | 0.577459 |
| **YbeZ** | AAA family ATPase | 0.973421 | 0.497375 |
| **LdhA** | 2-hydroxyacid dehydrogenase | 0.974445 | 0.587979 |
| **RuvA** | Holliday junction ATP-dependent DNA helicase RuvA | 0.974613 | 0.887075 |
| **YneJ** | HTH-type transcriptional regulator gltR | 0.974838 | 0.934197 |
| **LgrD** | NAD-dependent epimerase/dehydratase family protein | 0.975472 | 0.797672 |
| **HscA** | Chaperone protein HscA | 0.976495 | 0.522998 |
| **DinG** | ATP-dependent DNA helicase DinG | 0.976603 | 0.876141 |
| **BetB_2** | NAD/NADP-dependent betaine aldehyde dehydrogenase | 0.978228 | 0.390112 |
| **ThrB** | Homoserine kinase | 0.979279 | 0.923743 |
| **YqaB** | Fructose-1-phosphate phosphatase YqaB | 0.979452 | 0.71242 |
| **PldB** | Lysophospholipase | 0.979559 | 0.932909 |
| **CitX** | Apo-citrate lyase phosphoribosyl-dephospho-CoA transferase | 0.980003 | 0.853595 |
| **PcaD** | 3-oxoadipate enol-lactonase | 0.982071 | 0.597866 |
| **RcsB** | Transcriptional regulatory protein RcsB | 0.98257 | 0.836874 |
| **GroL** | 60 kDa chaperonin | 0.983643 | 0.738075 |
| **HadI** | (R)-2-hydroxyglutaryl-CoA dehydratase activator | 0.983646 | 0.954342 |
| **TreA_2** | Alpha,alpha-phosphotrehalase | 0.984095 | 0.771865 |
| **XylA** | Xylose isomerase | 0.984313 | 0.967302 |
| **GJJ01_13875** | AAA family ATPase | 0.984418 | 0.981306 |
| **YpdB** | DNA-binding response regulator | 0.984426 | 0.826603 |
| **LptB_1** | Lipopolysaccharide export system ATP-binding protein LptB | 0.984768 | 0.788258 |
| **DeoR** | DNA-binding transcriptional repressor DeoR | 0.986585 | 0.853357 |
| **ValS** | Valine--tRNA ligase | 0.988281 | 0.797196 |
| **HolC** | DNA polymerase III subunit chi | 0.988867 | 0.887396 |
| **YhiN** | Aminoacetone oxidase family FAD-binding enzyme | 0.989119 | 0.889801 |
| **UbiF_1** | 2-octaprenyl-3-methyl-6-methoxy-1,4-benzoquinol hydroxylase | 0.990803 | 0.93965 |
| **RbfA** | Ribosome-binding factor A | 0.991377 | 0.732375 |
| **DeoA** | Thymidine phosphorylase | 0.991545 | 0.901432 |
| **GlyQ** | Glycine--tRNA ligase alpha subunit | 0.991714 | 0.937178 |
| **YejL** | UPF0352 protein B4U21_19870 | 0.992076 | 0.94105 |
| **YlaC** | Inner membrane protein | 0.99214 | 0.967821 |
| **RpoA** | DNA-directed RNA polymerase subunit alpha | 0.992489 | 0.91345 |
| **Gsp** | Bifunctional glutathionylspermidine amidase/glutathionylspermidine synthase | 0.992867 | 0.872513 |
| **DD581_08425** | Elongation factor Tu (Fragment) | 0.994998 | 0.927011 |
| **GltX** | Glutamate--tRNA ligase | 0.995073 | 0.849114 |
| **YqjE** | Membrane protein | 0.995233 | 0.861094 |
| **TusD** | Sulfurtransferase TusD | 0.995687 | 0.977198 |
| **PyrD** | Dihydroorotate dehydrogenase (quinone) | 0.996327 | 0.744355 |
| **GlmS** | Glutamine--fructose-6-phosphate aminotransferase [isomerizing] | 0.99695 | 0.948601 |
| **NagA** | N-acetylglucosamine-6-phosphate deacetylase | 0.998492 | 0.979278 |
| **CoaD** | Phosphopantetheine adenylyltransferase | 0.998898 | 0.992513 |
| **Dat** | Dat protein | 1.000074 | 0.997577 |
| **BANRA_04491** | DUF523 domain-containing protein | 1.000937 | 0.995429 |
| **YqgE** | UPF0301 protein B4U21_25090 | 1.001024 | 0.984568 |
| **MukE** | Chromosome partition protein MukE | 1.003108 | 0.90506 |
| **AscG_2** | HTH-type transcriptional regulator AscG | 1.003109 | 0.963685 |
| **NagD** | HAD-IIA family hydrolase | 1.003328 | 0.956825 |
| **FabA** | 3-hydroxydecanoyl-[acyl-carrier-protein] dehydratase | 1.003551 | 0.931208 |
| **HemL** | Glutamate-1-semialdehyde 2,1-aminomutase | 1.004455 | 0.879261 |
| **GJJ01_01625** | Outer membrane protein | 1.004736 | 0.981786 |
| **IciA** | HTH-type transcriptional regulator ArgP | 1.005172 | 0.936304 |
| **HupA** | DNA-binding protein | 1.005559 | 0.936133 |
| **IgaA** | IgaA | 1.005804 | 0.976884 |
| **ThyA** | Thymidylate synthase | 1.006222 | 0.956513 |
| **B4U25_10290** | Glycine zipper 2TM domain-containing protein | 1.006626 | 0.986458 |
| **LolE** | Lipoprotein transporter subunit LolE | 1.007705 | 0.949982 |
| **YecM** | Protein yecM | 1.008032 | 0.959978 |
| **KdbD** | Histidine kinase | 1.008459 | 0.983589 |
| **YrbD** | ABC transporter periplasmic substrate-binding protein | 1.008937 | 0.868881 |
| **AidB** | Isovaleryl-CoA dehydrogenase | 1.010774 | 0.921045 |
| **Cls** | Cardiolipin synthase A | 1.011139 | 0.921998 |
| **RibF** | Riboflavin biosynthesis protein | 1.01139 | 0.899148 |
| **Rep** | ATP-dependent DNA helicase Rep | 1.012693 | 0.858535 |
| **Ompk17** | Outer membrane protein X | 1.013876 | 0.971424 |
| **AglB** | 6-phospho-alpha-glucosidase | 1.013954 | 0.911158 |
| **UbiE** | Ubiquinone/menaquinone biosynthesis C-methyltransferase UbiE | 1.014827 | 0.749703 |
| **DeoD** | Purine nucleoside phosphorylase DeoD-type | 1.015467 | 0.904124 |
| **AmpE** | AmpE protein | 1.01552 | 0.953103 |
| **YbiS** | L,D-transpeptidase | 1.015992 | 0.905813 |
| **YebO** | Uncharacterized protein YebO | 1.016838 | 0.86731 |
| **ProS_2** | Cys-tRNA(Pro)/cys-tRNA(Cys) deacylase | 1.016968 | 0.922435 |
| **Rng** | Ribonuclease G | 1.017356 | 0.751211 |
| **Fnr** | FNR family transcription factor | 1.017791 | 0.835577 |
| **CsdA** | Cysteine desulfurase CsdA | 1.017971 | 0.787392 |
| **MviM_1** | Oxidoreductase | 1.018545 | 0.877577 |
| **YgfX** | Inner membrane protein | 1.018647 | 0.985262 |
| **Ppc** | Phosphoenolpyruvate carboxylase | 1.018687 | 0.554418 |
| **CueR** | Copper efflux regulator | 1.021377 | 0.748638 |
| **IlvY** | DNA-binding transcriptional regulator IlvY | 1.021432 | 0.854339 |
| **TruA** | tRNA pseudouridine synthase A | 1.02272 | 0.154706 |
| **Asd2** | Aspartate-semialdehyde dehydrogenase | 1.024934 | 0.890874 |
| **ArgB** | Acetylglutamate kinase | 1.025753 | 0.420817 |
| **Rne** | Ribonuclease E | 1.026359 | 0.59786 |
| **CorA** | Magnesium transport protein CorA | 1.026532 | 0.299934 |
| **Hha_1** | Haemolysin expression modulating protein | 1.027439 | 0.909142 |
| **CspA** | Cold shock protein CspA | 1.027777 | 0.537736 |
| **FabG_9** | 3-oxoacyl-[acyl-carrier-protein] reductase | 1.028045 | 0.790572 |
| **Lrp_4** | AsnC family transcriptional regulator | 1.028616 | 0.905892 |
| **PpiD** | Peptidylprolyl isomerase | 1.028824 | 0.518625 |
| **B4U25_24840** | Putative DNA-binding transcriptional regulator | 1.029093 | 0.918521 |
| **HflB_1** | ATP-dependent zinc metalloprotease FtsH | 1.029796 | 0.508839 |
| **RhlB** | ATP-dependent RNA helicase RhlB | 1.030685 | 0.471986 |
| **MukB** | Chromosome partition protein MukB | 1.030741 | 0.544787 |
| **ThiE** | Thiamine-phosphate synthase | 1.031218 | 0.952659 |
| **RsmH** | Ribosomal RNA small subunit methyltransferase H | 1.031377 | 0.476863 |
| **AllS_5** | HTH-type transcriptional activator AllS | 1.031914 | 0.6988 |
| **OxaA** | Membrane protein insertase YidC | 1.031935 | 0.530368 |
| **IspG** | 4-hydroxy-3-methylbut-2-en-1-yl diphosphate synthase (flavodoxin) | 1.032001 | 0.437736 |
| **ClpX** | ATP-dependent Clp protease ATP-binding subunit ClpX | 1.034008 | 0.503244 |
| **MetB** | Cystathionine gamma-synthase | 1.034097 | 0.681996 |
| **PurA** | Adenylosuccinate synthetase | 1.034131 | 0.601473 |
| **FhuD** | Fe(3+)-hydroxamate ABC transporter substrate-binding protein FhuD | 1.034153 | 0.842701 |
| **MinC** | Probable septum site-determining protein MinC | 1.03461 | 0.702525 |
| **GrxD** | Glutaredoxin | 1.035166 | 0.42676 |
| **RpoD** | RNA polymerase sigma factor RpoD | 1.036189 | 0.450324 |
| **B4U21_18290** | Lipoprotein | 1.036624 | 0.79274 |
| **AlaS** | Alanine--tRNA ligase | 1.037522 | 0.076603 |
| **PyrF** | Orotidine 5'-phosphate decarboxylase | 1.037577 | 0.649227 |
| **Hns_1** | DNA-binding protein | 1.037718 | 0.483508 |
| **DmlR_10** | LysR family transcriptional regulator | 1.038082 | 0.738829 |
| **MsbA_1** | ATP-dependent lipid A-core flippase | 1.038121 | 0.429955 |
| **YhdH** | Acryloyl-CoA reductase | 1.038166 | 0.927461 |
| **YtfJ** | Protein ytfJ | 1.038412 | 0.628598 |
| **DcuR** | Transcriptional regulatory protein | 1.03901 | 0.584747 |
| **RdgB** | dITP/XTP pyrophosphatase | 1.039513 | 0.630769 |
| **PurB** | Adenylosuccinate lyase | 1.039942 | 0.029148 |
| **MalQ** | 4-alpha-glucanotransferase | 1.041192 | 0.917268 |
| **GanB** | Arabinogalactan endo-beta-1,4-galactanase | 1.041697 | 0.916666 |
| **YbaL** | Kef family K(+) transporter | 1.041855 | 0.746978 |
| **RplY** | 50S ribosomal protein L25 | 1.043398 | 0.026802 |
| **Apt** | Adenine phosphoribosyltransferase | 1.043649 | 0.25547 |
| **BetU** | BCCT family transporter | 1.044978 | 0.584952 |
| **LolC** | Lipoprotein-releasing ABC transporter permease subunit LolC | 1.045048 | 0.529403 |
| **BANRA_01242** | tRNA-uridine aminocarboxypropyltransferase | 1.045085 | 0.529079 |
| **PurR** | HTH-type transcriptional repressor PurR | 1.045786 | 0.315524 |
| **LysS** | Lysine--tRNA ligase | 1.046575 | 0.265145 |
| **PrfA** | Peptide chain release factor 1 | 1.047578 | 0.618429 |
| **OmpK36** | OmpK36 | 1.04767 | 0.723667 |
| **TypA** | GTP-binding protein | 1.048588 | 0.456188 |
| **DadB** | Alanine racemase | 1.049057 | 0.694668 |
| **YcfL** | DUF1425 domain-containing protein | 1.04909 | 0.873473 |
| **CysB** | Cys regulon transcriptional activator | 1.049522 | 0.204885 |
| **MtnN** | 5'-methylthioadenosine/S-adenosylhomocysteine nucleosidase | 1.04955 | 0.318904 |
| **MutS** | DNA mismatch repair protein MutS | 1.049695 | 0.708291 |
| **DrrA** | ABC transporter ATP-binding protein | 1.049996 | 0.423172 |
| **AnsP** | AnsP protein | 1.051191 | 0.758546 |
| **GlnS** | Glutamine--tRNA ligase | 1.05242 | 0.133784 |
| **Gph** | Phosphoglycolate phosphatase | 1.052642 | 0.714657 |
| **YghB** | DedA family inner membrane protein YghB | 1.052829 | 0.661823 |
| **LigA** | DNA ligase | 1.055265 | 0.340233 |
| **MdeA** | DgaE family pyridoxal phosphate-dependent ammonia lyase | 1.055293 | 0.54234 |
| **B4U21_01060** | YacC family pilotin-like protein | 1.055728 | 0.743371 |
| **MltB** | Lytic murein transglycosylase B | 1.056343 | 0.610324 |
| **YceG** | Endolytic murein transglycosylase | 1.058448 | 0.628242 |
| **UspG2** | Universal stress protein | 1.058644 | 0.936107 |
| **Dut** | Deoxyuridine 5'-triphosphate nucleotidohydrolase | 1.059653 | 0.270332 |
| **HisS** | Histidine--tRNA ligase | 1.060088 | 0.254485 |
| **YpdA** | Histidine kinase | 1.060162 | 0.928667 |
| **YhbV** | Ubiquinone biosynthesis protein UbiV | 1.06017 | 0.848307 |
| **BetA** | Oxygen-dependent choline dehydrogenase | 1.061452 | 0.439902 |
| **RlmI** | Ribosomal RNA large subunit methyltransferase I | 1.061823 | 0.564691 |
| **Alr** | Alanine racemase | 1.062269 | 0.672449 |
| **YhdE** | dTTP/UTP pyrophosphatase | 1.062796 | 0.52096 |
| **Frr** | Ribosome-recycling factor | 1.062798 | 0.070814 |
| **YigA** | DUF484 domain-containing protein | 1.063806 | 0.610872 |
| **PanD** | Aspartate 1-decarboxylase | 1.063813 | 0.610292 |
| **LolD_2** | Lipoprotein-releasing system ATP-binding protein LolD | 1.064184 | 0.494087 |
| **Gnd** | 6-phosphogluconate dehydrogenase, decarboxylating | 1.064479 | 0.207432 |
| **UvrC** | UvrABC system protein C | 1.065285 | 0.278983 |
| **FolD** | Bifunctional protein FolD | 1.065526 | 0.078703 |
| **GntK** | Gluconokinase | 1.065649 | 0.09714 |
| **MutL** | DNA mismatch repair protein MutL | 1.066164 | 0.535958 |
| **RlmE** | Ribosomal RNA large subunit methyltransferase E | 1.067344 | 0.197368 |
| **RodZ** | Cytoskeleton protein RodZ | 1.067827 | 0.507974 |
| **BioH** | Pimeloyl-[acyl-carrier protein] methyl ester esterase | 1.068873 | 0.344488 |
| **YjiY** | Carbon starvation protein | 1.068948 | 0.624211 |
| **LapB** | Lipopolysaccharide assembly protein B | 1.069769 | 0.733924 |
| **YajC** | Sec translocon accessory complex subunit YajC | 1.07067 | 0.398926 |
| **FruB_2** | EIII-Fru | 1.070756 | 0.811003 |
| **YpfH** | Esterase | 1.071301 | 0.4503 |
| **ArgS** | Arginine--tRNA ligase | 1.071471 | 0.329511 |
| **SlpA** | Peptidyl-prolyl cis-trans isomerase | 1.071742 | 0.755255 |
| **LacZ** | Beta-galactosidase | 1.071786 | 0.36277 |
| **HemB** | Delta-aminolevulinic acid dehydratase | 1.072493 | 0.184344 |
| **YjiA_2** | GTP-binding protein | 1.072511 | 0.250946 |
| **AcpH** | Acyl carrier protein phosphodiesterase | 1.072841 | 0.642645 |
| **RluE** | Pseudouridine synthase | 1.073259 | 0.763359 |
| **FabG_2** | 3-oxoacyl-ACP reductase | 1.073487 | 0.783984 |
| **SapC** | Antimicrobial peptide ABC transporter permease SapC | 1.073727 | 0.794347 |
| **SpeG_1** | GNAT family N-acetyltransferase | 1.073951 | 0.863559 |
| **RpsU** | 30S ribosomal protein S21 | 1.074627 | 0.478481 |
| **B4U25_14410** | ROK family protein | 1.075314 | 0.754124 |
| **GlpE** | Thiosulfate sulfurtransferase GlpE | 1.076485 | 0.359306 |
| **DegS** | Peptidase Do | 1.077558 | 0.614999 |
| **GutB_1** | Galactitol-1-phosphate 5-dehydrogenase | 1.078171 | 0.808588 |
| **FabG** | 3-oxoacyl-[acyl-carrier-protein] reductase | 1.07831 | 0.046953 |
| **RibB** | 3,4-dihydroxy-2-butanone 4-phosphate synthase | 1.078519 | 0.355811 |
| **SlmA** | Nucleoid occlusion factor SlmA | 1.078636 | 0.434366 |
| **FruA** | PTS fructose transporter subunit IIBC | 1.08044 | 0.586224 |
| **MetJ** | Met repressor | 1.080579 | 0.306542 |
| **NadA** | Quinolinate synthase A | 1.08171 | 0.673578 |
| **MalI** | Degradation activator | 1.082763 | 0.588994 |
| **HflD** | High frequency lysogenization protein HflD homolog | 1.083077 | 0.469118 |
| **NqrF** | Na(+)-translocating NADH-quinone reductase subunit F | 1.083114 | 0.524252 |
| **SopB** | ParB/RepB/Spo0J family plasmid partition protein | 1.083128 | 0.457689 |
| **ParM** | PRTRC system protein D | 1.083327 | 0.085251 |
| **YjjK** | Energy-dependent translational throttle protein EttA | 1.08355 | 0.010773 |
| **AccC1** | Biotin carboxylase | 1.083883 | 0.087562 |
| **QueC** | 7-cyano-7-deazaguanine synthase | 1.084023 | 0.204066 |
| **PurH** | Bifunctional purine biosynthesis protein PurH | 1.084115 | 0.124542 |
| **PorA** | Pyruvate-flavodoxin oxidoreductase | 1.084993 | 0.344318 |
| **Ddc** | Aspartate aminotransferase family protein | 1.085225 | 0.092028 |
| **AhcY** | Adenosylhomocysteinase | 1.085326 | 0.321855 |
| **Gmk** | Guanylate kinase | 1.086492 | 0.048731 |
| **YdgJ_1** | Oxidoreductase | 1.086958 | 0.205253 |
| **GlgB** | 1,4-alpha-glucan branching enzyme GlgB | 1.086996 | 0.392979 |
| **RecG** | ATP-dependent DNA helicase RecG | 1.087438 | 0.530453 |
| **DD581_13285** | UPF0597 protein C7V41_21510 | 1.087555 | 0.411522 |
| **KdgR_1** | DNA-binding transcriptional regulator KdgR | 1.08785 | 0.070769 |
| **ThiM** | Hydroxyethylthiazole kinase | 1.088026 | 0.602276 |
| **RcsF** | Outer membrane lipoprotein RcsF | 1.089697 | 0.491416 |
| **CspE** | Cold shock-like protein CspE | 1.089827 | 0.426391 |
| **CysN** | Sulfate adenylyltransferase subunit 1 | 1.090885 | 0.541192 |
| **YedI** | DUF808 domain-containing protein | 1.090959 | 0.438948 |
| **IlvD** | Dihydroxy-acid dehydratase | 1.092952 | 0.068121 |
| **Wzc** | Inner membrane tyrosine autokinase | 1.094217 | 0.448594 |
| **PriB** | Primosomal replication protein N | 1.096478 | 0.723719 |
| **BANRA_03325** | Permease | 1.096614 | 0.767976 |
| **MdcR** | HTH-type transcriptional regulator gltC | 1.097433 | 0.219979 |
| **PntA** | NAD(P) transhydrogenase subunit alpha | 1.098288 | 0.069221 |
| **AroK_1** | Adenylate kinase | 1.098864 | 0.641008 |
| **FrdA** | Fumarate reductase flavoprotein subunit | 1.099041 | 0.136797 |
| **SuhB_1** | Inositol-1-monophosphatase | 1.101024 | 0.080117 |
| **VapC** | Ribonuclease VapC | 1.1013 | 0.260171 |
| **CobT** | Nicotinate-nucleotide--dimethylbenzimidazole phosphoribosyltransferase | 1.102418 | 0.208108 |
| **GltD** | FAD-dependent oxidoreductase | 1.104349 | 0.244673 |
| **EntF** | Enterobactin non-ribosomal peptide synthetase EntF | 1.104544 | 0.184336 |
| **B4U21_09740** | YfeABCD regulator yfeE | 1.105073 | 0.742417 |
| **LepA** | Elongation factor 4 | 1.105663 | 0.049112 |
| **Pcm** | Protein-L-isoaspartate O-methyltransferase | 1.107979 | 0.534196 |
| **RdgC** | Recombination-associated protein RdgC | 1.1081 | 0.044017 |
| **GJJ01_15935** | tRNA/rRNA methyltransferase | 1.108309 | 0.210958 |
| **YbbH_1** | (Fe-S)-cluster assembly protein | 1.108445 | 0.269703 |
| **IscX** | Protein IscX | 1.108596 | 0.149665 |
| **NrdR** | Transcriptional repressor NrdR | 1.108613 | 0.158449 |
| **SurE** | 5'/3'-nucleotidase SurE | 1.109161 | 0.28253 |
| **PgeF** | Purine nucleoside phosphorylase | 1.111135 | 0.247441 |
| **Tsf** | Elongation factor Ts | 1.111858 | 0.000189 |
| **B4U21_26905** | GNAT family N-acetyltransferase | 1.113562 | 0.641389 |
| **MdaB** | Flavodoxin family protein | 1.114776 | 0.179915 |
| **SecB** | Protein-export protein SecB | 1.115008 | 0.024778 |
| **YebC** | Probable transcriptional regulatory protein B6R99_02425 | 1.115304 | 0.052479 |
| **NrdE** | Ribonucleoside-diphosphate reductase | 1.115553 | 0.206138 |
| **CysI** | Sulfite reductase [NADPH] hemoprotein beta-component | 1.115929 | 0.133819 |
| **ZapC** | Cell division protein ZapC | 1.117731 | 0.195142 |
| **MreC** | Cell shape-determining protein MreC | 1.121599 | 0.039393 |
| **MetK** | S-adenosylmethionine synthase | 1.122629 | 0.032434 |
| **TyrB_1** | Aminotransferase | 1.123046 | 0.737981 |
| **PheS** | Phenylalanine--tRNA ligase alpha subunit | 1.124915 | 0.018348 |
| **YaeL** | Zinc metalloprotease | 1.126747 | 0.179941 |
| **AsmA** | AsmA protein | 1.127766 | 0.182405 |
| **FolK** | 2-amino-4-hydroxy-6-hydroxymethyldihydropteridine pyrophosphokinase | 1.12797 | 0.374579 |
| **RbsR_2** | 2-ketogluconate utilization repressor PtxS | 1.129003 | 0.836735 |
| **RadD_2** | ATP-dependent helicase | 1.129902 | 0.035432 |
| **YhbY** | RNA-binding protein | 1.131469 | 0.247084 |
| **SixA** | Phosphohistidine phosphatase SixA | 1.13213 | 0.107005 |
| **GlpQ_1** | Glycerophosphodiester phosphodiesterase | 1.132337 | 0.674298 |
| **LpxA** | Acyl-[acyl-carrier-protein]--UDP-N-acetylglucosamine O-acyltransferase | 1.132538 | 0.168241 |
| **DnaA** | Chromosomal replication initiator protein DnaA | 1.134441 | 0.146958 |
| **EngA** | GTPase Der | 1.136653 | 0.043357 |
| **SmpB** | SsrA-binding protein | 1.136777 | 0.028827 |
| **DnaQ** | DNA polymerase III subunit epsilon | 1.137451 | 0.59213 |
| **MurG** | UDP-N-acetylglucosamine--N-acetylmuramyl-(pentapeptide) pyrophosphoryl-undecaprenol N-acetylglucosamine transferase | 1.137639 | 0.10761 |
| **PaaZ** | Bifunctional aldehyde dehydrogenase/enoyl-CoA hydratase | 1.137847 | 0.652733 |
| **GpsA** | Glycerol-3-phosphate dehydrogenase [NAD(P)+] | 1.141356 | 0.059832 |
| **NqrA** | Na(+)-translocating NADH-quinone reductase subunit A | 1.142389 | 0.303321 |
| **YfaU** | 2-keto-3-deoxy-L-rhamnonate aldolase | 1.143936 | 0.760564 |
| **SufB** | Fe-S cluster assembly protein SufB | 1.144371 | 0.131538 |
| **PmbA** | Metalloprotease PmbA | 1.145321 | 0.015005 |
| **Tig** | Trigger factor | 1.145954 | 0.003604 |
| **RpsM** | 30S ribosomal protein S13 | 1.146519 | 0.107291 |
| **OxyR_1** | LysR family regulatory protein CidR | 1.149208 | 0.517411 |
| **YheS_2** | ABC transporter ATP-binding protein | 1.1512 | 0.025708 |
| **RpoC** | DNA-directed RNA polymerase subunit beta' | 1.15187 | 0.000283 |
| **YcjG** | Dipeptide epimerase | 1.153984 | 0.380301 |
| **Aas** | Bifunctional protein Aas | 1.154408 | 0.414412 |
| **GalT** | Galactose-1-phosphate uridylyltransferase | 1.15467 | 0.762777 |
| **BtuD** | Vitamin B12 import ATP-binding protein BtuD | 1.155622 | 0.179063 |
| **BglA_3** | Family 1 glycosylhydrolase | 1.158003 | 0.260668 |
| **GlmU** | Bifunctional protein GlmU | 1.158685 | 0.162062 |
| **YiiS_1** | DUF406 domain-containing protein | 1.159498 | 0.019697 |
| **Usg** | Aspartate-semialdehyde dehydrogenase | 1.161828 | 0.010608 |
| **SbcC** | Nuclease SbcCD subunit C | 1.161943 | 0.060641 |
| **MurB** | UDP-N-acetylenolpyruvoylglucosamine reductase | 1.163038 | 0.119829 |
| **HisD** | Histidinol dehydrogenase | 1.163183 | 0.756102 |
| **GyrA** | DNA gyrase subunit A | 1.163223 | 0.02926 |
| **B4U21_17610** | UPF0260 protein B4U21_17610 | 1.163397 | 0.221793 |
| **RpoN** | RNA polymerase sigma-54 factor | 1.163839 | 0.067996 |
| **Eda** | 2-dehydro-3-deoxy-phosphogluconate aldolase | 1.164128 | 0.068381 |
| **AllS_4** | LysR family transcriptional regulator | 1.16429 | 0.509296 |
| **Ndh** | FAD-dependent oxidoreductase | 1.16448 | 0.02447 |
| **Mog** | Mog protein | 1.168419 | 0.054948 |
| **YibL** | Protein of uncharacterized function (DUF2810) | 1.169793 | 0.254175 |
| **NudK** | GDP-mannose pyrophosphatase nudK | 1.169979 | 0.090577 |
| **YeaP** | Diguanylate cyclase | 1.170278 | 0.189411 |
| **PatD** | Gamma-aminobutyraldehyde dehydrogenase | 1.171313 | 0.314445 |
| **FrmR** | Metal-sensing transcriptional repressor | 1.17448 | 0.048401 |
| **YehT** | DNA-binding response regulator | 1.176325 | 0.15637 |
| **CatB** | CatB protein | 1.176616 | 0.558168 |
| **IclR** | Acetate operon repressor | 1.177976 | 0.038287 |
| **UvrY** | BarA-associated response regulator UvrY (GacA, SirA) | 1.178653 | 0.466322 |
| **CbiK** | Sirohydrochlorin cobaltochelatase | 1.178913 | 0.043595 |
| **PtsG_5** | EIICB-Glc | 1.179156 | 0.079818 |
| **IscS** | Cysteine desulfurase IscS | 1.179315 | 0.019469 |
| **LutR_1** | FCD domain-containing protein | 1.180032 | 0.271564 |
| **Tgt** | Queuine tRNA-ribosyltransferase | 1.180084 | 0.01689 |
| **KstR2_1** | HTH-type transcriptional repressor KstR2 | 1.180483 | 0.329925 |
| **DnaB** | Replicative DNA helicase | 1.180661 | 0.001611 |
| **PriM** | DNA primase | 1.181016 | 0.108671 |
| **Mtr_1** | Tryptophan permease | 1.182914 | 0.447293 |
| **YeaZ** | t(6)A37 threonylcarbamoyladenosine biosynthesis protein TsaB | 1.1846 | 0.121196 |
| **AspS** | Aspartate--tRNA ligase | 1.184824 | 0.007991 |
| **CysE** | Serine acetyltransferase | 1.188078 | 0.215249 |
| **NusG** | Transcription termination/antitermination protein NusG | 1.188211 | 0.081404 |
| **AroK** | Shikimate kinase 1 | 1.188289 | 0.000877 |
| **B4U21_25325** | UPF0114 protein B4U21_25325 | 1.189064 | 0.664558 |
| **PurL** | Phosphoribosylformylglycinamidine synthase | 1.189083 | 0.002039 |
| **RibE** | 6,7-dimethyl-8-ribityllumazine synthase | 1.189534 | 0.010633 |
| **CitA** | Histidine kinase | 1.189615 | 0.30865 |
| **HisA** | 1-(5-phosphoribosyl)-5-[(5-phosphoribosylamino)methylideneamino] imidazole-4-carboxamide isomerase | 1.189782 | 0.018447 |
| **RplL** | 50S ribosomal protein L7/L12 | 1.191476 | 0.025149 |
| **DusA** | tRNA-dihydrouridine(20/20a) synthase | 1.191985 | 0.051155 |
| **FusA** | Elongation factor G | 1.19293 | 0.001509 |
| **Lon_2** | Endopeptidase La | 1.19438 | 0.037376 |
| **YibN** | Molybdopterin biosynthesis protein MoeB | 1.194422 | 0.000109 |
| **MlaA** | Lipoprotein | 1.194495 | 0.206304 |
| **MurA** | UDP-N-acetylglucosamine 1-carboxyvinyltransferase | 1.194816 | 0.006437 |
| **GJJ01_15355** | GNAT family N-acetyltransferase | 1.19545 | 0.657853 |
| **BANRA_02224** | tRNA-(Ms[2]io[6]A)-hydroxylase | 1.195745 | 0.233154 |
| **RmuC** | DNA recombination protein RmuC | 1.197085 | 0.027789 |
| **LigT** | RNA 2',3'-cyclic phosphodiesterase | 1.197318 | 0.730728 |
| **CbiF** | Cobalt-precorrin-4 C(11)-methyltransferase | 1.199362 | 0.114049 |
| **YbjD** | ATP-dependent endonuclease | 1.200175 | 0.031649 |
| **Tuf** | Elongation factor Tu (Fragment) | 1.200275 | 0.004909 |
| **PepA** | Probable cytosol aminopeptidase | 1.200333 | 0.001304 |
| **TrmD** | tRNA (guanine-N(1)-)-methyltransferase | 1.20146 | 0.151202 |
| **YhbU_3** | Peptidase | 1.201787 | 0.046173 |
| **NagB_2** | Glucosamine-6-phosphate deaminase | 1.202789 | 0.009452 |
| **CarB** | Carbamoyl-phosphate synthase large chain | 1.206254 | 0.000082 |
| **IlvC** | Ketol-acid reductoisomerase (NADP(+)) | 1.207428 | 0.320052 |
| **NarG** | Nitrate reductase (quinone) | 1.208227 | 0.004959 |
| **Dxs_1** | 1-deoxy-D-xylulose-5-phosphate synthase | 1.208979 | 0.007162 |
| **GatB** | Galactitol-specific phosphotransferase enzyme IIB component | 1.209703 | 0.21656 |
| **RelA** | GTP diphosphokinase | 1.20979 | 0.002588 |
| **TatD** | 3'-5' ssDNA/RNA exonuclease TatD | 1.213218 | 0.014924 |
| **SecD** | Protein translocase subunit SecD | 1.213629 | 0.003022 |
| **AtpA** | ATP synthase subunit alpha | 1.214085 | 0.000135 |
| **LsrK_1** | Pentose kinase | 1.214515 | 0.76289 |
| **BANRA_02252** | Amidohydrolase/deacetylase family metallohydrolase | 1.21489 | 0.265984 |
| **BANRA_00692** | ArsR family transcriptional regulator | 1.215541 | 0.077291 |
| **PspF** | Phage shock protein operon transcriptional activator | 1.216952 | 0.047168 |
| **RsmI** | Ribosomal RNA small subunit methyltransferase I | 1.218387 | 0.069904 |
| **YceD** | 23S rRNA accumulation protein YceD | 1.219086 | 0.005219 |
| **Cnu** | Cnu protein | 1.219689 | 0.007293 |
| **QueA** | S-adenosylmethionine:tRNA ribosyltransferase-isomerase | 1.220429 | 0.007646 |
| **NsrR** | HTH-type transcriptional repressor NsrR | 1.220831 | 0.209114 |
| **PotC** | Spermidine/putrescine ABC transporter membrane protein | 1.221296 | 0.240872 |
| **YojI** | ABC transporter ATP-binding protein | 1.224696 | 0.007243 |
| **PulS** | Lipoprotein, PulS/OutS family | 1.224732 | 0.191795 |
| **AcpS** | Holo-[acyl-carrier-protein] synthase | 1.225452 | 0.212583 |
| **Pth** | Peptidyl-tRNA hydrolase | 1.227105 | 0.258538 |
| **QueE** | 7-carboxy-7-deazaguanine synthase | 1.229032 | 0.032651 |
| **BolA** | BolA family transcriptional regulator | 1.229629 | 0.010307 |
| **PotA_2** | Spermidine/putrescine import ATP-binding protein PotA | 1.23018 | 0.237261 |
| **MurI** | Glutamate racemase | 1.23122 | 0.109105 |
| **YqiC** | Ubiquinone biosynthesis accessory factor UbiK | 1.231911 | 0.004347 |
| **TrmH** | tRNA (guanosine(18)-2'-O)-methyltransferase | 1.232857 | 0.224227 |
| **FtnA_2** | Ferritin | 1.233723 | 0.059027 |
| **PyrH** | Uridylate kinase | 1.233845 | 0.007387 |
| **EpmC** | Elongation factor P hydroxylase | 1.235964 | 0.606775 |
| **FtsQ** | Cell division protein FtsQ | 1.236342 | 0.308311 |
| **Cra_1** | Catabolite repressor/activator | 1.237463 | 0.207749 |
| **PcnB** | Poly(A) polymerase I | 1.238291 | 0.037925 |
| **PrfC** | Peptide chain release factor 3 | 1.241849 | 0.001449 |
| **LpxD** | UDP-3-O-(3-hydroxymyristoyl)glucosamine N-acyltransferase | 1.241902 | 0.003972 |
| **HmsP** | Biofilm formation regulator HmsP | 1.242169 | 0.029537 |
| **DD581_07015** | Acetyltransferase | 1.244018 | 0.00292 |
| **GlgA** | Glycogen synthase | 1.244503 | 0.065069 |
| **NusB** | Transcription antitermination protein NusB | 1.246152 | 0.00774 |
| **FabB** | 3-oxoacyl-ACP synthase | 1.249433 | 0.020448 |
| **GsiA_7** | ABC transporter ATP-binding protein | 1.249707 | 0.017029 |
| **RplS** | 50S ribosomal protein L19 | 1.249735 | 0.011282 |
| **YheS_2** | ABC transporter ATP-binding protein | 1.25302 | 0.013536 |
| **YjaG** | DUF416 domain-containing protein | 1.253348 | 0.015695 |
| **RffG** | dTDP-glucose 4,6-dehydratase | 1.253942 | 0.00757 |
| **SseB** | Enhanced serine sensitivity protein SseB | 1.254067 | 0.108178 |
| **Smp2** | Membrane protein | 1.254667 | 0.283781 |
| **CmoB** | tRNA U34 carboxymethyltransferase | 1.256083 | 0.438871 |
| **TusE** | Sulfurtransferase | 1.256874 | 0.103351 |
| **B4U25_14985** | DUF4186 domain-containing protein | 1.257286 | 0.028016 |
| **YaaA** | UPF0246 protein B4U21_00050 | 1.259645 | 0.042964 |
| **RpsB** | 30S ribosomal protein S2 | 1.260786 | 0.032025 |
| **RimI** | [Ribosomal protein S18]-alanine N-acetyltransferase | 1.26102 | 0.044734 |
| **YjgA** | UPF0307 protein B4U21_32930 | 1.262582 | 0.065504 |
| **Ffh** | Signal recognition particle protein | 1.263105 | 0.001068 |
| **SdaB** | L-serine dehydratase | 1.263123 | 0.002245 |
| **MrcA** | DD-transpeptidase | 1.266848 | 0.014722 |
| **FrdB** | Fumarate reductase iron-sulfur subunit | 1.268394 | 0.214415 |
| **FabI** | Enoyl-[acyl-carrier-protein] reductase [NADH] | 1.269689 | 0.003173 |
| **CobT** | Nicotinate-nucleotide--dimethylbenzimidazole phosphoribosyltransferase | 1.269696 | 0.690337 |
| **HisG** | ATP phosphoribosyltransferase | 1.271821 | 0.088378 |
| **MurR** | HTH-type transcriptional regulator MurR | 1.273896 | 0.071289 |
| **C2U49_13320** | Carboxymuconolactone decarboxylase family protein | 1.273973 | 0.068092 |
| **YieH** | 6-phosphogluconate phosphatase | 1.275444 | 0.208581 |
| **WaaA** | 3-deoxy-D-manno-octulosonic acid transferase | 1.277088 | 0.143985 |
| **ObgE** | GTPase Obg | 1.278428 | 0.126647 |
| **ThrS** | Threonine--tRNA ligase | 1.278472 | 0.000916 |
| **MntR** | Manganese transport regulator | 1.279653 | 0.022208 |
| **PepT** | Peptidase T | 1.282532 | 0.019235 |
| **YjaB_2** | Acetyltransferase | 1.28452 | 0.001094 |
| **YqeG** | Inner membrane transport protein YqeG | 1.286612 | 0.007491 |
| **MenB** | 1,4-dihydroxy-2-naphthoyl-CoA synthase | 1.287293 | 0.041417 |
| **AccA** | Acetyl-coenzyme A carboxylase carboxyl transferase subunit alpha | 1.291831 | 0.001312 |
| **RpmD** | 50S ribosomal protein L30 | 1.293471 | 0.049954 |
| **NarL** | DNA-binding response regulator | 1.293493 | 0.005544 |
| **GJJ01_27690** | Cupin domain-containing protein | 1.294004 | 0.005341 |
| **MalE** | Maltodextrin-binding protein | 1.294961 | 0.004695 |
| **NusA** | Transcription termination/antitermination protein NusA | 1.295202 | 0.001659 |
| **AtpH** | ATP synthase subunit delta | 1.296673 | 0.004893 |
| **GlyS** | Glycine--tRNA ligase beta subunit | 1.29694 | 0.000006 |
| **ParC** | DNA topoisomerase 4 subunit A | 1.29723 | 0.004265 |
| **DedD** | Cell division protein DedD | 1.297624 | 0.005312 |
| **RpoE** | ECF RNA polymerase sigma-E factor | 1.298086 | 0.004248 |
| **YgeR** | LysM peptidoglycan-binding domain-containing protein | 1.298181 | 0.06794 |
| **PdxJ** | Pyridoxine 5'-phosphate synthase | 1.298524 | 0.576105 |
| **PyrB** | Aspartate carbamoyltransferase | 1.298741 | 0.008489 |
| **DnaC** | DNA replication protein DnaC | 1.299365 | 0.001821 |
| **IaaA** | Beta-aspartyl-peptidase | 1.299986 | 0.000854 |
| **Rnd** | Ribonuclease D | 1.302966 | 0.012996 |
| **TamB** | Autotransporter assembly complex protein TamB | 1.303598 | 0.000448 |
| **IlvG** | Acetolactate synthase | 1.305824 | 0.410042 |
| **RpoB** | DNA-directed RNA polymerase subunit beta | 1.306092 | 0.000175 |
| **RnfE** | Ion-translocating oxidoreductase complex subunit E | 1.30683 | 0.337475 |
| **ExbD** | Biopolymer transport protein ExbD | 1.308009 | 0.00098 |
| **EntB** | Isochorismatase | 1.309511 | 0.00322 |
| **SmrA_1** | UPF0115 protein B6R99_06315 | 1.3101 | 0.294825 |
| **NarY** | NarY protein | 1.311187 | 0.611162 |
| **NemR** | HTH-type transcriptional repressor NemR | 1.313273 | 0.001899 |
| **FbaB** | Class I fructose-bisphosphate aldolase | 1.313461 | 0.002695 |
| **ThiI** | tRNA sulfurtransferase | 1.317497 | 0.001971 |
| **YeeX** | UPF0265 protein B4U21_18700 | 1.318191 | 0.010061 |
| **PitA** | Phosphate transporter | 1.318497 | 0.000207 |
| **RpoZ** | DNA-directed RNA polymerase subunit omega | 1.318551 | 0.01878 |
| **PgpB** | PgpB protein | 1.319239 | 0.100214 |
| **Imp** | LPS-assembly protein LptD | 1.319941 | 0.194527 |
| **YrfG** | (S)-2-haloacid dehalogenase | 1.320839 | 0.104594 |
| **RfaC** | ADP-heptose--LPS heptosyltransferase | 1.321432 | 0.000889 |
| **YggT** | Integral membrane protein YggT | 1.321571 | 0.168448 |
| **LpdA** | Dihydrolipoyl dehydrogenase | 1.322143 | 0.000425 |
| **MatP** | Macrodomain Ter protein | 1.322908 | 0.046725 |
| **SrlD** | Sorbitol-6-phosphate 2-dehydrogenase | 1.323504 | 0.003545 |
| **AsnS** | Asparagine--tRNA ligase | 1.323555 | 0.000158 |
| **ProQ** | RNA chaperone ProQ | 1.323628 | 0.000505 |
| **SufD** | Fe-S cluster assembly protein SufD | 1.323649 | 0.004703 |
| **NlpI** | Lipoprotein NlpI | 1.324385 | 0.039793 |
| **PfkA** | ATP-dependent 6-phosphofructokinase | 1.325738 | 0.000017 |
| **PheT** | Phenylalanine--tRNA ligase beta subunit | 1.326629 | 0.000152 |
| **BtuB** | Vitamin B12 transporter BtuB | 1.328196 | 0.052848 |
| **NuoL** | NADH-quinone oxidoreductase subunit L | 1.330935 | 0.011787 |
| **SdhD** | Succinate dehydrogenase hydrophobic membrane anchor subunit | 1.331288 | 0.043225 |
| **NhaB** | Na(+)/H(+) antiporter NhaB | 1.331555 | 0.15113 |
| **SrlB** | PTS glucitol/sorbitol transporter subunit IIA | 1.334021 | 0.005333 |
| **YjiR** | Aminotransferase class I/II-fold pyridoxal phosphate-dependent enzyme | 1.334046 | 0.378205 |
| **InfC** | Translation initiation factor IF-3 | 1.334548 | 0.007611 |
| **RsuA** | Pseudouridine synthase | 1.334819 | 0.001974 |
| **YdiK_2** | AI-2E family transporter | 1.335151 | 0.275936 |
| **Wzi** | Capsule assembly Wzi family protein | 1.336714 | 0.267081 |
| **RsxB** | Ion-translocating oxidoreductase complex subunit B | 1.336741 | 0.507147 |
| **MurF** | UDP-N-acetylmuramoyl-tripeptide--D-alanyl-D-alanine ligase | 1.337275 | 0.003696 |
| **GltR_2** | LysR family transcriptional regulator | 1.338732 | 0.437268 |
| **TsaE** | t(6)A37 threonylcarbamoyladenosine biosynthesis protein TsaE | 1.338812 | 0.000141 |
| **UbiH** | 2-octaprenyl-6-methoxyphenol hydroxylase | 1.340079 | 0.040897 |
| **OmpR_1** | DNA-binding response regulator | 1.34034 | 0.609797 |
| **TrmB** | tRNA (guanine-N(7)-)-methyltransferase | 1.34036 | 0.050813 |
| **GyrB** | DNA gyrase subunit B | 1.343482 | 0.000089 |
| **GuaA** | GMP synthase [glutamine-hydrolyzing] | 1.344057 | 0.000138 |
| **DacA** | Serine-type D-Ala-D-Ala carboxypeptidase | 1.344863 | 0.000565 |
| **IolC_3** | 5-dehydro-2-deoxygluconokinase | 1.345389 | 0.071485 |
| **HrpA** | ATP-dependent RNA helicase HrpA | 1.346606 | 0.009088 |
| **YcbX_2** | 2Fe-2S ferredoxin YfaE | 1.351057 | 0.01944 |
| **MscS** | Mechanosensitive channel MscS | 1.351109 | 0.013973 |
| **ArgR** | Arginine repressor | 1.351614 | 0.03005 |
| **SecA** | Protein translocase subunit SecA | 1.351642 | 0.000117 |
| **YdjN** | Cation:dicarboxylase symporter family transporter | 1.351707 | 0.091255 |
| **PrmC** | Release factor glutamine methyltransferase | 1.355895 | 0.112387 |
| **CytR_2** | LacI family DNA-binding transcriptional regulator | 1.356505 | 0.511782 |
| **B4U21_06905** | Bacterial sensory transduction regulator | 1.356968 | 0.016321 |
| **HxlR** | HTH-type transcriptional activator HxlR | 1.357434 | 0.020918 |
| **Upp** | Uracil phosphoribosyltransferase | 1.357719 | 0.001085 |
| **NtrB** | Histidine kinase | 1.359173 | 0.018127 |
| **CadC** | CadC family transcriptional regulator | 1.359303 | 0.039882 |
| **CyoA** | Ubiquinol oxidase subunit 2 | 1.360455 | 0.00192 |
| **EntS** | Enterobactin exporter EntS | 1.361101 | 0.564459 |
| **SelB** | SelB translation factor | 1.362775 | 0.095451 |
| **YabJ_1** | 2-iminobutanoate/2-iminopropanoate deaminase | 1.362939 | 0.013611 |
| **PotF_1** | ABC transporter substrate-binding protein | 1.364482 | 0.001276 |
| **YffB** | A glutathione-dependent thiol reductase | 1.366367 | 0.032526 |
| **MscS** | Mechanosensitive channel MscK | 1.366798 | 0.009703 |
| **GJJ01_16205** | Alpha-2-macroglobulin | 1.372783 | 0.011805 |
| **GJJ01_21115** | 5'-3' exoribonuclease | 1.373192 | 0.030285 |
| **ProS** | Proline--tRNA ligase | 1.374282 | 0.002709 |
| **CysK_2** | Cysteine synthase | 1.374643 | 0.00958 |
| **HemG** | HemG protein | 1.376598 | 0.005188 |
| **FtsK** | DNA translocase FtsK | 1.381396 | 0.018795 |
| **ZraR_2** | Response regulator of zinc sigma-54-dependent two-component system | 1.38249 | 0.559007 |
| **RpsP** | 30S ribosomal protein S16 | 1.385687 | 0.03662 |
| **Spr** | Bifunctional murein DD-endopeptidase/murein LD-carboxypeptidase | 1.387354 | 0.000649 |
| **YhaX** | Cof-type HAD-IIB family hydrolase | 1.38746 | 0.326284 |
| **GlnA** | Glutamine synthetase | 1.391089 | 0.003379 |
| **CmpR_1** | HTH-type transcriptional activator CmpR | 1.392076 | 0.406915 |
| **RplA** | 50S ribosomal protein L1 | 1.392317 | 0.000298 |
| **RsmB** | Ribosomal RNA small subunit methyltransferase B | 1.392724 | 0.007926 |
| **HisH** | Imidazole glycerol phosphate synthase subunit HisH | 1.393164 | 0.008527 |
| **RluC** | Pseudouridine synthase | 1.393716 | 0.001096 |
| **RplF** | 50S ribosomal protein L6 | 1.395529 | 0.001114 |
| **KamA** | EF-P post-translational modification enzyme B | 1.397112 | 0.013432 |
| **PurP** | NCS2 family permease | 1.398496 | 0.278903 |
| **PurU** | Formyltetrahydrofolate deformylase | 1.398591 | 0.019984 |
| **RplB** | 50S ribosomal protein L2 | 1.399344 | 0.003854 |
| **YaiI** | UPF0178 protein B6R99_07575 | 1.401203 | 0.004247 |
| **Pnp** | Polyribonucleotide nucleotidyltransferase | 1.402937 | 0.000142 |
| **CstA** | Carbon starvation protein A | 1.403166 | 0.276155 |
| **OxyR** | DNA-binding transcriptional regulator OxyR | 1.403453 | 0.00275 |
| **B4U21_27805** | Death on curing protein, Doc toxin | 1.406071 | 0.184131 |
| **BL124_00003435** | DUF4056 domain-containing protein | 1.406176 | 0.011912 |
| **MalK_3** | Maltose/maltodextrin import ATP-binding protein MalK | 1.407634 | 0.00285 |
| **CmpR_4** | HTH-type transcriptional activator CmpR | 1.40813 | 0.541004 |
| **WzzE** | ECA polysaccharide chain length modulation protein | 1.411079 | 0.007502 |
| **Syd** | Protein Syd | 1.411882 | 0.088758 |
| **NupC** | Nucleoside permease | 1.412843 | 0.007257 |
| **YcaO** | 30S ribosomal protein S12 methylthiotransferase accessory protein YcaO | 1.412914 | 0.018603 |
| **RpsJ** | 30S ribosomal protein S10 | 1.414062 | 0.009449 |
| **NadB** | L-aspartate oxidase | 1.415903 | 0.045411 |
| **RplM** | 50S ribosomal protein L13 | 1.415911 | 0.000318 |
| **WaaF** | ADP-heptose--LPS heptosyltransferase RfaF | 1.416297 | 0.314012 |
| **ProV** | Glycine betaine/L-proline ABC transporter ATP-binding protein | 1.416302 | 0.011618 |
| **FtsA** | Cell division protein FtsA | 1.41861 | 0.000403 |
| **YbjI** | 5-amino-6-(5-phospho-D-ribitylamino)uracil phosphatase YbjI | 1.418691 | 0.026248 |
| **MalM** | MalM protein | 1.42017 | 0.002051 |
| **Pta** | Phosphate acetyltransferase | 1.421944 | 0.000052 |
| **RpmJ** | 50S ribosomal protein L36 | 1.423953 | 0.000253 |
| **BltD** | GNAT family N-acetyltransferase | 1.425123 | 0.060584 |
| **CadA** | Inducible lysine decarboxylase | 1.427832 | 0.003016 |
| **AnsA** | Asparaginase | 1.429003 | 0.005443 |
| **IbpA** | Small heat shock protein IbpA | 1.43157 | 0.141849 |
| **AcrD** | Efflux pump membrane transporter | 1.432324 | 0.031482 |
| **CoaBC** | Coenzyme A biosynthesis bifunctional protein CoaBC | 1.433119 | 0.011336 |
| **GlsB** | Glutaminase | 1.434235 | 0.00215 |
| **RpmB** | 50S ribosomal protein L28 | 1.435173 | 0.000668 |
| **Lrp_1** | DNA-binding transcriptional activator DecR | 1.435612 | 0.635013 |
| **RplD** | 50S ribosomal protein L4 | 1.437004 | 0.000222 |
| **KdsC** | 3-deoxy-D-manno-octulosonate 8-phosphate phosphatase KdsC | 1.43864 | 0.12365 |
| **PrmA** | Ribosomal protein L11 methyltransferase | 1.439768 | 0.000279 |
| **AmyA** | Alpha-amylase | 1.441676 | 0.058429 |
| **RecB** | RecBCD enzyme subunit RecB | 1.442279 | 0.007369 |
| **MoaC** | Cyclic pyranopterin monophosphate synthase | 1.445486 | 0.078711 |
| **YbiU** | DUF1479 family protein | 1.445894 | 0.024263 |
| **RecX** | Regulatory protein RecX | 1.446395 | 0.056074 |
| **SecA** | UPF0149 family protein | 1.447456 | 0.002158 |
| **MetN_3** | Methionine import ATP-binding protein MetN | 1.448147 | 0.093356 |
| **GJJ01_05905** | Iron-regulated membrane protein | 1.448577 | 0.008065 |
| **YjjG** | 5'-nucleotidase | 1.448899 | 0.062984 |
| **YeaC** | DUF1315 domain-containing protein | 1.45091 | 0.007522 |
| **RpsN** | 30S ribosomal protein S14 | 1.452361 | 0.012083 |
| **RecC** | RecBCD enzyme subunit RecC | 1.45628 | 0.018465 |
| **IutA** | Putative TonB-dependent receptor | 1.45638 | 0.143578 |
| **FdoI** | FdoI protein | 1.456463 | 0.240275 |
| **B4U21_12950** | ParD-like family protein | 1.457548 | 0.114459 |
| **LptF** | Lipopolysaccharide export system permease protein LptF | 1.457591 | 0.114564 |
| **FepA_3** | TonB-dependent receptor | 1.459386 | 0.079889 |
| **RimO** | Ribosomal protein S12 methylthiotransferase RimO | 1.460788 | 0.000007 |
| **WaaE** | Glucosyltransferase WaaE | 1.461384 | 0.004706 |
| **PykF** | Pyruvate kinase | 1.462592 | 0.000022 |
| **B4U21_20510** | YfcL family protein | 1.46316 | 0.019899 |
| **DbpA** | ATP-dependent RNA helicase DbpA | 1.463878 | 0.042951 |
| **RplI** | 50S ribosomal protein L9 | 1.463905 | 0.001638 |
| **RpsT** | 30S ribosomal protein S20 | 1.464476 | 0.000555 |
| **MalF** | Maltose/maltodextrin transport system permease protein | 1.465901 | 0.00272 |
| **RstA** | DNA-binding response regulator | 1.468658 | 0.024551 |
| **B4U25_19730** | RidA family protein | 1.469687 | 0.452946 |
| **LacC** | Phosphofructokinase | 1.469877 | 0.015807 |
| **RluA** | Pseudouridine synthase | 1.470306 | 0.001135 |
| **YebE** | DUF533 domain-containing protein | 1.471611 | 0.192923 |
| **ZnuA** | High-affinity zinc uptake system protein ZnuA | 1.472306 | 0.002446 |
| **Pat** | Protein acetyltransferase | 1.474413 | 0.341721 |
| **QueG** | Epoxyqueuosine reductase | 1.476724 | 0.190245 |
| **AgaR** | DeoR family transcriptional regulator | 1.477665 | 0.005018 |
| **XerC** | Tyrosine recombinase XerC | 1.480109 | 0.22592 |
| **FecD** | ABC transporter permease | 1.480111 | 0.232536 |
| **ClcA** | H(+)/Cl(-) exchange transporter ClcA | 1.480442 | 0.61721 |
| **YicC** | Protein YicC | 1.483441 | 0.002089 |
| **RatB** | UPF0125 protein B6R99_20980 | 1.485244 | 0.010855 |
| **RcsC** | Sensor histidine kinase RcsC | 1.485304 | 0.003751 |
| **InfB** | Translation initiation factor IF-2 | 1.489782 | 0.000061 |
| **TsaD** | tRNA N6-adenosine threonylcarbamoyltransferase | 1.490594 | 0.004293 |
| **Spr_3** | Endopeptidase | 1.490838 | 0.000196 |
| **RpsL** | 30S ribosomal protein S12 | 1.490864 | 0.000257 |
| **AckA_2** | Acetate kinase | 1.490913 | 0.000992 |
| **RfaQ** | Lipopolysaccharide heptosyltransferase III | 1.493555 | 0.259745 |
| **SecY** | Protein translocase subunit SecY | 1.494446 | 0.058272 |
| **BN49_1157** | Ribosome association toxin RatA | 1.495869 | 0.028786 |
| **Edd** | Phosphogluconate dehydratase | 1.497894 | 0.000031 |
| **MtnC** | Enolase-phosphatase E1 | 1.498966 | 0.022104 |
| **RlmH** | Ribosomal RNA large subunit methyltransferase H | 1.504129 | 0.193694 |
| **PyrG** | CTP synthase | 1.506807 | 0.00005 |
| **PrsA** | Ribose-phosphate pyrophosphokinase | 1.508749 | 0.000149 |
| **GuaB** | Inosine-5'-monophosphate dehydrogenase | 1.509187 | 0.000868 |
| **NlpA** | Lipoprotein | 1.509647 | 0.01764 |
| **EngD** | Ribosome-binding ATPase YchF | 1.510441 | 0.000242 |
| **ErpA** | Iron-sulfur cluster insertion protein ErpA | 1.511557 | 0.00173 |
| **RpmH** | 50S ribosomal protein L34 | 1.512243 | 0.055832 |
| **YccS** | Efflux (PET) family inner membrane protein YccS | 1.513499 | 0.012529 |
| **CysJ** | Sulfite reductase [NADPH] flavoprotein alpha-component | 1.514644 | 0.000125 |
| **Cfa** | Cfa protein | 1.515064 | 0.000217 |
| **RpsC** | 30S ribosomal protein S3 | 1.515727 | 0.000059 |
| **Udk** | Uridine kinase | 1.517024 | 0.011017 |
| **MnmE** | tRNA modification GTPase MnmE | 1.52177 | 0.000103 |
| **HyaD** | Glycosyltransferase | 1.524658 | 0.069464 |
| **Rho** | Transcription termination factor Rho | 1.526347 | 0.00018 |
| **TruB** | tRNA pseudouridine synthase B | 1.527455 | 0.001426 |
| **C2U49_08720** | LexA family transcriptional regulator | 1.530287 | 0.275521 |
| **RplT** | 50S ribosomal protein L20 | 1.532011 | 0.00455 |
| **MlaE** | Intermembrane phospholipid transport system permease protein MlaE | 1.53212 | 0.021663 |
| **NhaK_2** | Na+/H+ antiporter | 1.540953 | 0.083356 |
| **YoaE_2** | CNNM family cation transport protein YoaE | 1.541997 | 0.00184 |
| **Dam_2** | Site-specific DNA-methyltransferase (adenine-specific) | 1.542424 | 0.002301 |
| **LldD_1** | Alpha-hydroxy-acid oxidizing protein | 1.54245 | 0.001011 |
| **YbbL** | Iron ABC transporter ATP-binding protein FetA | 1.543723 | 0.000029 |
| **MurJ** | Probable lipid II flippase MurJ | 1.545587 | 0.021341 |
| **GntR_2** | Gluconate operon transcriptional repressor GntR | 1.546397 | 0.088851 |
| **GsiA_11** | ABC transporter ATP-binding protein | 1.54646 | 0.002738 |
| **GntU** | Gluconate transporter | 1.547927 | 0.050046 |
| **HisC** | Histidinol-phosphate aminotransferase | 1.548501 | 0.001741 |
| **XseB** | Exodeoxyribonuclease 7 small subunit | 1.551076 | 0.003941 |
| **SrlR_3** | DeoR family transcriptional regulator | 1.555636 | 0.084182 |
| **RplR** | 50S ribosomal protein L18 | 1.556776 | 0.000019 |
| **GlpA** | Glycerol-3-phosphate dehydrogenase | 1.557426 | 0.009403 |
| **C2U49_00080** | 5-oxoprolinase/urea amidolyase family protein | 1.557428 | 0.00201 |
| **RlmG** | Ribosomal RNA large subunit methyltransferase G | 1.559636 | 0.002078 |
| **CmoA** | Carboxy-S-adenosyl-L-methionine synthase | 1.560088 | 0.001516 |
| **NudJ** | Phosphatase NudJ | 1.56079 | 0.091488 |
| **RplN** | 50S ribosomal protein L14 | 1.562042 | 0.000357 |
| **BglF_2** | PTS beta-glucoside transporter subunit IIABC | 1.563229 | 0.006484 |
| **NuoE** | NADH dehydrogenase I subunit E | 1.563779 | 0.0004 |
| **RpsE** | 30S ribosomal protein S5 | 1.564817 | 0.000448 |
| **Wzt** | ABC transporter ATP-binding protein | 1.56483 | 0.041858 |
| **YhjQ** | Cellulose synthase operon protein YhjQ | 1.565433 | 0.13551 |
| **RpoH** | RNA polymerase sigma factor RpoH | 1.565891 | 0.003496 |
| **RpsR** | 30S ribosomal protein S18 | 1.566233 | 0.001888 |
| **MraY** | Phospho-N-acetylmuramoyl-pentapeptide-transferase | 1.566669 | 0.001276 |
| **LipA** | Lipoyl synthase | 1.567911 | 0.000823 |
| **MalZ** | Maltodextrin glucosidase | 1.568947 | 0.01245 |
| **Irp1** | High-molecular-weight nonribosomal peptide/polyketide synthetase 1 | 1.570937 | 0.568741 |
| **AtpD** | ATP synthase subunit beta | 1.572629 | 0.000008 |
| **GlpK_1** | Carbohydrate kinase | 1.575014 | 0.57194 |
| **EntC** | Isochorismate synthase | 1.58542 | 0.022175 |
| **PlsB** | Glycerol-3-phosphate acyltransferase | 1.586466 | 0.002752 |
| **NrdD** | Anaerobic ribonucleoside-triphosphate reductase | 1.588822 | 0.010836 |
| **NqrC** | Na(+)-translocating NADH-quinone reductase subunit C | 1.596277 | 0.019004 |
| **GJJ01_27620** | DNA polymerase III subunit theta | 1.59632 | 0.127706 |
| **SelA** | L-seryl-tRNA(Sec) selenium transferase | 1.596978 | 0.00106 |
| **BANRA_02570** | Protein PaaI | 1.600138 | 0.001866 |
| **MtnB** | Methylthioribulose-1-phosphate dehydratase | 1.600353 | 0.437192 |
| **PflB** | Formate C-acetyltransferase | 1.601837 | 0.00053 |
| **YeiG** | S-formylglutathione hydrolase | 1.602382 | 0.005007 |
| **DegA_2** | LacI family DNA-binding transcriptional regulator | 1.604563 | 0.07145 |
| **DhaR** | Acetoin catabolism regulatory protein | 1.606493 | 0.263273 |
| **LivF** | High-affinity branched-chain amino acid transport ATP-binding protein | 1.609709 | 0.023008 |
| **Cca** | Multifunctional CCA protein | 1.610727 | 0.00267 |
| **YheT** | Hydrolase | 1.611843 | 0.112523 |
| **LdcA** | Muramoyltetrapeptide carboxypeptidase | 1.616133 | 0.012806 |
| **RsfS** | Ribosomal silencing factor RsfS | 1.61862 | 0.003997 |
| **LpxB** | Lipid-A-disaccharide synthase | 1.623743 | 0.088218 |
| **MiaB** | tRNA-2-methylthio-N(6)-dimethylallyladenosine synthase | 1.625201 | 0.000292 |
| **GJJ01_13315** | GNAT family N-acetyltransferase | 1.625416 | 0.254256 |
| **DmlR_9** | LysR family transcriptional regulator | 1.629018 | 0.225455 |
| **RplC** | 50S ribosomal protein L3 | 1.62982 | 0.000179 |
| **PstC** | Phosphate transport system permease protein | 1.631441 | 0.051123 |
| **FhuC** | Car(A)_1_M80346 | 1.631674 | 0.001542 |
| **TrpR** | Trp operon repressor | 1.633097 | 0.008469 |
| **TtdT** | Anion permease | 1.636104 | 0.013641 |
| **BioD_2** | ATP-dependent dethiobiotin synthetase BioD | 1.638023 | 0.001462 |
| **RplO** | 50S ribosomal protein L15 | 1.639217 | 0.000782 |
| **AroL** | Shikimate kinase 1 | 1.640329 | 0.003153 |
| **GJJ01_06720** | Metal-dependent hydrolase | 1.642617 | 0.004791 |
| **BANRA_03124** | ISNCY family transposase | 1.64562 | 0.030647 |
| **HcaR_1** | Hca operon transcriptional activator | 1.649102 | 0.097808 |
| **LpxT** | Lipid A 1-diphosphate synthase | 1.65152 | 0.000195 |
| **BANRA_00262** | Uncharacterized protein | 1.653836 | 0.024064 |
| **TruC** | TruC protein | 1.654097 | 0.001221 |
| **NarH** | Nitrate reductase subunit beta | 1.654123 | 0.005889 |
| **YgjP** | DUF45 domain-containing protein | 1.654957 | 0.000436 |
| **RpsD** | 30S ribosomal protein S4 | 1.655362 | 0.00004 |
| **UraH** | 5-hydroxyisourate hydrolase | 1.656371 | 0.048408 |
| **MoaB** | Molybdenum cofactor biosynthesis protein B | 1.658417 | 0.092679 |
| **SufS** | Cysteine desulfurase | 1.658839 | 0.023294 |
| **MtnK** | Methylthioribose kinase | 1.659449 | 0.000406 |
| **PabA** | Aminodeoxychorismate synthase component 2 | 1.677112 | 0.251819 |
| **BANRA_01373** | DNA-binding protein | 1.681879 | 0.072401 |
| **BaeS** | Histidine kinase | 1.681985 | 0.048465 |
| **Gcd** | Gcd protein | 1.682341 | 0.004077 |
| **EnvC** | Murein hydrolase activator EnvC | 1.683521 | 0.015584 |
| **TrkA** | Trk system potassium uptake protein TrkA | 1.685457 | 0.067697 |
| **YciN** | DUF2498 domain-containing protein | 1.691468 | 0.026031 |
| **CsrD** | Lipoprotein | 1.694111 | 0.004702 |
| **FdhD** | Sulfur carrier protein FdhD | 1.698198 | 0.082197 |
| **YbbH_4** | HTH-type transcriptional regulator HexR | 1.702189 | 0.003158 |
| **FeoA** | Fe(2+) transport protein A | 1.7041 | 0.005898 |
| **EntE** | (2,3-dihydroxybenzoyl)adenylate synthase | 1.705187 | 0.000481 |
| **OmpW** | Outer membrane protein OmpW | 1.706998 | 0.031832 |
| **YjcD** | Guanine-hypoxanthine permease | 1.707234 | 0.040079 |
| **DacB** | D-alanyl-D-alanine carboxypeptidase | 1.710029 | 0.015333 |
| **PolA** | DNA polymerase I | 1.711175 | 0.000172 |
| **TsgA** | Protein TsgA homolog | 1.714399 | 0.001734 |
| **YfiR** | TetR family transcriptional regulator | 1.715405 | 0.001217 |
| **AceE** | Pyruvate dehydrogenase E1 component | 1.717303 | 0.00001 |
| **Mqo** | Probable malate:quinone oxidoreductase | 1.717959 | 0.003682 |
| **RhlE** | ATP-dependent RNA helicase RhlE | 1.719062 | 0.000002 |
| **HolE_1** | DNA polymerase III subunit theta | 1.720015 | 0.012269 |
| **B4U21_25500** | UPF0441 protein B4U21_25500 | 1.725125 | 0.002191 |
| **BANRA_01179** | Membrane protein | 1.726206 | 0.00158 |
| **NorR** | Anaerobic nitric oxide reductase transcription regulator NorR | 1.727087 | 0.002415 |
| **FtsN** | Cell division protein FtsN | 1.727836 | 0.000315 |
| **Rph** | Ribonuclease PH | 1.72856 | 0.016426 |
| **RplX** | 50S ribosomal protein L24 | 1.73161 | 0.000517 |
| **RplJ** | 50S ribosomal protein L10 | 1.733155 | 0.000123 |
| **EXT45_28075** | Recombinase | 1.734304 | 0.264061 |
| **MnmG** | tRNA uridine 5-carboxymethylaminomethyl modification enzyme MnmG | 1.738878 | 0.039148 |
| **YbiX** | PKHD-type hydroxylase B6R99_16635 | 1.741282 | 0.297593 |
| **AtpG** | ATP synthase gamma chain | 1.741696 | 0.00012 |
| **RecR** | Recombination protein RecR | 1.743897 | 0.001814 |
| **FolE2** | GTP cyclohydrolase FolE2 | 1.746387 | 0.003394 |
| **TyrR** | Transcriptional regulatory protein TyrR | 1.748097 | 0.00077 |
| **IlvB_2** | Thiamine pyrophosphate enzyme | 1.748475 | 0.046768 |
| **Livk** | High-affinity branched-chain amino acid ABC transporter substrate-binding protein LivK | 1.752419 | 0.137917 |
| **B4U25_35990** | LacI family DNA-binding transcriptional regulator | 1.752665 | 0.024754 |
| **RplQ** | 50S ribosomal protein L17 | 1.757042 | 0.000981 |
| **YbcJ** | Ribosome-associated protein | 1.75724 | 0.006671 |
| **SrmB** | ATP-dependent RNA helicase SrmB | 1.75808 | 0.000748 |
| **MrdA** | Peptidoglycan D,D-transpeptidase MrdA | 1.759157 | 0.003431 |
| **RpsH** | 30S ribosomal protein S8 | 1.762205 | 0.000463 |
| **ExbB** | Biopolymer transport protein ExbB | 1.765326 | 0.00106 |
| **MbtH** | Cytoplasmic protein YbdZ | 1.767316 | 0.112038 |
| **MhpD_2** | 2-keto-4-pentenoate hydratase | 1.768555 | 0.134856 |
| **DmsB** | Anaerobic dimethyl sulfoxide reductase chain B | 1.77742 | 0.114053 |
| **YiiF** | CopG family transcriptional regulator | 1.779578 | 0.019123 |
| **ClpS** | ATP-dependent Clp protease adapter protein ClpS | 1.78108 | 0.000646 |
| **SdaC_1** | HAAAP family serine/threonine permease | 1.78321 | 0.037849 |
| **PagP** | Lipid A palmitoyltransferase PagP | 1.784267 | 0.023708 |
| **HelD** | DNA helicase | 1.788999 | 0.065155 |
| **B4U21_15950** | GMC family oxidoreductase | 1.789104 | 0.016633 |
| **RplE** | 50S ribosomal protein L5 | 1.79099 | 0.001096 |
| **RpsA** | 30S ribosomal protein S1 | 1.794288 | 0.000271 |
| **FccA** | Urocanate reductase | 1.795368 | 0.000021 |
| **MukF** | Chromosome partition protein MukF | 1.79545 | 0.00626 |
| **GpmI** | 2,3-bisphosphoglycerate-independent phosphoglycerate mutase | 1.796387 | 0.000562 |
| **MoeZ** | tRNA uridine(34) hydroxylase | 1.80165 | 0.000164 |
| **YbfE** | LexA regulated protein | 1.80175 | 0.00035 |
| **MreB** | Cell shape-determining protein MreB | 1.804843 | 0.000906 |
| **UhpA_2** | DNA-binding response regulator | 1.807836 | 0.016646 |
| **RsmE** | Ribosomal RNA small subunit methyltransferase E | 1.808605 | 0.000558 |
| **RpsG** | 30S ribosomal protein S7 | 1.809661 | 0.000295 |
| **RplP** | 50S ribosomal protein L16 | 1.81066 | 0.000186 |
| **RsmC** | Ribosomal RNA small subunit methyltransferase C | 1.813449 | 0.00042 |
| **RlmN** | Dual-specificity RNA methyltransferase RlmN | 1.813923 | 0.000007 |
| **BepD** | MdtA/MuxA family multidrug efflux RND transporter periplasmic adaptor subunit | 1.816548 | 0.008727 |
| **TolC** | Outer membrane channel protein | 1.81749 | 0.099614 |
| **LipB** | Octanoyltransferase | 1.819705 | 0.00164 |
| **Ipk** | 4-diphosphocytidyl-2-C-methyl-D-erythritol kinase | 1.820808 | 0.00039 |
| **YibH_2** | Auxiliary transport protein, membrane fusion protein (MFP) family | 1.821525 | 0.000674 |
| **BarA** | Histidine kinase | 1.824501 | 0.00891 |
| **RavA** | ATPase RavA | 1.829864 | 0.000852 |
| **YhhW_1** | Pirin family protein | 1.836376 | 0.046767 |
| **CysD** | Sulfate adenylyltransferase subunit 2 | 1.840102 | 0.329519 |
| **FocA** | Formate channel 1 | 1.840965 | 0.037329 |
| **RplV** | 50S ribosomal protein L22 | 1.841588 | 0.000013 |
| **YqhC** | AraC family transcriptional regulator | 1.843127 | 0.096139 |
| **AroQ** | 3-dehydroquinate dehydratase | 1.849103 | 0.094892 |
| **EpmA** | Elongation factor P--(R)-beta-lysine ligase | 1.85387 | 0.001104 |
| **CoaA** | Pantothenate kinase | 1.854803 | 0.00067 |
| **IscA** | Iron-binding protein IscA | 1.860325 | 0.004048 |
| **MalT** | HTH-type transcriptional regulator MalT | 1.873099 | 0.001727 |
| **SirB1** | Protein SirB1 | 1.880772 | 0.004981 |
| **SuhB_2** | Inositol monophosphatase | 1.881133 | 0.00276 |
| **AaeA** | p-hydroxybenzoic acid efflux pump subunit AaeA | 1.881504 | 0.020919 |
| **GltC_2** | LysR family transcriptional regulator | 1.882934 | 0.192832 |
| **MetQ_3** | Lipoprotein | 1.883622 | 0.005923 |
| **RplK** | 50S ribosomal protein L11 | 1.886262 | 0.000087 |
| **DmlR_2** | HTH-type transcriptional regulator DmlR | 1.887731 | 0.155839 |
| **NirB** | NirB protein | 1.89094 | 0.000137 |
| **BaeR** | DNA-binding response regulator | 1.891236 | 0.055833 |
| **YdjA** | Putative NAD(P)H nitroreductase | 1.893617 | 0.000255 |
| **RapA** | RNA polymerase-associated protein RapA | 1.904796 | 0.000309 |
| **RimM** | Ribosome maturation factor RimM | 1.915902 | 0.000046 |
| **PdhR** | Pyruvate dehydrogenase complex repressor | 1.917536 | 0.000198 |
| **RpmC** | 50S ribosomal protein L29 | 1.92156 | 0.000061 |
| **NtpA** | Dihydroneopterin triphosphate diphosphatase | 1.923676 | 0.004465 |
| **TrpE** | Anthranilate synthase component 1 | 1.927021 | 0.001262 |
| **MscL** | Large-conductance mechanosensitive channel | 1.928514 | 0.000344 |
| **Gpt** | Xanthine-guanine phosphoribosyltransferase | 1.929255 | 0.00799 |
| **FhuA** | Ferric hydroxamate uptake | 1.931714 | 0.019246 |
| **UgpC** | ABC transporter | 1.935317 | 0.170665 |
| **AceF** | Acetyltransferase component of pyruvate dehydrogenase complex | 1.940297 | 0.000019 |
| **ArtM** | Arginine ABC transporter | 1.943326 | 0.085722 |
| **TusC** | Protein TusC | 1.946889 | 0.013199 |
| **YajD** | Cytoplasmic protein | 1.947354 | 0.022287 |
| **ArgI** | Ornithine carbamoyltransferase | 1.950222 | 0.012825 |
| **RplU** | 50S ribosomal protein L21 | 1.952338 | 0.00002 |
| **GJJ01_14600** | Alcohol dehydrogenase | 1.962454 | 0.137476 |
| **Rnt** | Ribonuclease T | 1.962753 | 0.006646 |
| **StpA** | DNA-binding protein | 1.969607 | 0.000048 |
| **TtcA** | tRNA-cytidine(32) 2-sulfurtransferase | 1.97273 | 0.000945 |
| **Lgt** | Phosphatidylglycerol--prolipoprotein diacylglyceryl transferase | 1.975106 | 0.012716 |
| **FeoB** | Ferrous iron transport protein B | 1.975771 | 0.000895 |
| **RpsS** | 30S ribosomal protein S19 | 1.983366 | 0.000233 |
| **AzoR_2** | FMN-dependent NADH-azoreductase | 1.98379 | 0.000285 |
| **RafA** | Alpha-galactosidase | 1.996071 | 0.024234 |
| **MetL** | Bifunctional aspartokinase/homoserine dehydrogenase | 1.997104 | 0.009795 |
| **SstT** | Serine/threonine transporter SstT | 1.997891 | 0.000088 |
| **RlmM** | Ribosomal RNA large subunit methyltransferase M | 2.011849 | 0.001095 |
| **RplW** | 50S ribosomal protein L23 | 2.019854 | 0.000029 |
| **LamB_3** | Maltoporin | 2.020749 | 0.010949 |
| **AdhE** | Aldehyde-alcohol dehydrogenase | 2.025335 | 0.000004 |
| **EntH** | Proofreading thioesterase EntH | 2.032558 | 0.00234 |
| **PotD** | Putrescine-binding periplasmic protein | 2.03493 | 0.035076 |
| **DnaK_1** | Chaperone protein DnaK | 2.040341 | 0.000008 |
| **RpsI** | 30S ribosomal protein S9 | 2.041983 | 0.000028 |
| **TatE** | Probable Sec-independent protein translocase protein TatE | 2.043726 | 0.000042 |
| **LpxP** | Lipid A biosynthesis palmitoleoyltransferase | 2.052455 | 0.185622 |
| **DppB_1** | ABC transporter permease subunit | 2.053847 | 0.221373 |
| **EPB11_22935** | GNAT family N-acetyltransferase | 2.059089 | 0.000213 |
| **MscS2** | Miniconductance mechanosensitive channel MscM | 2.059251 | 0.000485 |
| **RpiR** | MurR/RpiR family transcriptional regulator | 2.068097 | 0.126222 |
| **Nth** | Endonuclease III | 2.074001 | 0.009065 |
| **HemN_1** | Heme chaperone HemW | 2.081404 | 0.00926 |
| **ChbF_1** | 6-phospho-beta-glucosidase | 2.084432 | 0.044229 |
| **PflB** | Formate C-acetyltransferase | 2.093699 | 0.000552 |
| **FabD_1** | Malonyl CoA-acyl carrier protein transacylase | 2.122309 | 0.053347 |
| **CyoB** | Cytochrome bo(3) ubiquinol oxidase subunit 1 | 2.122529 | 0.000588 |
| **YgiQ** | UPF0313 protein B6R99_11700 | 2.124835 | 0.008092 |
| **TrxC** | Thioredoxin 2 | 2.134233 | 0.000725 |
| **ArgE** | Acetylornithine deacetylase | 2.135326 | 0.000114 |
| **CedA** | Cell division activator CedA | 2.140627 | 0.01736 |
| **YhbU_1** | Collagenase-like protease | 2.140923 | 0.002567 |
| **RuvC** | Crossover junction endodeoxyribonuclease RuvC | 2.141019 | 0.043143 |
| **MenC** | o-succinylbenzoate synthase | 2.144194 | 0.000414 |
| **YhjH_2** | Cyclic diguanylate phosphodiesterase | 2.145659 | 0.220157 |
| **YehS** | DUF1456 domain-containing protein | 2.151399 | 0.001293 |
| **GrcA** | Autonomous glycyl radical cofactor | 2.151728 | 0.000295 |
| **RpsK** | 30S ribosomal protein S11 | 2.173438 | 0.000091 |
| **Tsx** | Nucleoside-specific channel-forming protein Tsx | 2.176457 | 0.006869 |
| **RecJ** | Single-stranded-DNA-specific exonuclease RecJ | 2.177853 | 0.000886 |
| **CbiA** | Cobyrinate a,c-diamide synthase | 2.181772 | 0.00007 |
| **RafR** | HTH-type transcriptional regulator RafR | 2.194736 | 0.00092 |
| **CusR** | Copper response regulator transcription factor CusR | 2.19494 | 0.134761 |
| **GlpR_2** | DeoR faimly transcriptional regulator | 2.208174 | 0.00096 |
| **CysA_2** | Sulfate/thiosulfate import ATP-binding protein CysA | 2.209301 | 0.014194 |
| **OadA** | Oxaloacetate decarboxylase | 2.210083 | 0.000006 |
| **BL124_00024955** | Glycosyltransferase family 9 protein | 2.214218 | 0.036244 |
| **GJJ01_24875** | Phage tail tape measure protein | 2.216653 | 0.00087 |
| **YfiH_2** | Laccase domain protein yfiH | 2.217183 | 0.178853 |
| **RnhB** | Ribonuclease HII | 2.235692 | 0.006008 |
| **PheP** | Phenylalanine transporter | 2.251867 | 0.005591 |
| **NirD** | NirD protein | 2.262206 | 0.044901 |
| **FolB** | 7,8-dihydroneopterin aldolase | 2.267619 | 0.000018 |
| **FabZ** | 3-hydroxyacyl-[acyl-carrier-protein] dehydratase FabZ | 2.280111 | 0.000019 |
| **MnmC** | tRNA 5-methylaminomethyl-2-thiouridine biosynthesis bifunctional protein MnmC | 2.286753 | 0.007178 |
| **DmsA_2** | Anaerobic dimethyl sulfoxide reductase subunit A | 2.290555 | 0.031336 |
| **EptB** | Kdo(2)-lipid A phosphoethanolamine 7''-transferase | 2.295478 | 0.000504 |
| **MdtK** | Multidrug resistance protein MdtK | 2.296095 | 0.084468 |
| **HigA-2** | Antitoxin HigA-2 | 2.299354 | 0.016721 |
| **MrcB** | Penicillin-binding protein 1B | 2.319527 | 0.000048 |
| **GlrK** | Histidine kinase | 2.326 | 0.026569 |
| **DcuD** | Anaerobic C4-dicarboxylate transporter DcuC | 2.330103 | 0.052435 |
| **YbaB** | Nucleoid-associated protein B4U21_03855 | 2.330908 | 0.000027 |
| **ComR** | Bacterial regulatory proteins, tetR family | 2.334617 | 0.041113 |
| **HolA** | DNA polymerase III subunit delta | 2.340535 | 0.000107 |
| **CobS** | Adenosylcobinamide-GDP ribazoletransferase | 2.344313 | 0.004373 |
| **C2U49_27255** | LacI family DNA-binding transcriptional regulator | 2.347021 | 0.176025 |
| **YpjD** | CcsA-like protein | 2.349141 | 0.00297 |
| **TmcA** | GNAT family N-acetyltransferase | 2.354824 | 0.041393 |
| **BetP** | BetT protein | 2.361902 | 0.108794 |
| **Def** | Peptide deformylase | 2.373513 | 0.00035 |
| **RpsQ** | 30S ribosomal protein S17 | 2.395454 | 0.000435 |
| **PotA_3** | Spermidine/putrescine import ATP-binding protein PotA | 2.400163 | 0.048287 |
| **BdcA** | Oxidoreductase | 2.403073 | 0.000369 |
| **RpsO** | 30S ribosomal protein S15 | 2.424409 | 0.000452 |
| **AccD** | Acetyl-coenzyme A carboxylase carboxyl transferase subunit beta | 2.432996 | 0.000146 |
| **YgdR_2** | Membrane protein | 2.453397 | 0.225548 |
| **LysP** | Amino acid permease | 2.466425 | 0.000858 |
| **YdgI** | Amino acid permease | 2.530506 | 0.014464 |
| **DD581_11815** | Elongation factor Tu (Fragment) | 2.532635 | 0.001829 |
| **YfnB** | 2-haloalkanoic acid dehalogenase | 2.550547 | 0.005419 |
| **SoxR** | Redox-sensitive transcriptional activator SoxR | 2.553747 | 0.001327 |
| **PotB** | PotB protein | 2.557444 | 0.000292 |
| **Fiu** | Catecholate siderophore receptor Fiu | 2.572812 | 0.032033 |
| **HexR** | HexR protein | 2.584873 | 0.000539 |
| **Hfq** | RNA-binding protein Hfq | 2.631325 | 0.000398 |
| **B4U21_17300** | DUF2058 domain-containing protein | 2.633088 | 0.000054 |
| **YncA** | GNAT family N-acetyltransferase | 2.63699 | 0.008669 |
| **B4U21_16785** | Ion transporter | 2.659725 | 0.079696 |
| **TerB** | Tellurium resistance membrane protein TerB | 2.684546 | 0.004002 |
| **NuoH** | NADH-quinone oxidoreductase subunit H | 2.686746 | 0.001706 |
| **NuoM** | NADH dehydrogenase I subunit M | 2.698228 | 0.000095 |
| **HcaT** | 3-phenylpropionate MFS transporter | 2.713763 | 0.079902 |
| **UbiC** | Chorismate pyruvate-lyase | 2.724322 | 0.000129 |
| **BL124_00021250** | TonB-dependent receptor | 2.737865 | 0.043848 |
| **ExuR** | ExuR protein | 2.747183 | 0.013122 |
| **ArsC** | Arsenate reductase | 2.754157 | 0.000916 |
| **YfcB** | 50S ribosomal protein L3 glutamine methyltransferase | 2.874821 | 0.000505 |
| **NupX** | Nucleoside permease | 2.881831 | 0.001904 |
| **FhuB** | Fe(3+)-hydroxamate ABC transporter permease FhuB | 2.893739 | 0.009383 |
| **YifK** | Amino acid permease | 2.906191 | 0.000305 |
| **YhhV** | Antitoxin | 2.967039 | 0.003195 |
| **Fis** | DNA-binding protein Fis | 2.968407 | 0.002502 |
| **YhbU_3** | Ubiquinone biosynthesis protein UbiU | 2.968866 | 0.002548 |
| **YdhC** | Bcr/CflA family efflux transporter | 2.98384 | 0.102256 |
| **IscR** | HTH-type transcriptional regulator IscR | 3.004428 | 0.000048 |
| **MetE** | 5-methyltetrahydropteroyltriglutamate--homocysteine methyltransferase | 3.01663 | 0.012974 |
| **BL124_00033400** | UPF0149 protein BL124_00033400 | 3.027268 | 0.007692 |
| **WbaP** | UDP-Gal::undecaprenolphosphate Gal-1-P transferase | 3.03881 | 0.000118 |
| **NarK** | Nitrate/nitrite transporter | 3.081599 | 0.000368 |
| **LsrR** | LsrR, transcriptional repressor of lsr operon | 3.096841 | 0.002512 |
| **RfaH** | Transcription antitermination protein RfaH | 3.13163 | 0.006507 |
| **RbsC** | RbsC protein | 3.178122 | 0.001382 |
| **NemA** | N-ethylmaleimide reductase | 3.193323 | 0.000089 |
| **IsiB** | Flavodoxin | 3.214876 | 0.01251 |
| **Fim_1** | Fimbrial protein | 3.240534 | 0.004359 |
| **GlpC** | Anaerobic glycerol-3-phosphate dehydrogenase subunit C | 3.247958 | 0.000432 |
| **DeaD** | ATP-dependent RNA helicase DeaD | 3.266184 | 0.00001 |
| **HscB** | Co-chaperone protein HscB | 3.278164 | 0.001092 |
| **YncE_2** | Antigen Lp49 | 3.320257 | 0.001028 |
| **MenA** | 1,4-dihydroxy-2-naphthoate octaprenyltransferase | 3.337725 | 0.000074 |
| **RbsR** | Ribose operon transcriptional repressor RbsR | 3.339158 | 0.000405 |
| **BANRA_03067** | Amine oxidase, flavin-containing | 3.339239 | 0.066788 |
| **GJJ01_00155** | Acetyltransferase YafP | 3.374015 | 0.074881 |
| **Kup** | Low affinity potassium transport system protein kup | 3.428788 | 0.000123 |
| **TcyL** | Cystine ABC transporter | 3.546004 | 0.001191 |
| **Wza** | Integral outer membrane lipoprotein | 3.561825 | 0.092533 |
| **NudC** | NAD-capped RNA hydrolase NudC | 3.569819 | 0.169116 |
| **UbiJ** | Ubiquinone biosynthesis accessory factor UbiJ | 3.611191 | 0.000236 |
| **YhjX** | Inner membrane protein yhjX | 3.626972 | 0.039617 |
| **BVX91_07400** | Glycosyltransferase | 3.64601 | 0.114922 |
| **BrnQ** | Branched-chain amino acid transport system carrier protein | 3.686054 | 0.001326 |
| **RecN** | DNA repair protein RecN | 3.695094 | 0.00008 |
| **IlvB** | Acetolactate synthase | 3.81049 | 0.002032 |
| **DtpT** | Di-/tripeptide transporter | 3.873392 | 0.00004 |
| **AccB** | Biotin carboxyl carrier protein of acetyl-CoA carboxylase | 4.011628 | 0.000001 |
| **RecQ** | DNA helicase | 4.016875 | 0.000489 |
| **RcsA** | Transcriptional regulatory protein RcsA | 4.172624 | 0.002099 |
| **PriA** | Primosomal protein N' | 4.194054 | 0.003564 |
| **YaeE** | D-methionine ABC transporter permease MetI | 4.338075 | 0.000032 |
| **Hha** | Hemolysin expression modulating protein | 4.51068 | 0.016135 |
| **MenD** | 2-succinyl-5-enolpyruvyl-6-hydroxy-3-cyclohexene-1-carboxylate synthase | 4.880206 | 0.000359 |
| **XynB_2** | Beta-xylosidase | 4.880335 | 0.000385 |
| **PlaP** | APC family permease | 4.992996 | 0.000231 |
| **LptB_3** | ABC transporter ATP-binding protein | 5.02985 | 0.002172 |
| **MalP_2** | Alpha-1,4 glucan phosphorylase | 5.062167 | 0.001254 |
| **DnaE** | DNA polymerase III subunit alpha | 5.155331 | 0.000613 |
| **GlpB** | Anaerobic glycerol-3-phosphate dehydrogenase subunit B | 5.210341 | 0.001593 |
| **BtuF** | Vitamin B12-binding protein | 5.350788 | 0.000078 |
| **YahK** | Alcohol dehydrogenase catalytic domain-containing protein | 5.554869 | 0.002519 |
| **Fsa** | Fructose-6-phosphate aldolase | 5.782794 | 0.000532 |
| **CadB** | Arginine/agmatine antiporter | 5.991591 | 0.003256 |
| **YbbM** | ABC-type uncharacterized transport system, permease component | 6.000384 | 0.000427 |
| **ZnuC** | Zinc import ATP-binding protein ZnuC | 6.058259 | 0.032729 |
| **C4Y50_024620** | Cobalamin biosynthesis protein CobW | 6.2951 | 0.036742 |
| **CsdE** | Cysteine desulfurase sulfur acceptor subunit CsdE | 6.756937 | 0.001824 |
| **MoaD** | MoaD protein | 6.87882 | 0.000197 |
| **YneE** | Ibestrophin | 6.958729 | 0.000014 |
| **YaeJ** | Aminoacyl-tRNA hydrolase | 6.988485 | 0.000529 |
| **MsbA_1** | Cysteine/glutathione ABC transporter ATP-binding protein/permease CydC | 7.049462 | 0.00007 |
| **DusB** | tRNA-dihydrouridine synthase B | 7.173647 | 0.000535 |
| **Tam** | Trans-aconitate 2-methyltransferase | 7.525914 | 0.000184 |
| **GarR_1** | 2-hydroxy-3-oxopropionate reductase | 8.224097 | 0.000041 |
| **YhbT** | Ubiquinone biosynthesis accessory factor UbiT | 9.443405 | 0.00019 |
| **YejM** | Inner membrane protein YejM | 9.547558 | 0.000043 |
| **DinG_1** | ATP-dependent helicase | 9.985891 | 0.000444 |
| **YgfY** | FAD assembly factor SdhE | 10.655565 | 0.000011 |
| **SecE** | Protein translocase subunit SecE | 11.556173 | 0.000652 |
| **YbeD** | UPF0250 protein B4U21_05195 | 11.970744 | 0.000095 |
| **FrmA_1** | S-(hydroxymethyl)glutathione dehydrogenase | 12.041462 | 0 |
| **B4U21_17310** | Cold-shock' DNA-binding domain | 13.584165 | 0.000129 |
| **YijO** | Helix-turn-helix domain-containing protein | 14.891831 | 0.000485 |
| **RpmI** | 50S ribosomal protein L35 | 211.055101 | 0.000001 |

| **Origin** | **Add Na^+^** | **Add HPO_3_^¯^** | **Lose HPO_3_^¯^** | **Add 1 Ara4N and lose HPO_3_^¯^** | **Add 1 Ara4N** | **Add 1 Ara4N and add Na^+^** | **Add 2 Ara4N** | **Add 2 Ara4N and add Na^+^** | **Add 1 pEtN** | **Add 1 pEtN and lose HPO_3_^¯^** |
| --- | --- | --- | --- | --- | --- | --- | --- | --- | --- | --- |
| **1797 (P1)** | ND | ND | 1717 | 1848 | 1928 | ND | 2059 | ND | ND | ND |
| **1825 (P2)** | 1847 | ND | 1745 | 1876 | 1956 | 1978 | 2087 | 2109 | 1947 | 1867 |
| **1841 (P3)** | ND | 1921 | 1761 | 1892 | ND | ND | 2103 | ND | ND | ND |

**Supplementary Table 6.** Lipid A modifications presented in this study (*m/z*).

**ND,** Not detected

**Supplementary Table 7.** Chemical structures of lipid A presented in this study.

| ***m/z*** | **structure** |
| --- | --- |
| **1717** |  |
| **1797** |  |
| **1848** |  |
| **1928** |  |
| **2059** |  |
| **1745** |  |
| **1825** |  |
| **1847** | Na^+^ |
| **1876** |  |
| **1956** |  |
| **1978** |  Na^+^ |
| **2087** |  |
| **2109** |  Na^+^ |
| **1947** | or  |
| **1867** | or  |
| **1841** |  |
| **1921** | or  |
| **1761** |  |
| **1892** |  |
| **2103** |  |
